# Enabling climate action: A nexus approach for delivering co-benefits across biodiversity, water, food, and health

**DOI:** 10.1016/j.isci.2026.117734

**Published:** 2026-10-05

**Authors:** Pramod K. Singh, Himangana Gupta, Julio Díaz-José, Pamela D. McElwee, Maria Cristina Tirado, Francesco Cherubini, Helena Duchková, Marcos Llope, Mariana M. Vale, Shabana Khan, Akihiko Ito, Masakazu Hori, Siri H. Eriksen, Lakshmikant Tiwari, Sui Chian Phang, Daniel Mason-D’Croz, Xiyan Xu, Tiff van Huysen, Mario Herrero, Taylor H. Ricketts, Paula A. Harrison, Pete Smith

**Affiliations:** 1 Institute of Rural Management Anand (IRMA), “Tribhuvan” Sahkari University, Anand, India; 2 United Nations University - Institute for the Advanced Study of Sustainability, Tokyo, Japan; 3 Faculty of Agriculture and Biological Sciences, Universidad Veracruzana, Xalapa, Mexico; 4 School of Environmental and Biological Sciences, Rutgers University, Rutgers, NJ, USA; 5 School of Public Health, Institute of the Environment and Sustainability, University of California, Los Angeles, Los Angeles, CA, USA; 6 Industrial Ecology Programme, Department of Energy and Process Engineering, Norwegian University of Science and Technology, Trondheim, Norway; 7 Global Change Research Institute of the Czech Academy of Sciences, Brno, Czech Republic; 8 Faculty of Humanities, Charles University in Prague, Prague, Czech Republic; 9 Centro Oceanográfico de Gijón/Xixón, Instituto Español de Oceanografía (IEO-CSIC), Gijón/Xixón, Asturies, Spain; 10 Department of Ecology, Federal University of Rio de Janeiro, Rio de Janeiro, Brazil; 11 Indian Research Academy, Delhi, India; 12 Graduate School of Agricultural and Life Sciences, the University of Tokyo, Tokyo, Japan; 13 Fisheries Resources Institute, Japan Fisheries Research and Education Agency, Yokohama, Japan; 14 Faculty of Landscape and Society, Norwegian University of Life Sciences, Ås, Norway; 15 The Nature Conservancy, London, UK; 16 Department of Global Development, College of Agriculture and Life Sciences, Cornell University, Ithaca, NY, USA; 17 Agricultural Economics and Rural Policy Group, Wageningen University and Research, Wageningen, the Netherlands; 18 State Key Laboratory of Earth System Numerical Modeling and Application, Institute of Atmospheric Physics, Chinese Academy of Sciences, Beijing, China; 19 Intergovernmental Science-Policy Platform on Biodiversity and Ecosystem Services (IPBES), Bonn, Germany; 20 Gund Institute for Environment, University of Vermont, Burlington, VT, USA; 21 Rubenstein School for Environment and Natural Resources, University of Vermont, Burlington, VT, USA; 22 Department of Global Development, College of Agriculture and Life Sciences and Cornell Atkinson Center for Sustainability, Cornell University, Ithaca, NY, USA; 23 UK Centre for Ecology & Hydrology, Lancaster, UK; 24 Institute of Biological & Environmental Sciences, University of Aberdeen, Aberdeen, UK

**Keywords:** climate action, nexus governance, Earth System Justice, co-benefits and trade-offs, indigenous and local knowledge, transformative change

## Abstract

Climate response options rarely affect biodiversity, water, food, and health in uniform ways. This comparative analysis examines why technically viable response options generate uneven cross-nexus outcomes, and how governance and justice conditions shape whether these outcomes become durable co-benefits. Anchored in the IPBES Nexus Assessment, the analysis synthesizes evidence from fifteen response options spanning land, ocean, energy, urban, industrial, and societal transitions. Ecosystem-centered and demand-side pathways more consistently produce synergies because they work through ecological functions that connect several nexus elements. Infrastructure- and technology-centered options deliver mitigation and health gains, but their wider effects are more context-dependent and often shaped by land use, water demand, biodiversity risk, tenure, access, and benefit-sharing. These patterns do not arise from technical design alone. By integrating nexus governance with Earth System Justice, the analysis indicates how cross-sectoral coordination, distributive equity, procedural inclusion, accountable finance, and knowledge pluralism can shape legitimacy and durability. Climate action is more likely to deliver sustained system-wide benefits when institutions align ecological interdependencies with social rights and responsibilities. Otherwise, promising interventions may remain partial, contested or short-lived.

## Highlights

• Fifteen climate options yield uneven biodiversity, water, food, and health outcomes
• Ecosystem-centered and demand-side pathways yield stronger nexus synergies
• Infrastructure outcomes depend on siting, tenure, and benefit-sharing
• Inclusive nexus governance and Earth System Justice sustain durable co-benefits

## Graphical abstract

**Figure:**
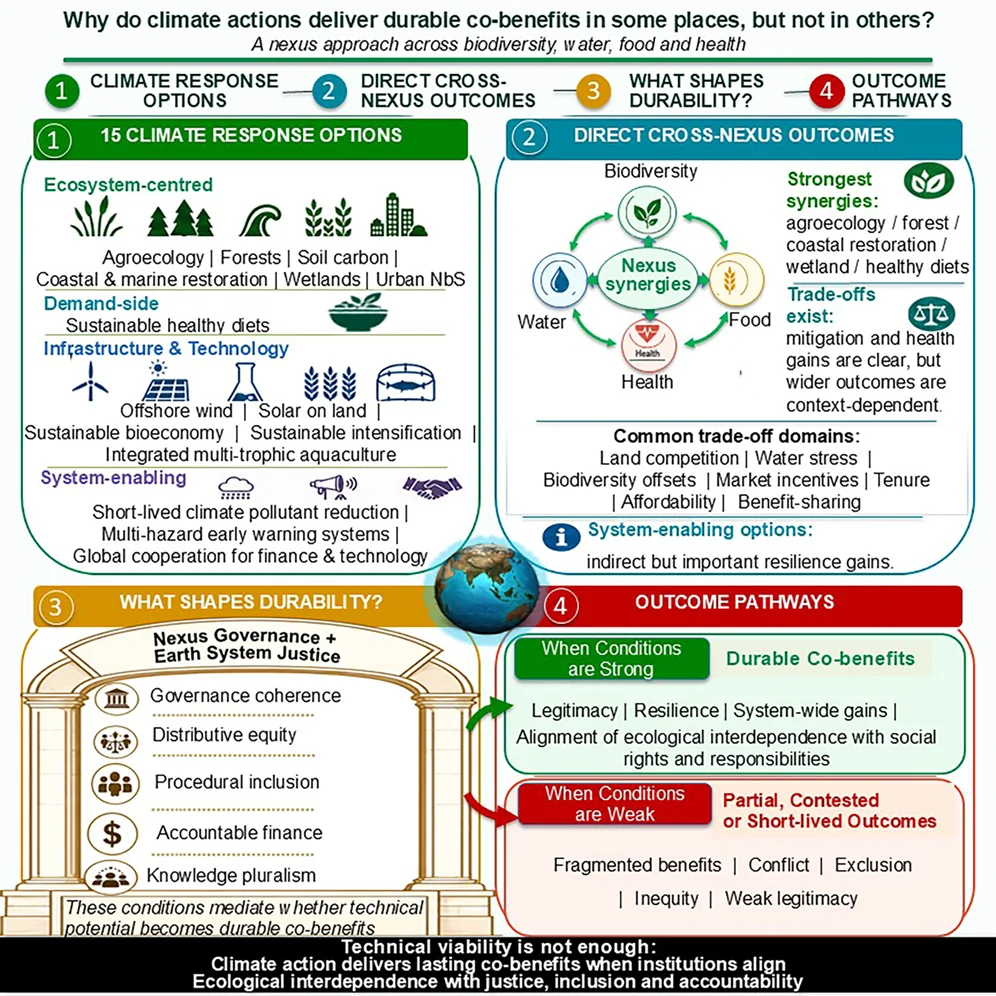

## Teaser

Environmental science; Environmental policy; Nature conservation; Ecology

## Introduction

Climate change unfolds across ecological, hydrological, social, and economic systems. It intensifies pressures on biodiversity, freshwater resources, food and nutrition security, and public health. The Sixth Assessment Report of the Intergovernmental Panel on Climate Change identifies climate change as a threat multiplier that deepens existing vulnerabilities in societies shaped by inequality and institutional fragility.^1^

These pressures interact through reinforcing feedback across sectors and scales. Climate risk is therefore not confined to a single domain. Climate change both results from and accelerates unsustainable land use, biodiversity loss, freshwater depletion, and emissions from agriculture, energy, and health systems.^2^^,^^3^ Intact ecosystems moderate climatic extremes, regulate hydrological cycles, sustain food production, and protect human health. Degradation weakens these buffering functions and heightens exposure. This interdependence underpins the nexus perspective, which conceptualizes biodiversity, water, food, health, and climate as coupled systems shaping development and risk trajectories.^4^^,^^5^

We use the term nexus approach to refer to an integrated way of analyzing interlinkages and interdependencies among sectors and systems. In the Thematic Assessment Report on the interlinkages among biodiversity, water, food and health (“nexus assessment”) of the Intergovernmental Science-Policy Platform on Biodiversity and Ecosystem Services (IPBES), this approach supports adaptive decisions that seek to maximize synergies and minimize trade-offs.^5^ The relevant nexus for this article links biodiversity, water, food, health, and climate change. Climate change is included because it interacts with the other nexus elements both as a driver of risk and through adaptation and mitigation actions.^5^ We therefore assess climate response options by asking how their effects move across biodiversity, water, food, and health systems. Interlinkages denote influences and interactions among multiple elements. Interdependencies arise when one or more elements depend on another to function. Synergies occur when improvement in one element enhances another. Trade-offs occurs when improvement in one element worsens another. This framing distinguishes nexus analysis from a siloed approach, where decisions are made without sufficient attention to cross-system consequences.^5^

We use transformation to describe system-level change across institutions, practices, technologies, economies, and values. We use transformative change more specifically, following the IPBES Thematic Assessment Report on the underlying causes of biodiversity loss and the determinants of transformative change and options for achieving the 2050 vision for biodiversity (“transformative change assessment”), to mean fundamental, system-wide reorganization across technological, economic, and social factors, including paradigms, goals, and values.^6^ The IPBES transformative change assessment further clarifies that deliberate transformative change requires shifts in views, structures, and practices that address the underlying causes of biodiversity loss and nature’s decline.^6^ Views are ways of thinking, knowing, and seeing. Structures are ways of organizing, regulating, and governing. Practices are ways of doing, behaving, and relating.^6^ In this article, a climate response option has transformative potential only when it moves beyond technical improvement and helps alter the institutional, social, and ecological relations that produce unsustainable or unjust outcomes.

Recognition of systemic interdependence has not yet produced sufficiently integrated climate action. Mitigation pathways remain misaligned with long-term temperature goals, while adaptation measures often lack institutional coherence.^7^ Prevailing development models continue to separate ecological integrity from social well-being.^6^ Political economy constraints, entrenched interests, and persistent financing gaps further restrict coordinated responses.^8^^,^^9^ Many interventions still privilege technical optimization, with less consistent attention to cross-system interactions, distributive safeguards, and knowledge pluralism.

Extensive scholarship evaluates climate interventions and documents their mitigation potential and sector-specific co-benefits.^5^^,^^10^^,^^11^ The IPBES Nexus Assessment synthesizes interactions among biodiversity, water, food, health, and climate change, and shows that response options vary widely in their nexus-wide impacts.^5^ Yet similar options can produce durable synergies in some settings and contested or partial outcomes in others. This unevenness is not a secondary implementation detail. It is the central analytical problem. The remaining explanatory gap concerns how technical potential becomes, or fails to become, a durable, system-wide benefit under specific governance and justice conditions. Without such clarity, policy design may privilege mitigation efficiency while neglecting ecological integrity, distributive justice and long-term institutional resilience. This analysis therefore asks: why do technically viable climate response options generate uneven cross-nexus outcomes, and how do governance and justice conditions shape whether these outcomes become durable co-benefits across biodiversity, water, food, and health systems? The question has two linked tasks. First, the analysis identifies recurring patterns of synergies, trade-offs, and mixed effects across selected response options. Second, it examines how governance coherence, distributive equity, procedural inclusion, accountable finance, and knowledge pluralism shape the durability and legitimacy of those outcomes.

This paper does not extend the catalog of climate response options. Its contribution lies in re-analyzing a bounded assessment evidence base through an integrated nexus governance and Earth System Justice (ESJ) framework. The analysis shifts attention from whether response options can generate co-benefits to why comparable options produce durable synergies in some settings and fragmented, contested, or short-lived outcomes in others.

This distinction matters for technology-intensive mitigation as well as ecosystem-centered action. Recent work on carbon capture, utilization and storage, geological storage capacity, and wind-solar-hydro complementarity shows that investment viability and decarbonization potential vary with source-sink matching, plant-level location, policy incentives, and spatial optimization.^12^^,^^13^^,^^14^^,^^15^ These studies reinforce the need to evaluate technical options together with finance, siting, institutional capacity, and distributional consequences.

The analysis integrates two complementary lenses. Nexus governance examines institutional arrangements that coordinate across sectors and scales, reduce trade-offs, and realize synergies.^5^ ESJ situates distributive equity, procedural fairness, intergenerational responsibility, and the recognition of diverse knowledge systems within Earth system governance.^16^ Together, these perspectives extend evaluation beyond technical effectiveness toward coherence, legitimacy, and durability.

Figure 1 summarizes the analytical sequence. It separates the direct nexus effects of climate response options from the enabling and mediating conditions that shape whether those effects become durable cross-nexus benefits or remain fragmented, contested, or short-lived.

**Figure 1:**
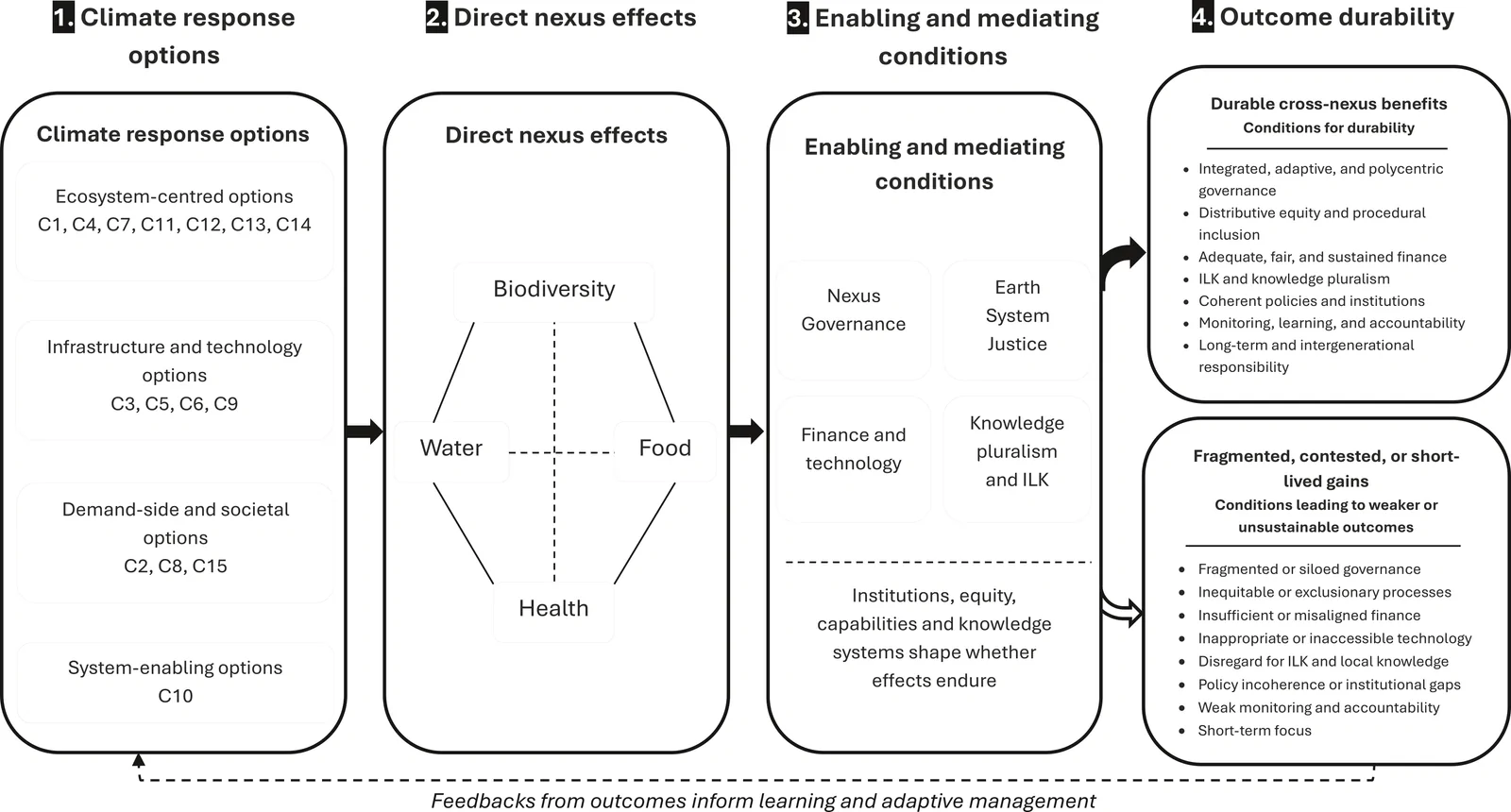
Analytical framework linking climate response options, cross-nexus outcomes, governance-and-justice conditions, and outcome durability The framework treats synergies, trade-offs and mixed effects across biodiversity, water, food and health systems as the outcomes to be explained. Governance coherence, distributive equity, procedural inclusion, accountable finance, and knowledge pluralism are treated as mediating conditions that shape whether technical potential becomes a durable system-wide benefit or remains fragmented, contested, or short-lived.

## Results

### Climate change response options: Co-benefits, trade-offs, and structural differentiation

#### Cross-nexus performance of climate response options

This section compares fifteen climate change response options across biodiversity, water, food, and health systems. Its purpose is diagnostic. It identifies recurring patterns of synergies, trade-offs, and mixed effects before examining the governance and justice conditions that shape durability. A climate change response option refers here to an evidence-based ecological, technological, institutional, financial, or socio-cultural intervention assessed for cross-nexus performance and compatibility with ESJ and nexus governance principles. The analysis treats observed patterns as evidence-supported comparative tendencies. It does not treat them as intrinsic rankings of the quality of response options. Standardized definitions appear in Table S1, and observed cross-domain impacts are synthesized in Table 1. Table S11 reports the evidence base, geographical spread, confidence level, and key limitation for each response option.

**Table 1:** Observed co-benefits and trade-offs across climate response options

Response option	Co-benefits				Typical trade-off domain	Key sources (APA, from uploaded materials)
	Biodiversity	Water	Food	Health		
C01 Increase soil organic carbon	++	+	++	+	permanence of sequestration	Cheng et al.^17^; Keesstra et al.^18^; De Deyn and Kooistra^19^; Smith et al.^20^
C02 Sustainable intensification	+	±	++	+	input dependence, rebound effects	Bais-Moleman et al.^21^; Brauman et al.^22^; Garrett et al.^23^; Smith et al.^24^
C03 Integrated multitrophic aquaculture	+	+	+	+	local ecological limits	Chopin et al.^25^; FAO^26^; Hori et al.^27^; Gentry et al.^28^
C04 Wetland conservation and restoration	++	++	+	+	land use, vector exposure	Munang et al.^29^; Dale and Connelly^30^; Sadeghi et al.^31^; Smith et al.^20^^,^^24^
C05 Offshore wind power	±	0	0	+	marine ecosystem disturbance	Singh et al.^10^; Smith et al.^11^
C06 Solar photovoltaics on land	±	0	0	+	land competition	Singh et al.^10^; Smith et al.^11^
C07 Sustainable bioeconomy	±	±	+	+	biomass demand	IPBES^32^; Singh et al.^10^; Smith et al.^11^
C08 Reduce short-lived climate pollutants	+	+	0	++	sectoral coverage	IPCC^3^; Smith et al.^11^
C09 Multi-hazard early warning systems	0	+	+	++	coverage and access	IPBES^32^; Singh et al.^10^; Smith et al.^11^
C10 Global cooperation for finance and technology	+	+	+	+	distributional equity	IPBES^32^; Singh et al.^10^; Smith et al.^11^
C11 Agroecology	++	+	++	+	yield variability	Bharucha et al.^33^; Duddigan et al.^34^; Khadse & Rosset^35^; Jaacks et al.^36^
C12 Forest-based practices to address climate change	++	+	+	+	tenure and access	Blackman & Veit^37^; Duchelle et al.^38^; Gebara & Agrawal^39^; Garcia et al.^40^
C13 Restoration of coastal and marine ecosystems for carbon sequestration	++	++	+	+	local livelihood trade-offs	Wylie et al.^41^; Huff & Tonui^42^; Formuli et al.^43^
C14 Urban nature-based solutions	+	+	0	++	spatial equity	Anguelovski et al.^44^; Garcia Ferrari et al.^45^; Reynolds et al.^46^
C15 Sustainable healthy diets	++	+	++	++	affordability and access	Springmann et al.^47^; Singh et al.^10^

Note: symbols indicate the direction and consistency of direct or near-direct nexus effects reported in the assessed evidence: ++ consistently positive; + mostly positive; 0 neutral or indirect; ± mixed or context-dependent. They do not represent effect sizes, universal outcomes, or invariant effects across regions and time horizons. Where evidence varies by context, the table reports mixed or context-dependent effects rather than forcing a single positive or negative classification. Citations are limited to sources explicitly available in the uploaded assessment chapters and synthesis papers.

Three patterns are most visible. First, ecosystem-centered and demand-side options exhibit the strongest cross-nexus synergies. Agroecology (C11), wetland conservation, and restoration (C04), forest-based practices (C12), coastal and marine ecosystem restoration (C13), and sustainable healthy diets (C15) show predominantly positive biodiversity-food interactions, alongside consistent gains in water regulation and health.^10^^,^^11^ These options simultaneously reinforce the ecological functions that underpin several nexus domains. Soil formation, nutrient cycling, hydrological regulation, and dietary diversification operate as integrative processes rather than isolated interventions. Increasing soil organic carbon (C01) shows a similar profile: biodiversity, agricultural productivity, and water-retention benefits are widely reported, although the permanence of sequestration remains a recognized constraint.^17^^,^^18^^,^^20^ Sustainable healthy diets (C15) also show consistent synergies across biodiversity, food security, and health.^10^^,^^47^ Its main trade-offs concern affordability and cultural transition rather than ecological contradiction.

Second, infrastructure- and technology-centerd mitigation options display more differentiated outcomes. Offshore wind power (C05) and solar photovoltaics on land (C06) deliver clear mitigation and health gains through energy decarbonization and reduced air pollution.^10^^,^^11^ Their biodiversity and food-system interactions are more variable. Reported trade-offs concern marine ecosystem disturbance and competition for land, arising from spatial allocation rather than from incompatibility with mitigation objectives. The sustainable bioeconomy (C07) follows a comparable pattern. It can support food and health outcomes, but its effects on biodiversity and water remain context-dependent because biomass demand can intensify pressure on land and water.^5^^,^^10^ Thus, outcomes depend on feedstock sourcing, land governance, and ecological thresholds.

Third, system-enabling options generate mediated but important cross-nexus effects. Multi-hazard early warning systems (C09) strengthen health and food-system resilience through anticipatory action,^10^^,^^11^ although biodiversity effects are usually mediated effects rather than direct effects. Reducing short-lived climate pollutants (C08) yields rapid climate and health gains because methane and black carbon have distinct atmospheric properties.^3^ Global cooperation for finance and technology (C10) functions as a structural enabler across domains. Positive impacts are reported across biodiversity, water, food, and health, but distributional equity remains a recurrent concern.^10^^,^^11^

Across categories, trade-offs cluster around land competition, biomass demand, tenure and access rights, affordability, and distributional equity. Mixed outcomes do not imply net failure. They reveal structural tensions within socio-ecological systems that need to be governed rather than ignored.

#### Justice alignment and transformative differentiation

Figure 2 differentiates response options based on their alignment with the scoring criteria for social inclusion, equity, and transformative change. It does not rank sectors. Instead, it maps normative and institutional orientation, showing how justice compatibility and systemic integration vary independently of technological ambition.

**Figure 2:**
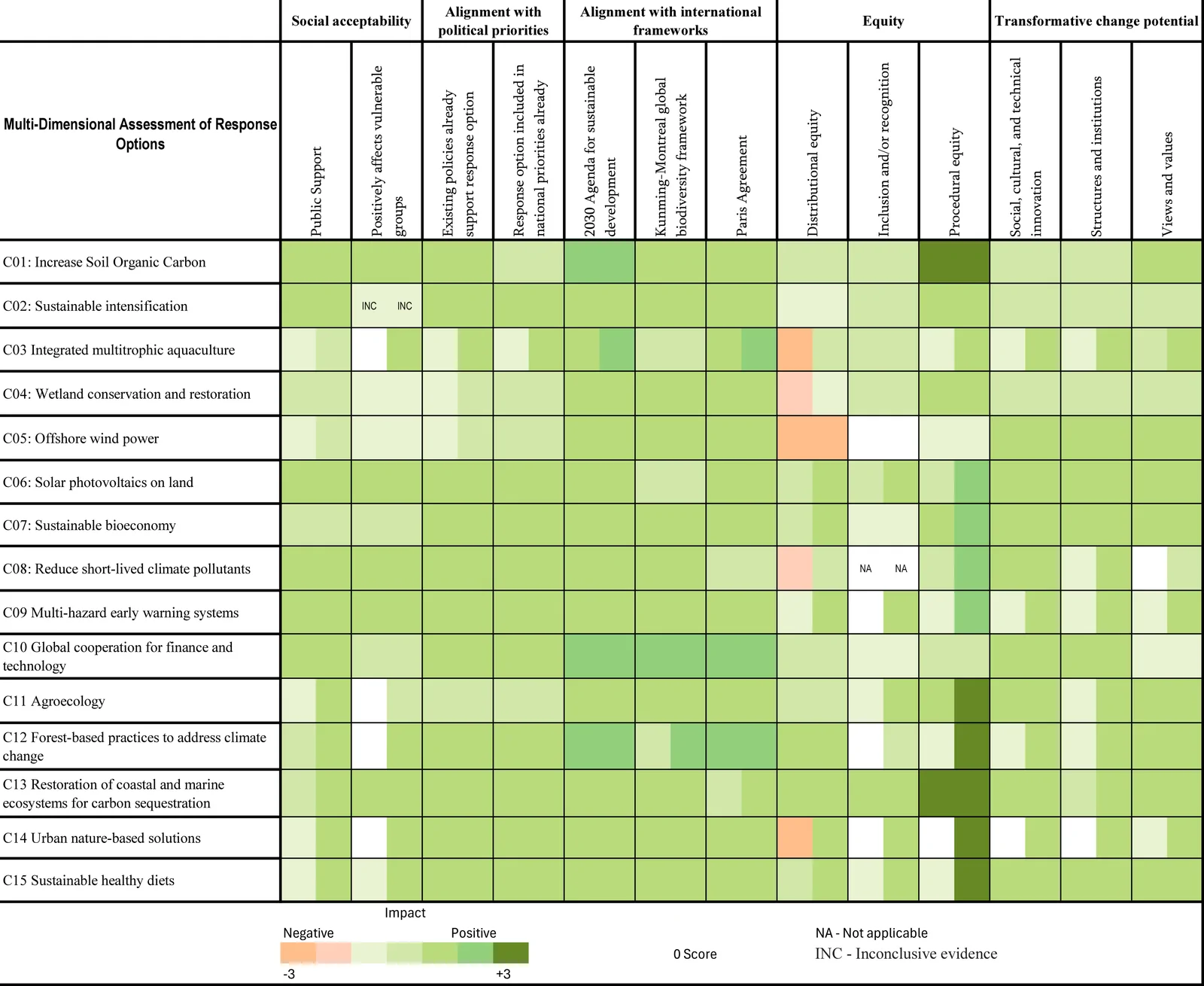
Multi-dimensional assessment of response options using social, equity, and transformative change criteria (modified from ref.^10^) Green indicates synergies; orange indicates trade-offs. Light, medium, and dark shading indicate small, moderate, and large impacts, respectively.

Ecosystem-centered and demand-side options cluster toward stronger ESJ compatibility and nexus governance synergy. Agroecology (C11), forest-based practices (C12), coastal and marine ecosystem restoration (C13), and sustainable healthy diets (C15) occupy this zone.^10^^,^^11^ These pathways embed distributive co-benefits within production and consumption systems. They strengthen local autonomy while integrating biodiversity, water, food, and health linkages. Their transformative character lies in reshaping land-livelihood-governance relationships and embedding plural knowledge systems in decision-making.^6^^,^^16^

Wetland conservation and restoration (C04) and integrated multitrophic aquaculture (C03) display similar systemic promise. Both can deliver broad cross-nexus benefits. Their justice performance, however, depends on participatory governance and ecological safeguards. Where inclusion and accountability are institutionalized, synergies consolidate; where safeguards weaken, benefits fragment.^5^

Infrastructure-centered mitigation pathways occupy a distinct position. Offshore wind (C05) and land-based solar photovoltaics (C06) provide clear mitigation and health gains.^10^^,^^11^ Figure 2 indicates more moderate justice alignment for these options. Distributive outcomes hinge on siting, tenure, and benefit-sharing design. Without procedural safeguards, benefits may concentrate while ecological or social burdens diffuse. Technical maturity does not remove this institutional dependence.^16^

The sustainable bioeconomy (C07) demonstrates high systemic potential, but its justice profile varies with biomass sourcing, land governance, and value-chain structure. Circular resource use does not automatically secure equity. Institutional design determines whether local livelihoods strengthen or extraction pressures intensify.^5^^,^^10^

System-enabling options reveal a further distinction. Reducing short-lived climate pollutants (C08) yields rapid climate and health benefits due to the atmospheric properties of methane and black carbon.^3^ Multi-hazard early warning systems (C09) strengthen resilience through anticipatory action.^10^^,^^11^ Both show strong relevance to equity in Figure 2. Their transformative depth depends on whether they expand rights to information, reach marginalized groups, and induce institutional reform in risk governance.^6^^,^^16^

Global cooperation for finance and technology (C10) is structurally distinct because it conditions feasibility and justice outcomes across the set of options. It scores highest on ESJ compatibility and nexus governance synergy.^10^ Its transformative potential lies in redistributing capabilities and correcting structural asymmetries in climate governance.^6^^,^^16^ Without equitable finance and technology transfer, cross-nexus gains remain uneven.

### Enabling climate action: Governance, finance, equity, and knowledge

As climate change and biodiversity loss intensify, mitigation, and adaptation depend not only on limiting adverse outcomes but also on the institutions, power relations, and socio-political contexts through which strategies are enacted. Enabling conditions include governance structures, financial and technological mechanisms, ethical frameworks, justice considerations, and the weaving of indigenous and local knowledge (ILK) with scientific knowledge. Together, these conditions shape whether response options generate durable co-benefits or reproduce trade-offs across biodiversity, water, food, health, and climate systems.

#### Governance and institutional frameworks for enabling climate action

This subsection examines how institutional arrangements shape coordination, legitimacy, and durability. It focuses on actors, policy instruments and governance frameworks rather than cataloging governance models.

Nexus governance offers a more integrative and transdisciplinary framing of climate challenges and responses. It brings diverse actors into multiple arenas of engagement, makes values such as equity and accountability explicit, enables policy alignment, and supports experimental, adaptive, and reflexive action.^32^^,^^48^ Governance, therefore, does more than implement climate action. It shapes how mitigation and adaptation unfold and whether they contribute to sustainable development across environmental and societal dimensions.

Climate governance operates through three interrelated elements: (1) actors and institutions working in diverse arenas of engagement; (2) policy instruments, including regulations, economic tools, and procedural mechanisms that coordinate climate action across levels and sectors^10^^,^^49^^,^^50^^,^^51^^,^^52^; and (3) governance frameworks and mechanisms that structure actor interactions and define how authority is organized.^53^^,^^54^

Figure 3 conceptualizes these governance approaches. It shows how actors and institutions interact through regulatory, market-based, and voluntary instruments within broader governance frameworks. Polycentric governance distributes authority across overlapping centers of decision-making, allowing context-specific responses and adaptive learning.^54^^,^^55^ Nexus governance complements this by integrating policies across traditionally siloed sectors and supporting equitable, cross-sectoral co-benefits.^32^^,^^48^

**Figure 3:**
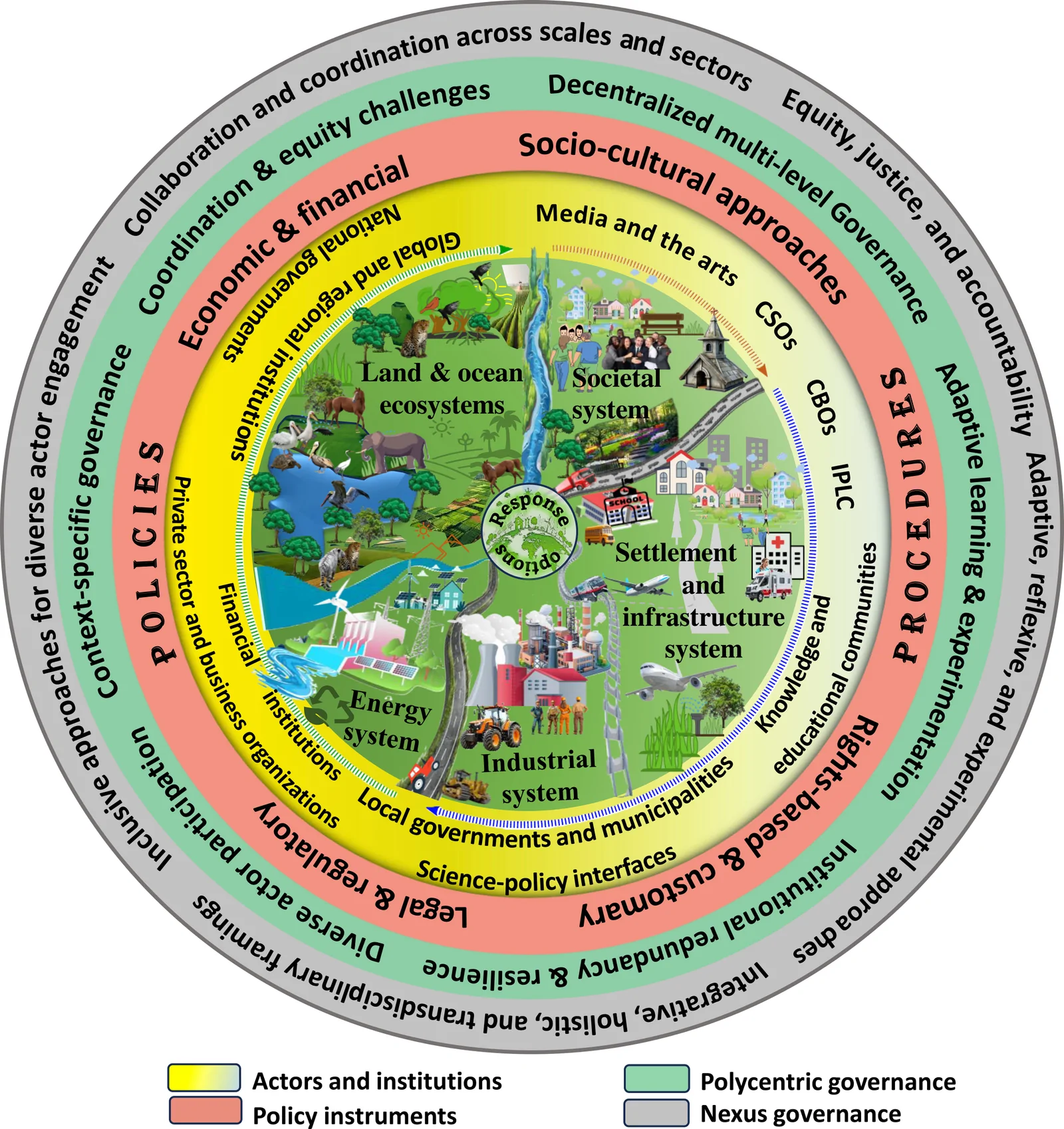
Governance approaches for climate action (systems transitions component modified from ref.^50^) The figure links actors and institutions, policy instruments, and governance frameworks. It shows how polycentric and nexus governance can support coordination, safeguards, and adaptive learning across sectors and scales.

##### Key actors and institutions

Variation in outcomes across climate change response options reflects differences in how actors are positioned, empowered, and connected within governance systems. Actors engage in decision-making across multiple arenas of engagement, understood as the settings and contexts in which governance interactions shape nexus-oriented approaches and initiatives.^50^ These arenas span political, economic, ecological, socio-cultural, and knowledge-technological domains. Power relations within them are often asymmetric. Hence, each arena contributes distinct perspectives and capacities to climate action.^50^ Table 2 summarizes the major categories of actors and their areas of engagement across the 15 selected climate response options.

**Table 2:** Response options, key actors, and arenas of engagement

Response options	Actors	Arena of engagement
C01 Increase soil organic carbon	agriculture ministries; extension systems; farmer organisations; rural development agencies; microfinance institutions; research institutes; civil society; IPLCs	ecological; socio-cultural; political; economic; technological
C02 Sustainable intensification	agriculture ministries; agribusiness firms; development banks; research institutes; farmer producer organisations; regulators	economic; technological; political; ecological
C03 Integrated multitrophic aquaculture	fisheries authorities; aquaculture enterprises; coastal municipalities; marine research centers; coastal community organisations; environmental NGOs	ecological; economic; technological; socio-cultural; political
C04 Wetland conservation and restoration	environment ministries; river basin authorities; municipal agencies; conservation organisations; community groups; IPLCs	ecological; political; socio-cultural; economic
C05 Offshore wind power	energy agencies; renewable companies; marine spatial planners; development banks and private investors; regulators; science-policy advisers	economic; technological; political; ecological
C06 Solar photovoltaics on land	energy regulators; solar firms; green banks; municipalities; community energy initiatives; technical institutes	economic; technological; political; socio-cultural; ecological
C07 Sustainable bioeconomy	industry and environment ministries; bio-based enterprises; innovation hubs; financiers; research institutes; civil society	economic; technological; political; ecological
C08 Reduce short-lived climate pollutants	environment and health ministries; sector regulators; clean-technology firms; civil society; local governments; media and communication platforms	political; technological; socio-cultural; economic; ecological
C09 Multi-hazard early warning systems	meteorological services; disaster authorities; climate centers; health agencies; local governments; community organisations	technological; political; socio-cultural; ecological; economic
C10 Global cooperation for finance and technology	multilateral climate institutions; development banks; finance ministries; climate finance units; technology platforms; science-policy interfaces; civil society oversight bodies	political; economic; technological
C11 Agroecology	agriculture ministries; agroecology networks; farmer movements; women’s collectives; research institutes; civil society; IPLCs	ecological; socio-cultural; political; economic; technological
C12 Forest-based practices to address climate change	forest ministries; community forest organisations; carbon-market actors; conservation NGOs; IPLCs; financiers	ecological; political; economic; socio-cultural; technological
C13 Restoration of coastal and marine ecosystems for carbon sequestration	coastal authorities; blue-carbon initiatives; development finance; municipalities; fisheries cooperatives; IPLCs; environmental NGOs; research institutes	ecological; political; economic; socio-cultural; technological
C14 Urban nature-based solutions	municipal governments; planning departments; health agencies; urban ecology institutes; neighborhood associations; real estate and green-infrastructure firms	political; socio-cultural; ecological; economic; technological
C15 Sustainable healthy diets	health and agriculture ministries; food research institutes; civil society; youth movements; food enterprises; media; community networks	socio-cultural; political; economic; technological; ecological

Note: arena categories indicate the main settings through which actors shape implementation.IPLCs, Indigenous Peoples and local communities; NGOs, non-governmental organizations; PV, photovoltaics; REDD+, reducing emissions from deforestation and forest degradation.

Equity, inclusivity, plural values, and deliberative approaches within these arenas are critical for addressing systemic failures that undermine climate action.^10^^,^^49^ Diverse actors require governance arrangements that facilitate coordination, deliberation, and negotiation across scales. National governments continue to dominate formal policy processes, but non-state actors, including civil society organizations and private firms, shape climate action through practice and influence. They design local initiatives, steer public debate, and direct flows of finance and technical expertise.^5^

##### Policy instruments for effective climate action

Policy instruments translate governance intent into practice. Their design conditions whether climate responses reinforce cross-sectoral synergies or reproduce fragmentation. Regulatory tools, such as carbon taxes and emissions caps, set binding limits. Market-based options, including emissions trading and payments for ecosystem services, use financial signals to influence decisions.^56^ Voluntary commitments, from corporate net-zero pledges to community-driven conservation initiatives, create scope for flexible and context-specific action. Figure 4 synthesizes climate-related policies and procedures and evaluates their contributions across the biodiversity-water-food-health nexus. Table S2 provides the scoring rationale, confidence assessments, and actor attribution, while Table S3 reports the full policy matrix.

**Figure 4:**
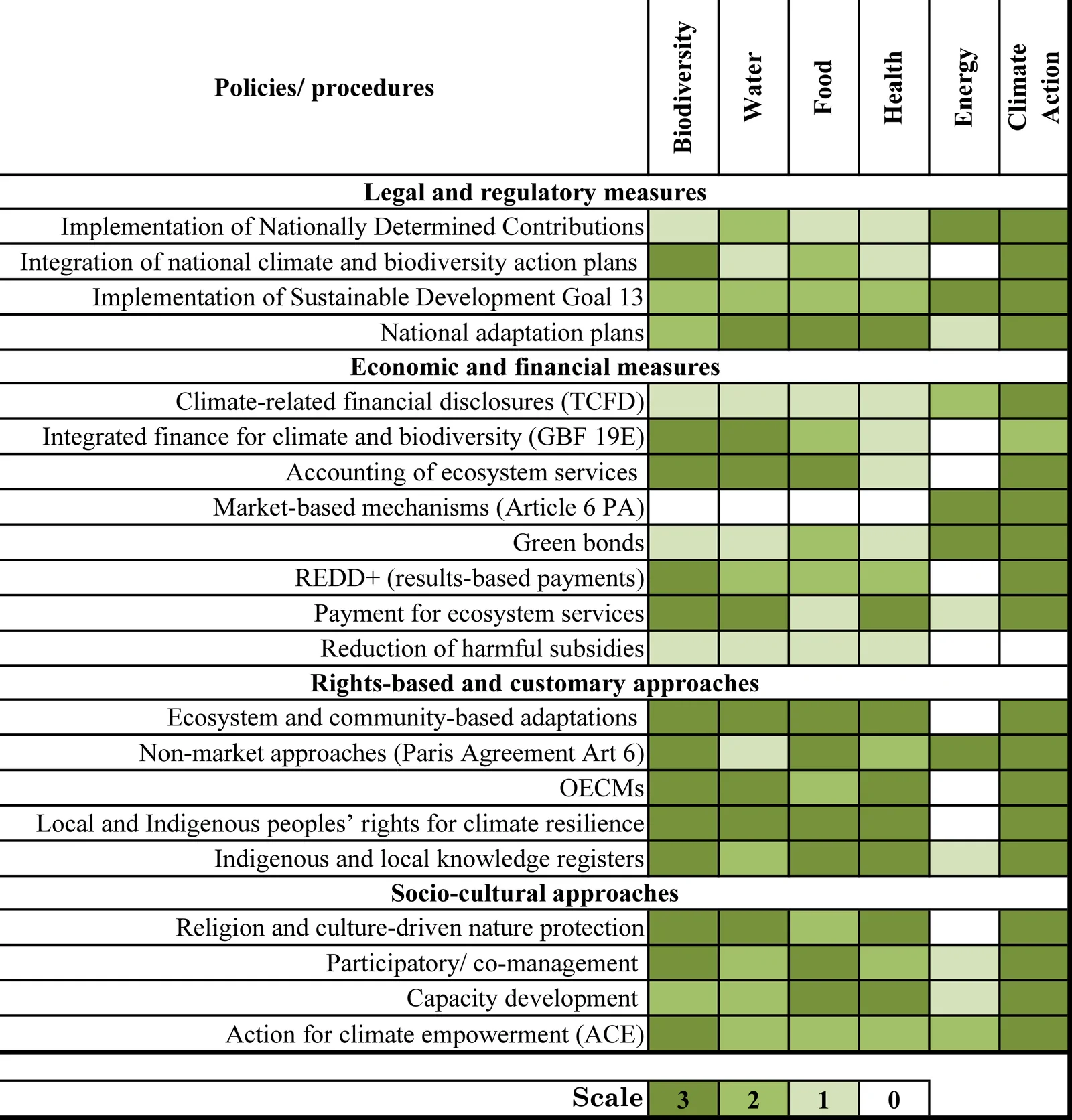
Climate policies and procedures potentially shaping the biodiversity-water-food-health nexus Table S3 provides the detailed policy matrix; Table S9 reports the evaluation criteria.

Despite this diversity, fragmented policy landscapes continue to hinder coordinated implementation. Disjointed mandates across jurisdictions and sectors dilute institutional focus and create overlaps or blind spots. Global frameworks such as the 2030 Agenda for Sustainable Development and the Kunming-Montreal Global Biodiversity Framework promote coherence and integration.^5^ Their translation into national instruments remains uneven, particularly where political priorities diverge or inter-ministerial coordination is weak.

Nationally Determined Contributions (NDCs) under the Paris Agreement are central instruments for aligning mitigation and adaptation efforts with enabling conditions. Recent synthesis evidence shows progression in ambition and scope, with most parties communicating economy-wide targets and a large majority including adaptation components.^57^ Many NDCs reference ecosystems, forests, oceans, resilience, and just transition considerations. Yet integration across biodiversity, water, food, and health systems remains uneven. These interlinkages are often acknowledged but rarely embedded in mandatory safeguards or integrated assessment requirements. While forest and ocean measures are increasingly visible, explicit mechanisms to prevent biodiversity trade-offs in mitigation planning remain limited.^57^^,^^58^ Fragmented institutional mandates risk maladaptive outcomes, particularly where energy transitions proceed without parallel safeguards for ecosystem integrity, tenure security, and distributive equity.

National adaptation plans also provide opportunities for policy integration, yet biodiversity dimensions, including ecological flow requirements in hydropower development, are often neglected.^58^ Only a minority of least developed countries prioritize nutrition within adaptation planning, indicating that the needs of vulnerable populations remain under-represented.^59^

Legal and regulatory instruments, therefore, require a stronger nexus orientation. This includes embedding biodiversity-water-food-health linkages into national and sub-national adaptation planning, integrating procedural safeguards to ensure the participation of Indigenous Peoples and local communities (IPLC), and ensuring cross-ministerial accountability. Such measures can improve coherence and reduce unintended trade-offs.

Economic and financial instruments add another layer of influence. Reducing Emissions from Deforestation and Forest Degradation (REDD+) supports mitigation and biodiversity objectives through forest governance in high-carbon ecosystems.^60^^,^^61^ Ecosystem-based adaptation uses biodiversity-driven strategies to manage climate risks under both the United Nations Framework Convention on Climate Change (UNFCCC) and the convention on biological diversity.^62^ Yet these approaches can generate harm when implemented without social and ecological safeguards. Afforestation with exotic monocultures has displaced native species, strained water resources, and undermined local food systems.^63^^,^^64^

Customary institutions and rights-based approaches help secure tenure, foster accountability, and build legitimacy. For IPLC, whose stewardship underpins biodiversity conservation, these frameworks enable participation in decisions affecting their lands and livelihoods. Despite international recognition, their operationalization in national climate policies and financing mechanisms remains inconsistent and under-resourced.^65^

More broadly, existing policy instruments often fail to address systemic inequalities or institutional exclusion. IPLC engagement remains largely symbolic, while financial mechanisms rarely accommodate plural value systems or build on ILK.^66^ Few instruments define procedural pathways for resolving cross-sectoral trade-offs.

#### Governance frameworks and mechanisms

Beyond individual actors and instruments, broader governance frameworks determine how authority is distributed, how learning occurs, and how trade-offs are managed over time. Climate governance must contend with complex interdependencies and uncertain futures. It therefore requires institutional designs that move beyond sectoral silos and static rules. Polycentric governance and nexus governance offer complementary responses to this complexity.

Polycentric governance distributes authority across multiple, overlapping centers that operate with partial autonomy while remaining interconnected.^54^^,^^55^ This structure supports experimentation, adaptive learning, and context-sensitive responses. Redundancy across centers enhances resilience, while modular autonomy enables innovation without reliance on a single authority.^67^ Variation across governance units can therefore stabilize, rather than weaken, implementation.

Distributed authority alone, however, does not guarantee equitable or coherent outcomes. Without mechanisms to balance power and align mandates, polycentric systems may entrench fragmentation or elite capture.^53^^,^^68^ Transparency, accountability, and procedural safeguards remain essential.

Nexus governance complements polycentricity by addressing interconnections across biodiversity, water, food, health, and climate systems. It is based on five interlinked principles outlined in the IPBES Nexus Assessment^32^: integrative and transdisciplinary framings, inclusive engagement of diverse actors, explicit attention to justice and accountability, cross-scale and cross-sectoral coordination, and adaptive, reflexive governance that learns iteratively from practice.^5^^,^^48^

Together, polycentric and nexus governance combine flexibility with coherence. Polycentric arrangements allow responses to reflect local priorities and conditions. Nexus governance provides the integrative logic required to manage trade-offs and realize co-benefits across systems. Their combination supports climate governance that is adaptive, inclusive, and capable of sustaining outcomes.

#### Global cooperation for finance and technology

This subsection examines why technically viable climate response options often fail to scale equitably or remain durable, especially in low- and middle-income contexts. Access to finance and climate-relevant technologies remains deeply uneven. Many low- and middle-income countries face limited concessional finance, high borrowing costs, and weak institutional capacity.^10^^,^^49^ These barriers restrict investment in long-term resilience and low-carbon development. Despite long-standing commitments under the UNFCCC, adaptation finance remains insufficient, with substantial annual shortfalls.^69^

Transparent and accountable governance of financial flows is critical for low-carbon transitions. Market-based instruments such as carbon pricing and emissions trading can redirect investment incentives.^10^^,^^70^ Blended finance and mechanisms such as REDD+ and blue carbon markets mobilize resources for adaptation while strengthening ecological and economic resilience.^71^^,^^72^^,^^73^ Yet unsustainable debt burdens, risk-averse capital, and entrenched subsidy regimes continue to constrain progress.^49^

Harmful public subsidies for fossil fuels, agriculture, forestry, and fisheries divert resources from renewable- and circular-economy pathways. Reallocating these funds and embedding equity considerations in financial governance are central to a just climate transition.

Access to technology is similarly uneven. Intellectual property regimes, financial barriers, and geopolitical asymmetries limit the diffusion of climate-relevant technologies.^74^^,^^75^ International mechanisms such as the Green Climate Fund and the Climate Investment Platform can help close these gaps by strengthening access and building technical capabilities.

Collaborations between public institutions and private firms play a crucial role in transferring knowledge and innovation across contexts.^49^ Legal tools, including patent waivers and compulsory licensing, have been used to ease restrictions associated with proprietary technologies.^76^^,^^77^ Emerging forms of South-South cooperation offer alternative pathways that prioritize context-specific innovation and local production over dependency on high-income economies.^49^ Approaches that combine regional finance, co-designed research, and local manufacturing are better positioned to democratize access to climate-relevant technologies.

#### Ethical foundations for climate governance

This subsection examines how values, worldviews, and normative commitments shape the legitimacy and durability of climate governance arrangements.

Ethical principles, societal values, and diverse worldviews shape climate decisions that are both socially legitimate and environmentally responsible.^1^^,^^50^ Ethical reasoning influences how climate choices are framed, contested, and justified. Definitions of key sustainability and ethics concepts used in this analysis are provided in Table S4. Frameworks grounded in justice, equity, and sustainability provide shared reference points for governing uncertainty and contestation.^8^^,^^78^ Rights-based approaches and community-led adaptation can open institutional space for more equitable outcomes.^8^^,^^79^

Institutional design reflects underlying values. These values shape what is prioritized, how systems respond to disruption, and how they learn over time.^48^ Worldviews are, therefore, consequential. Anthropocentric models tend to center growth and human utility, often treating ecosystems instrumentally. Ecocentric perspectives emphasize relationality among species and the integrity and resilience of ecosystems.^80^ Governance arrangements that selectively draw on both perspectives may build legitimacy and coherence across competing objectives. Sustainable change depends not only on scientific input but also on the capacity to align evidence, deliberation, and lived experience. Table 3 situates these ethical strands within nexus-based climate governance.

**Table 3:** Ethical foundations in climate action

Response option	Governance priorities	Ethical considerations	Worldview alignment
C01 Increase soil organic carbon	regenerative soils; food sustainability	soil stewardship; long-term resilience	ecocentric agroecology
C02 Sustainable intensification	efficient productivity; land sparing	food security; future equity	pragmatic intensification
C03 Integrated multitrophic aquaculture	circular nutrients; water quality	marine stewardship; ecosystem balance	blue sustainability
C04 Wetland conservation and restoration	water regulation; habitat recovery	ecological justice; critical-habitat protection	biocentric conservation
C05 Offshore wind power	renewables; marine spatial planning	just transition; ocean-use ethics	precautionary pragmatism
C06 Solar photovoltaics on land	distributed energy; access justice	energy equity; land-use ethics	eco-modern pragmatism
C07 Sustainable bioeconomy	biomass efficiency; innovation	circularity; future justice	regenerative systems
C08 Reduce short-lived climate pollutants	pollution control; rapid mitigation	clean-air rights; environmental health	precautionary equity
C09 Multi-hazard early warning systems	Anticipatory action; disaster preparedness	safety; information rights	human security; resilience ethics
C10 Global cooperation for finance and technology	equitable finance; technology transfer	climate justice; historical responsibility	cosmopolitan justice
C11 Agroecology	local resilience; agrobiodiversity	food sovereignty; ecological integrity	biocultural regeneration
C12 Forest-based practices to address climate change	carbon storage; sustainable forestry	conservation ethics; community rights	indigenous knowledge; deep ecology
C13 Restoration of coastal and marine ecosystems for carbon sequestration	ocean stewardship; ecosystem recovery	marine conservation; climate resilience	marine biocentrism; blue economy
C14 Urban nature-based solutions	green infrastructure; environmental justice	climate resilience; social inclusion	urban ecology
C15 Sustainable healthy diets	food-system sustainability; health equity	right to food; ethical consumption	planetary health; food-system transformation

Note: worldview alignment indicates the dominant ethical orientation associated with each response option; categories are interpretive and not mutually exclusive.

The distinction between risk-oriented and rights-oriented ethical framings has institutional consequences. A risk-attuned approach defines sustainability primarily as hazard reduction. It prioritizes rapid mitigation, measurable outputs, and technological deployment. Such strategies can deliver visible climate and health gains, but they may leave tenure, access, and distributional equity insufficiently addressed.

A rights-attuned approach treats equity and participation as design principles. Distributive equity concerns the fair allocation of benefits and burdens. Procedural inclusion refers to meaningful participation in decision-making. Recognition affirms diverse knowledge systems, including ILK. When these elements are embedded in governance, cross-nexus coherence strengthens. Thus, sustainability depends not only on technical effectiveness but also on the ethical orientation that shapes institutional design.

#### Equity, justice, and social inclusion in climate governance

This subsection analyses how equity, inclusion, and historical responsibility condition the effectiveness and legitimacy of climate action.

Climate governance is linked to histories of exclusion that continue to shape exposure and vulnerability. Structural inequalities rooted in colonial exploitation, entrenched social hierarchies, and patterns of environmental degradation influence whose voices are heard and whose interests prevail.^81^^,^^82^ Policies that overlook these asymmetries risk reproducing the injustices they seek to address.

Responding to this challenge requires more than technical adjustment. Institutions must confront how governance arrangements silence certain groups, misdirect public resources, and marginalize alternative knowledge systems.^83^^,^^84^ Equity must therefore be treated as a precondition for legitimacy and effectiveness, not as an ancillary concern.

Three interrelated dimensions of justice are central. Procedural justice concerns inclusive decision-making that brings those facing the greatest climate risks and those historically excluded from political participation into governance processes.^16^^,^^85^ Distributive justice addresses the allocation of financial, technological, and institutional support in ways that reflect differentiated needs and responsibilities.^50^ Recognition justice requires that the worldviews and lived experiences of marginalized communities are affirmed as integral to decision-making.

ESJ extends these concerns to the planetary scale. It treats justice as constitutive of how ecological thresholds are defined and governed, rather than as an external normative overlay.^16^ By holding ecological limits and human dignity within the same analytical frame, ESJ challenges any separation between environmental safety and political responsibility.

Nexus governance, as developed through the IPBES Nexus Assessment, offers a complementary perspective.^5^ Like ESJ, it centers equity, accountability, and pluralism. Its distinctive emphasis lies in operational coherence: identifying synergies and reducing trade-offs across sectors and scales.

The two approaches converge in recognizing the indivisibility of environmental and social goals, the need for knowledge co-production, and the importance of broad-based participation. They diverge in emphasis. ESJ prioritizes redistributive imperatives, historical legacies, and reparative responsibilities, while nexus governance provides institutionally navigable mechanisms for coordination and learning.^5^^,^^16^

Integrating ESJ with nexus governance strengthens climate action by linking ethical evaluation to institutional coordination. ESJ assesses response options against distributive equity, procedural fairness, intergenerational responsibility, and the recognition of diverse knowledge systems. Nexus governance aligns biodiversity, water, food, health, and climate objectives across sectors and scales. Table 4 applies this combined lens. For example, C11 agroecology scores high on both ESJ compatibility and nexus governance synergy because it embeds food sovereignty, ecological integrity, and participatory governance within production systems, reinforcing soil-water-food linkages. Table S5 details the evaluation rationale.

**Table 4:** Evaluating climate action response options through Earth System Justice and nexus governance synergy

Response option	ESJ compatibility	Nexus governance synergy	Current implementation	Scalability	Systemic rationale
C01 Increase soil organic carbon	3	3	embedded in sustainable agriculture programmes	high with policy support	strengthens soil-water-food links; permanence remains critical
C02 Sustainable intensification	2	2	widespread in commercial agriculture	moderate; needs input regulation	productivity gains with rebound and equity risks
C03 Integrated multitrophic aquaculture	3	3	community-driven coastal applications	moderate-high in suitable coastal zones	circular nutrient design supports marine biodiversity-food integration
C04 Wetland conservation and restoration	3	3	biodiversity hotspots and NbS projects	high in suitable hydrological landscapes	restores hydrological regulation and biodiversity with multi-domain benefits
C05 Offshore wind power	2	2	expanding in renewable energy systems	high in resource-rich marine areas	strong mitigation gains; siting governs marine trade-offs
C06 Solar photovoltaics on land	2	2	widely deployed for decentralized power	high with land-use safeguards	decarbonization benefits require land-competition safeguards
C07 Sustainable bioeconomy	3	3	implemented through bio-based industries	moderate-high with biomass governance	circular resource use; sourcing limits shape outcomes
C08 Reduce short-lived climate pollutants	3	3	implemented across urban and rural contexts	high through targeted regulation	rapid climate and health gains with sectoral leverage
C09 Multi-hazard early warning systems	3	3	operational in hazard-prone regions	high with digital and institutional integration	anticipatory risk reduction with indirect biodiversity effects
C10 Global cooperation for finance and technology	4	4	global financing and technology mechanisms	transformative through multilateral reform	system-wide enabling mechanism for other options
C11 Agroecology	4	4	community-based agriculture initiatives	high via extension and policy integration	ecological farming creates food-biodiversity synergies
C12 Forest-based practices to address climate change	4	4	REDD+ and community forestry programmes	high with forest governance safeguards	carbon sequestration integrated with ecosystems and tenure
C13 Restoration of coastal and marine ecosystems for carbon sequestration	4	4	active blue-carbon projects	high in suitable coastal systems	blue carbon supports coastal protection and biodiversity recovery
C14 Urban nature-based solutions	3	3	implemented in metropolitan areas	high within municipal planning systems	urban climate buffering with spatial distribution challenges
C15 Sustainable healthy diets	4	4	growing advocacy and local adoption	high through coordinated food policy	demand-side transformation links food, health, and emissions

Note: ESJ compatibility and nexus governance synergy scores are ordinal expert-synthesis judgments on a 0–4 scale: 0 = none or very low; 1 = limited; 2 = moderate; 3 = strong; 4 = transformative. Scores are not statistical estimates, effect sizes, or direct nexus-impact scores. Direct or near-direct nexus effects are reported separately in Table 1. This table should be read with Supplementary Materials 4, 5, and 10.

#### Indigenous and local knowledge in climate action

This subsection explains how epistemic inclusion shapes the durability and contextual fit of climate responses. ILK systems embody long-standing, place-based understandings of ecological dynamics embedded in cultural and ethical relationships with nature. These systems support adaptive practices across biodiversity, water, food, health, and climate domains and remain central to many communities’ resilience strategies.^73^^,^^86^^,^^87^

Mainstream climate governance has often marginalized ILK by privileging universalized scientific models and technocratic solutions. This epistemic exclusion constrains innovation, weakens legitimacy, and undermines locally rooted capacities. Where integration occurs, it often remains extractive: knowledge is documented, but its political, relational, and governance dimensions remain under-recognized.

Across several response options assessed here, ILK underpins effective adaptation. In forest-based approaches, customary governance and sacred forest conservation align ecological integrity with cultural meaning. Agroecological systems (C11) draw on traditional seed-saving, intercropping, and soil fertility management. In coastal contexts, ILK informs navigation, mangrove restoration, and fisheries governance. In wetland restoration, local knowledge of hydrology and species succession supports ecological recovery and risk reduction.^10^^,^^88^^,^^89^

Weaving ILK with scientific knowledge requires institutional arrangements that recognize knowledge sovereignty and uphold procedural equity. Co-governance arrangements and transdisciplinary collaborations enhance contextual fit and long-term stewardship.^90^ Moving from inclusion to recognition depends on legal and institutional mechanisms that protect collective rights and enable equitable benefit-sharing. Table 5 shows how ILK strengthens multiple response options by reinforcing biocultural diversity and enabling pathways that are both context-sensitive and systemically just.

**Table 5:** Potential contributions of Indigenous and local knowledge to climate action

Response option	Potential contributions of ILK to climate action
C01 Increase soil organic carbon	traditional composting, organic amendments, soil stewardship practices
C02 Sustainable intensification	mixed cropping, indigenous rotation systems, ecological land-use planning
C03 Integrated multitrophic aquaculture	polyculture traditions, nutrient cycling, aquatic ecosystem balance
C04 Wetland conservation and restoration	floodplain governance, local hydrological knowledge, community-led restoration
C05 Offshore wind power	coastal use rights, culturally sensitive siting, marine spatial awareness
C06 Solar photovoltaics on land	sacred landscape mapping, traditional architectural insights, land-use compatibility
C07 Sustainable bioeconomy	low-waste biomass use, traditional harvesting, regenerative resource cycles
C08 Reduce short-lived climate pollutants	efficient cooking practices, fire control traditions, local air quality strategies
C09 Multi-hazard early warning systems	environmental sign interpretation, oral histories, localised risk response
C10 Global cooperation for finance and technology	customary governance integration, community-informed co-design, equitable partnerships
C11 Agroecology	seed-saving, pest management knowledge, ecological farming systems
C12 Forest-based practices to address climate change	sacred groves, rotational access, collective forest stewardship
C13 Restoration of coastal and marine ecosystems for carbon sequestration	mangrove planting knowledge, seasonal tidal insights, community replanting protocols
C14 Urban nature-based solutions	home-garden traditions, rainwater harvesting, thermoregulation techniques
C15 Sustainable healthy diets	local food diversity, seasonal consumption, traditional diets and indigenous food systems

### Illustrative global examples of enabling conditions

Climate change response options: co-benefits, trade-offs, and structural differentiation and enabling climate action: governance, finance, equity, and knowledge identified two central findings: ecosystem-centered and demand-side options tend to generate stronger cross-nexus synergies than infrastructure-centered pathways, and durability depends on governance coherence and justice safeguards as much as on technical design. This section uses five enabling-condition cases and one contested counter-case to examine those mechanisms in context. The focal cases trace enabling conditions across agroecology, indigenous forest governance, blue carbon, urban nature-based solutions, and sustainable healthy diets. The Kachung Forest Project is included as a contested counter-case within C12 to probe the inverse mechanism. Nexus impacts are synthesized in Table S6, while institutional features are detailed in Tables 6 and S7.

**Table 6:** Enabling conditions for five focal climate response options and one contested counter-case

Dimension	Core feature	Andhra Pradesh (Agroecology)^33^^,^^34^^,^^35^^,^^36^^,^^91^	Brazilian Amazon (Indigenous REDD+)^37^^,^^38^^,^^39^^,^^40^^,^^92^^,^^93^	Mikoko Pamoja (blue carbon)^41^^,^^42^^,^^43^	Medellín (urban NbS)^44^^,^^45^^,^^46^^,^^94^^,^^95^^,^^96^	Wayanad (healthy diets & ILK) ^97^^,^^98^^,^^99^^,^^100^	Kachung forest Project^101^^,^^102^^,^^103^^,^^104^^,^^105^^,^^106^^,^^107^
Polycentric governance	multi-level coordination	RySS-SHG-farmer coordination	Amazon fund links IPLCs, NGOs, and state	community-state-donor co-management	municipal leadership with civic-academic actors	layered SHG-CSO-public coordination	CDM-state-corporate links; weak local authority and accountability
	adaptive learning	farmer experiments and field schools	mapping plus satellite oversight	iterative carbon and restoration validation	monitoring and pilot refinement	ILK holder-scientist co-learning	MRV improved; livelihood correction lagged
	equity tensions	adapted agroecology; bureaucratic gaps persist	tenure governance; benefit-sharing risks remain	negotiated livelihood trade-offs	unequal green access	recognition gaps across health, forest, and ILK	local resource use overlooked in project design
Nexus governance	cross-sector framing	soil-water-nutrition-livelihood links	carbon-biodiversity-indigenous rights integration	restoration-livelihood-coast protection	heat-air-biodiversity alignment	food-ecosystem-women’s agency links	carbon/timber dominated within project framing
	participation/accountability	SHG-based marginal farmer inclusion	FPIC-centered decisions	transparent revenue governance	neighborhood co-design	tribal women/healers central	beneficiaries, not co-designers in decisions
	cross-scale reflexivity	agriculture-nutrition-NRM convergence	forest-climate-IPLC alignment	NGO-state-community coordination	inter-agency planning	health-biodiversity-agriculture convergence	global-national coordination exceeded local accountability and grievance correction
Finance/technology	climate finance	public schemes; no carbon credits	donor-backed PES/carbon finance	blue-carbon community services	municipal/donor co-finance	public/CSO funding; little climate finance	CDM finance; contested benefits for communities
	knowledge transfer	seed exchange; agroecology tools	remote sensing plus ILK	satellite/community monitoring	GIS/ecological design	ILK-science co-production	GIS/MRV/silviculture dominated assessment
Ethical foundations	justice values	soil stewardship; dignity	climate justice; indigenous rights	coastal-custodian equity	green equity; well-being	biocultural food ethics	mitigation over livelihoods
	ESJ alignment	agroecosystem procedural/distributive justice	tenure-secure PES justice	blue carbon with development	equitable ecosystem-service access	food/healing justice	weak ESJ alignment across dimensions
Equity and inclusion	recognition/participation	landless and women empowered	partial IPLC authority	community ownership	citizen planning engagement	consensus SHG platforms	customary users weakly recognized in governance
	distributive outcomes	smallholder benefits	community carbon payments	schools and water funded	air-quality/jobs gains	nutrition/income/herbal access	carbon gains; local access losses persisted
	justice mobilization	agroecology movement links	land-rights struggles	blue-justice framing	urban justice mobilisation	diet revival	Carbon-colonialism scrutiny and legitimacy concerns
ILK	science integration	farmer knowledge and agroecology	ILK-satellite-carbon tools	participatory monitoring	community insight in design	ILK in diet/health/agroecology	expert baselines marginalized ILK
	resilience role	seed-soil-cropping knowledge	fire and biodiversity stewardship	mangrove/tidal knowledge	plant/urban adaptation knowledge	climate-resilient diets/healing	restricted access weakened coping
	institutional recognition	informal SHG support	partly formalised; tenure-sensitive	recognized through PES	limited formal recognition	weak legal institutionalization	ILK not embedded

Note: cases are used for qualitative mechanism tracing, not for statistical representativeness.CDM, clean development mechanism; CSO, civil society organization; ESJ, Earth System Justice; FPIC, free, prior and informed consent; GIS, geographic information system; ILK, indigenous and local knowledge; IPLC, Indigenous Peoples and local communities; MRV, monitoring, reporting and verification; NbS, nature-based solutions; NGO, non-governmental organization; NRM, natural resource management; PES, payment for ecosystem services; REDD+, reducing emissions from deforestation and forest degradation; RySS, Rythu Sadhikara Samstha; SHG, self-help group.

Community-managed natural farming in Andhra Pradesh, India, illustrates agroecology (C11) in practice. Reported outcomes include enhanced agrobiodiversity, improved groundwater recharge, increased dietary diversity, and reduced agrochemical exposure.^33^^,^^34^^,^^35^ These outcomes align with the high ESJ compatibility and nexus governance synergy attributed to C11 (Table 4). Agronomic technique alone does not explain the gains. The Rythu Sadhikara Samstha (RySS) platform connected state agencies with self-help groups, farmer networks, and civil society organizations. Authority was decentralized yet coordinated; farmer-led experimentation and field schools enabled adaptive learning.^35^ Redundancy across extension services and community institutions strengthened resilience to market and policy shifts. Women farmers and marginal groups were actively included. Synergies stabilized where distributive equity, recognition of local knowledge, and cross-sectoral coordination converged.^33^^,^^34^^,^^35^^,^^36^^,^^91^

Indigenous-led REDD+ initiatives in the Brazilian Amazon illustrate forest-based practices (C12) under conditions of recognition justice. Documented outcomes include avoided deforestation, habitat protection, strengthened watershed function, and reduced fire-related health burdens.^37^^,^^39^^,^^40^^,^^92^ Food systems benefited through continued access to non-timber forest products. These effects correspond to the strong justice and nexus scores for C12 (Table 4). Tenure security proved decisive, where customary land rights were formally recognized, and indigenous institutions retained authority, mitigation aligned with livelihood security. The Amazon Fund linked IPLC, non-governmental organizations, and state agencies within a multi-level governance architecture. Participatory mapping and satellite monitoring integrated scientific tools with indigenous knowledge. Overlapping institutional linkages enhanced resilience to political change, where communities were treated as beneficiaries rather than rights-holders, conflict, and instability followed. Carbon finance without recognition justice did not secure durable cross-nexus outcomes.^37^^,^^38^^,^^39^^,^^40^^,^^92^^,^^93^

The Mikoko Pamoja initiative in Kenya extends this logic to coastal restoration (C13). Documented effects include mangrove rehabilitation, improved water filtration, salinity buffering, diversification of marine food sources, and livelihood-linked nutrition gains. Carbon revenues finance education and water infrastructure, thereby linking mitigation to social development.^42^^,^^43^ The project operates through community-state-donor co-management. Community oversight governs revenue allocation, and carbon stocks are validated through satellite monitoring and local reporting. Trade-offs between conservation and livelihoods are negotiated in community forums. Blue carbon functions as a nexus intervention when ecological stewardship and accountable finance reinforce one another.^41^^,^^42^^,^^43^ When ESJ principles are not observed, conservation and restoration initiatives can result in the transfer of ownership and control over previously communal (customary collective) territories to private actors, such as investment funds involved in mangrove restoration, as documented in the Sine-Saloum delta of Senegal.^5^^,^^108^^,^^109^ Case-specific responses are therefore required.

Medellín’s urban green corridors in Colombia represent urban nature-based solutions (C14) within a metropolitan context. Reported outcomes include expanded tree cover, improved air quality, reduced heat exposure, and measurable mental and cardiovascular health benefits. These gains required coordination across planning, health, and environmental departments. Municipal leadership enabled integration.^44^^,^^45^^,^^94^ Urban geographic information systems and ecological design frameworks supported monitoring and adaptive refinement (Table 6). Civic bodies, academic institutions, and neighborhood associations participated in co-design. Spatial inequities in access to green spaces persisted, and green gentrification posed risks to vulnerable populations. Ecological and health co-benefits did not automatically secure distributive justice.^44^^,^^45^^,^^46^^,^^94^^,^^95^^,^^96^ Explicit safeguards remained necessary to sustain legitimacy over time.

The revitalization of local diets and health traditions in Wayanad, Kerala state, southwest India, illustrates sustainable healthy diets (C15) as a socio-ecological transition. Reported outcomes include the revival of wild edible species, strengthened food sovereignty, improved dietary quality, and reinforcement of local health practices.^97^^,^^98^^,^^99^^,^^100^ These outcomes correspond to the high ESJ compatibility of C15 (Table 4). Governance operated through layered coordination among local governments, self-help groups, civil society organizations, and tribal institutions. More than 500 training sessions connected indigenous knowledge holders with scientific institutions. This process enabled knowledge co-production, defined as the joint creation of actionable understanding across epistemic communities. Public procurement and local institutions supported market access for diverse crops. Dietary transitions generated system-wide co-benefits when embedded in biocultural diversity and women’s agency rather than framed as individual consumption change.^97^^,^^98^^,^^99^^,^^100^

Across the five enabling-condition cases, strong cross-nexus impacts coincide with polycentric governance, adaptive learning, distributive equity, recognition justice, and locally accountable finance. These features do not operate as add-ons to technical design. They shape who participates, whose knowledge counts, how benefits are distributed, and whether ecological gains persist. The cases, therefore, support the paper’s central inference: variation in cross-nexus performance reflects institutional coherence and justice orientation as well as technical ambition.

The Kachung Forest Project in Uganda serves as the contested counter-case because it holds the response-option category constant while probing the inverse mechanism within forest-based climate action. Official Clean Development Mechanism documents framed the project as an afforestation intervention on degraded lands, with anticipated mitigation and sustainable-development benefits.^101^^,^^102^ Independent studies, however, show that the project’s legitimacy was weakened by contested degradation baselines, restricted access to land, fuelwood, grazing areas, and useful trees, limited recognition of customary resource use, and uneven livelihood benefits.^103^^,^^104^^,^^105^^,^^106^^,^^107^ The case does not invalidate forest-based climate action. It demonstrates that carbon accounting, plantation establishment, and formal certification cannot secure durable co-benefits when procedural inclusion, distributive safeguards, and epistemic recognition remain weak.

## Discussion

This study demonstrates why technically credible climate responses produce uneven outcomes across connected biodiversity, water, food, and health systems. Response-option design establishes the initial ecological and technical possibilities. Ecological context then shapes the direction and magnitude of direct effects. Institutional conditions determine whose interests influence implementation and whose burdens remain visible. Ecosystem-centered and demand-side options often support broader synergies because they work through ecological functions linking several nexus elements. However, implementation of technologies alone cannot sustain those benefits. Governance architecture, distributive arrangements, and epistemic inclusion determine whether synergies endure, fragment or become contested.

Infrastructure- and technology-centered pathways reveal this institutional mediation most clearly. Land-based solar photovoltaics (C06) and offshore wind power (C05) reduce emissions and air-pollution exposure.^10^^,^^11^ Their biodiversity and food-system effects depend on siting, spatial planning, and benefit-sharing. Many reported trade-offs arise from land or marine allocation rather than technological incompatibility. Planning can align mitigation with local development when it respects ecological thresholds, tenure rights, and community consent. Centralized decisions can instead concentrate benefits while dispersing social and ecological costs. Spatial governance therefore shapes the wider nexus performance of these technologies.

The sustainable bioeconomy (C07) shows how resource demand can redirect cross-nexus outcomes. Circular resource use may reduce emissions and support livelihoods.^5^^,^^10^ Yet biomass sourcing, ecological limits and land governance condition its biodiversity and water effects. Forest-based practices (C12) display comparable institutional dependence. Carbon sequestration can reinforce biodiversity and livelihoods where communities retain authority and tenure rights.^38^^,^^40^ Benefits weaken when carbon accounting displaces recognition, participation and enforceable safeguards.^110^ Documented REDD+ and plantation-carbon controversies demonstrate the resulting legitimacy risks.^110^ Quantified carbon gains cannot compensate for insecure rights or inequitable benefit-sharing.

Polycentric governance distributes authority among overlapping centers of decision-making. This arrangement can support experimentation, learning, and context-sensitive adaptation.^54^^,^^55^ However, decentralization without coordination may reproduce sectoral silos and existing power asymmetries.^53^^,^^68^ Nexus governance addresses this weakness by making explicit the interdependencies across biodiversity, water, food, health, and climate.^5^ Polycentric arrangements and nexus governance are therefore complementary rather than interchangeable. Polycentric arrangements create space for adaptation, while nexus governance connects decisions across sectors and scales. Their combination can expose trade-offs, assign responsibility and strengthen accountability. Fragmented authority can weaken technically promising interventions when these integrative functions remain absent.^33^^,^^38^

ESJ treats justice as a constitutive element of Earth system governance.^16^ It connects distributive equity, procedural fairness, recognition of diverse worldviews, and intergenerational responsibility. This perspective explains why procedural inclusion affects more than social acceptance. It shapes access, authority, and the institutional legitimacy required for ecological gains to persist. Agroecology relies on farmer autonomy and collective organization.^35^^,^^36^ Forest-based strategies require secure tenure and equitable benefit-sharing.^37^^,^^38^ Multi-hazard early warning systems (C09) protect lives only when information reaches marginalized populations.^10^^,^^11^ Justice alignment therefore influences both implementation quality and outcome durability.

Knowledge systems provide a further mechanism linking legitimacy with ecological performance. ILK comprises place-based understanding embedded in social relations, cultural practices and ethical obligations toward nature.^86^^,^^87^^,^^111^ Governance can strengthen adaptive capacity when it respects knowledge sovereignty and supports co-governance. Knowledge sovereignty means that knowledge holders retain authority over how their knowledge is interpreted, shared, and applied. Instrumental use of ILK without recognition or decision-making power can erode legitimacy.^39^^,^^41^ Epistemic inclusion therefore requires institutional authority, not merely consultation or information extraction.

Financial and technological arrangements act as structural filters across all response options. Limited concessional finance, high debt burdens, and risk-averse investment restrict implementation in many low- and middle-income settings.^10^^,^^49^ Market-based instruments can mobilize capital, but safeguards determine their distributive and ecological consequences. Carbon pricing may advance mitigation while neglecting biodiversity, tenure, and livelihood protections.^63^ Intellectual property rules and domestic institutional capacity also shape technology access and diffusion.^74^^,^^75^ Global cooperation for finance and technology (C10) therefore conditions feasibility across the option set. Its redistributive promise depends on transparent allocation, enforceable accountability, and locally appropriate implementation.

These findings extend existing response-option assessments in two respects. First, they distinguish direct nexus effects from the mediated effects of governance, finance, tenure, and knowledge systems. Second, they explain durability as an institutional and ethical outcome rather than an automatic consequence of technical effectiveness. Durability refers here to the persistence of cross-nexus benefits over time. This distinction prevents favorable technical scores from being mistaken for stable implementation outcomes.

The comparison also clarifies differences among response-option categories without ranking them intrinsically. Ecosystem-centered and demand-side pathways often connect nexus elements through ecological processes. Infrastructure-centered pathways can generate broad benefits when planning protects ecosystems, rights and livelihoods. System-enabling options work indirectly through coordination, redistribution and institutional reform. These are evidence-supported comparative tendencies, not universal properties of each category. Different literatures emphasize different outcomes, regions and risks, which can influence the apparent pattern.

Finance and technological readiness condition feasibility, but neither determines durability independently. Concessional finance can expand implementation capacity while still reproducing asymmetry through opaque allocation. Technological maturity can strengthen mitigation or adaptation potential while leaving tenure conflicts unresolved. Institutional coherence determines how these resources interact with ecological limits and social rights. The relevant analytical question therefore concerns how capacity, authority, and accountability combine in specific settings.

The structured expert assessment and comparative institutional analysis remain diagnostic rather than predictive. The ordinal scores identify directional patterns within the assessed evidence. They do not estimate causal effects, probabilities, or invariant outcomes. Administrative capacity, legal frameworks, and political economy continue to shape implementation across settings. The analysis therefore supports reasoned comparison while resisting deterministic interpretation.

Technical potential alone cannot explain uneven co-benefits across connected systems. Durable outcomes become more plausible when institutions coordinate across sectors and scales. They also require fair distributions of benefits and burdens, meaningful participation, plural knowledge systems, and accountable long-term finance. Weak conditions can produce displaced costs, fragmented gains, or social contestation despite credible mitigation or adaptation potential. Climate responses should therefore be evaluated through both their direct effects and the institutional conditions governing their persistence. This approach aligns ecological interdependence with rights, responsibilities, and public accountability.

### Limitations of the study

Several limitations constrain the scope of the comparison. The study uses ordinal scoring and qualitative mechanism tracing rather than statistical causal identification. It cannot estimate average treatment effects, probabilities, or universal rankings. Evidence coverage also varies across response options, regions, and institutional settings. Ordinal categories compress contextual differences that may matter for implementation. The literature-based expert assessment requires interpretive judgment despite standardized criteria and author-team calibration. The assessment did not use blinded independent parallel coding, calculate inter-rater reliability, or conduct formal numerical sensitivity analysis. Source counts in Table S11 represent entries cited in this manuscript, not independent studies identified through a systematic review. The focal cases illustrate enabling mechanisms and include one contested counter-case. They do not represent the global distribution of implementation outcomes. Selection boundaries and evidence-focus bias may therefore influence comparisons among response-option types. The findings should be read as evidence-supported comparative tendencies, not deterministic predictions.

Future research should test these comparative tendencies through preregistered systematic review, independent parallel coding, and quantitative sensitivity analysis. Preregistration would specify search, screening, and analytical decisions before evidence synthesis begins. Independent parallel coding would allow reviewers to classify evidence separately before resolving disagreements. Quantitative sensitivity analysis could examine whether alternative scoring assumptions alter the reported comparative patterns. These procedures would strengthen reproducibility without converting context-dependent judgments into deterministic estimates.

## Resource availability

### Lead contact

Further information and requests for resources and reagents should be directed to and will be fulfilled by the lead contact, Pramod K. Singh, email: pramodirma@gmail.com.

### Materials availability

This study did not generate new physical materials, biological samples, software, or experimental resources. It uses published assessment material, peer-reviewed and gray literature, and the supplementary scoring, and case matrices prepared for this manuscript.

### Data and code availability

• All data used in the analysis are reported in the main tables and supplementary materials. Response-option definitions are reported in Table S1. Policy-instrument scoring rationales are reported in Table S2. Key sustainability and ethics terms are defined in Table S4. The rationale for assessing ESJ and nexus governance together is reported in Table S5. Case-based nexus impacts and enabling conditions are reported in Tables S6 and S7. Scoring indicators and criteria are reported in Tables S8–S10. Evidence coverage and long-list exclusions are reported in Tables S11 and S12. Chapter 5 response-option scoring criteria and process are available at https://doi.org/10.5281/zenodo.13931440.
• This study generated no new code.
• Any additional information required to reanalyze the data reported in this paper is available from the lead contact upon request.

## Declaration on AI-assisted technologies in the manuscript preparation process

An enterprise version of Grammarly was used to improve language, grammar, and readability. The authors reviewed and approved all revisions and remain responsible for the final content.

## Acknowledgments

We thank the members of the IPBES Bureau and Multidisciplinary Expert Panel, including those who served on the Nexus assessment management committee and provided guidance throughout the assessment process. We also thank the external reviewers who commented on drafts of Chapter 5.5 during the production of the Nexus Assessment and the chapter review editor, N.H. Ravindranath. The leadership, guidance, and support of the IPBES secretariat have been invaluable throughout the assessment process.

## Author contributions

P.K.S., conceptualization, methodology, formal analysis, response option assessment, writing – original draft, writing – review and editing, and visualization; H.G., conceptualization, response option assessment, referencing, and review & editing; J.D.-J., M.C.T., F.C., H.D., M.L., M.M.V., S.K., A.I., M.H., and X.X., response option assessment, and review & editing; L.T., drawing figures and visualization; S.H.E., S.C.P., D.M.-D., M.H., T.H.R., P.D.M., P.A.H., and T.v.H., review of response-option assessment, review & editing; P.S., methodology, review of response-option assessment, and review & editing. All authors reviewed and approved of the paper.

## Declaration of interests

The authors declare no competing interests.

## STAR★Methods

### Key resources table

**Table:** 

REAGENT or RESOURCE	SOURCE	IDENTIFIER
**Deposited data**		

Deposited dataset	https://doi.org/10.5281/zenodo.13931440	Not applicable

**Other**		

IPBES Nexus Assessment Summary for Policymakers	IPBES et al.^5^	Published assessment and supporting materials
IPBES Nexus Assessment Chapter 5.5	Singh et al.^10^	Published assessment chapter
IPBES Nexus Assessment Chapter 5.6	Ricketts et al.^112^	Published assessment chapter
Climate-resilient development pathways	Schipper et al.^50^	IPCC WGII Sixth Assessment Report, Chapter 18
Response-option pool and synthesis	Smith et al.^11^	Published synthesis used to identify the broader response-option set
Earth System Justice framework	Gupta et al.^16^	Conceptual framework for justice in Earth system governance
Response-option definitions	This study	Table S1
Policy-instrument scoring rationales	This study	Table S2
Climate policies and procedures potentially shaping the biodiversity-water-food-health nexus	This study	Table S3
Definitions of key sustainability and ethics terminology	This study	Table S4
Rationale for evaluating climate response options through Earth System Justice and nexus governance synergy	This study	Table S5
Nexus impacts across five focal cases and one contested counter-case	This study	Table S6
Enabling conditions for five focal response options and one contested counter-case	This study	Table S7
Indicators and scoring framework for response options	This study	Table S8
Criteria for evaluating policies and procedures enabling climate response options	This study	Table S9
Criteria for assessing climate response options for Earth System Justice compatibility and nexus governance synergy	This study	Table S10
Evidence base, source coverage, confidence, and limitations for the 15 climate response options	This study	Table S11
Selection-boundary audit for long-list climate response options	This study	Table S12

### Method details

#### Study design

The study uses structured evidence synthesis and comparative institutional analysis. It examines why technically viable climate response options generate uneven cross-nexus outcomes, and how governance and justice conditions shape whether these outcomes become durable co-benefits or remain fragmented, contested or short-lived. The design is grounded in a bounded assessment evidence base. It does not report a new systematic review, meta-analysis, experiment, predictive model or causal identification design.

Evidence synthesis refers here to a bounded comparison of already assessed evidence, case material and policy-instrument information. Comparative institutional analysis is the examination of how rules, authority relations, participation, finance, and knowledge systems shape implementation. Global assessments and peer-reviewed syntheses support broad patterns of response options. Empirical studies support specific nexus interactions. Policy and project documents support implementation analysis. Focal cases and the contested counter-case support qualitative mechanism tracing rather than prevalence estimation.

The study proceeds in four steps. First, it identifies and classifies climate response options across system transitions. Second, it compares their direct effects across nexus domains. Third, it evaluates policy instruments, ESJ compatibility and nexus governance synergy. Fourth, it uses focal cases to examine how governance, finance, rights, values and knowledge inclusion mediate implementation.

#### Evidence corpus and boundary

The evidence corpus was anchored in the IPBES Nexus Assessment, especially the Chapter 5 response-option assessment and the climate-focused Chapter 5.5 synthesis.^10^^,^^112^ The analysis also used the IPCC WGII system-transition framing,^50^ the broader response-option synthesis,^11^ the Earth System Justice framework,^16^ and case-specific peer-reviewed and gray literature listed in the Supplementary Materials.

The study did not run a new database search. It therefore does not report search strings, database counts, screening-stage exclusions, or a PRISMA flow diagram. This choice reflects the study design. The manuscript builds on an intergovernmental assessment corpus and applies an additional governance-and-justice framework to it. It does not claim to exhaust all climate-response literature. Transparency is provided through the published 69-option assessment, the supplementary scoring criteria, and the evidence rationales reported in the Supplementary Materials.

Sources were included when they met at least one of four criteria. They had to inform the definition, performance, or implementation of one or more selected climate response options; provide evidence on biodiversity, water, food, health, energy, or climate interactions; document policy instruments, governance arrangements, finance, rights, values, or ILK; or support one of the focal case analyses. Sources were not used where they offered only general climate-policy commentary without clear relevance to response-option performance, cross-nexus effects, governance, justice, or case interpretation.

#### Evidence hierarchy and source use

The analysis used different types of evidence for different purposes. Global assessments and peer-reviewed syntheses supported broad response-option patterns. Peer-reviewed empirical studies supported specific nexus interactions and trade-off claims. Policy reports, project documents, and institutional material supported governance, finance, rights, and implementation analysis. Case studies were used for mechanism-tracing, not for estimating prevalence. Expert judgment was used within the structured IPBES-style assessment process, in which the interpretation of evidence required calibration across domains and response options.

#### Selection of climate change response options

The response-option selection followed the system-transition logic used in Chapter 18 of the IPCC WGII Sixth Assessment Report.^50^ That framing identifies five transition arenas relevant to climate-resilient development: land and ocean ecosystems; energy systems; settlement and infrastructure systems; industrial systems; and societal systems. The published long-list assessment of 69 climate response options provided the intervention boundary for the selection.^11^

Fourteen sector-bound response options were selected from the published long-list assessment.^11^ Global cooperation for finance and technology was included as a cross-cutting enabling option, yielding the fifteen-option set analyzed here. This distinction matters because global cooperation for finance and technology does not operate as a sector-bound intervention in the same way as solar photovoltaics, agroecology, or urban nature-based solutions. It shapes feasibility, equity, and implementation capacity across several response options through finance, technology transfer, capacity-building, and institutional cooperation.

Selection combined representational and explanatory criteria. First, the set had to cover the five transition arenas rather than privilege a single sector or technology. Second, each option needed to demonstrate or emerging relevance for climate change mitigation, adaptation, or both. Third, each option needed to have traceable effects across the biodiversity, water, food, health, and climate domains. These cross-domain effects are referred to here as nexus effects.

The selected set deliberately includes different types of climate action. Land and ocean ecosystem transitions are represented by increases in soil organic carbon, sustainable intensification, integrated multitrophic aquaculture, wetland conservation and restoration, agroecology, forest-based practices, and the restoration of coastal and marine ecosystems. Energy transitions are represented by offshore wind power and land-based solar photovoltaics. The sustainable bioeconomy and reductions in short-lived climate pollutants represent industrial transitions. Settlement and infrastructure transitions are represented by urban nature-based solutions. Societal transitions are represented by sustainable healthy diets. Multi-hazard early warning systems and global cooperation for finance and technology affects all. These 15 response options were discussed in various IPBES author meetings across chapters. Table S11 reports the evidence base, source coverage, evidence type, geography, confidence, and limitations for each response option. Table S12 lists long-list options/categories not examined as separate analytical units, explains the reasons for non-selection, identifies potential selection-bias risks, and explains how bias was mitigated.

Non-selected options were not treated as unsuitable or ineffective. They were not examined in detail where they substantially overlapped with selected options, had narrower sectoral mechanisms, had weaker cross-nexus traceability, or were less useful for testing the governance-and-justice explanation developed in this study. This exclusion logic reduces duplication while preserving variation across transition arenas, actors, spatial scales, and governance challenges.

The final set was not selected as a ranked list of preferred interventions. It was selected to examine variation. Some options show strong co-benefit potential. Some show context-dependent outcomes. Others deliver mitigation or adaptation benefits while raising questions about land use, ecological disturbance, distributional equity, or procedural inclusion. This variation allows the analysis to examine why technically credible climate actions produce different outcomes across biodiversity, water, food, health, and climate systems.

The fifteen selected options are defined in Table S1 and classified in below table. Table 1 reports their observed co-benefits and trade-offs. Table 2 identifies key actors and arenas of engagement. The analysis treats the selected options as analytically useful response types rather than policy prescriptions. Their performance depends on context, institutional capacity, tenure arrangements, safeguards, finance, and political economy.

**Table:** Classification of the selected climate change response options

Climate change response option	Land and ocean ecosystems transitions	Energy system transitions	Settlement & infrastructure system transitions	Industrial system transitions	Societal system transitions
C01 Increase soil organic carbon	✓				
C02 Sustainable intensification	✓				
C03 Integrated multitrophic aquaculture	✓				
C04 Wetland conservation and restoration	✓				
C05 Offshore wind power		✓			
C06 Solar photovoltaics on land		✓			
C07 Sustainable bioeconomy		✓		✓	
C08 Reduce short-lived climate pollutants		✓		✓	
C09 Multi-hazard early warning systems	✓	✓	✓	✓	✓
C10 Global cooperation for finance and technology	✓	✓	✓	✓	✓
C11 Agroecology	✓				
C12 Forest-based practices to address climate change	✓				
C13 Restoration of coastal and marine ecosystems for carbon sequestration	✓				
C14 Urban nature-based solutions			✓		
C15 Sustainable healthy diets	✓				✓

#### Response option scoring

The response option scoring drew on the Chapter 5 process of the IPBES Nexus Assessment.^10^^,^^112^ The Chapter 5.5 author team developed scores through literature-based expert assessment. The process used peer-reviewed and gray literature, cross-author deliberation, and internal consistency checks across the Chapter 5 subchapter teams (5.1–5.5). These scores were included in drafts of the chapter made available during IPBES external reviews of the Nexus Assessment. Disagreements were resolved through discussion and calibration against shared definitions.

The scoring was not implemented as independent, blinded parallel coding by a fixed number of raters. It therefore did not generate an inter-rater reliability coefficient. No formal sensitivity analysis was conducted. Reproducibility rests instead on explicit criteria, reported scoring rationales, public assessment procedures, and deliberative calibration across the author team. These limits are reported because the scores are structured expert syntheses rather than statistical estimates.

The scoring framework comprised ten criteria: spatial scale, temporal scale, impact on nexus elements, social acceptability, alignment with political priorities, economic implications, technical feasibility, equity, alignment with international frameworks, and transformative change potential. Table S8 reports the indicators and scoring rules. Table 1 and Figure 2 draw on these criteria.

The scoring distinguished direct effects from mediated effects. Direct effects are expected consequences of implementing a response option for biodiversity, water, food, health, or climate systems. Mediated effects arise through governance design, finance, tenure, procedural inclusion, knowledge systems, institutional capacity, and political economy. The main tables focus on direct or near-direct effects. The case analysis and Discussion examine mediated mechanisms. No mediated governance effect was used to upgrade a direct nexus-impact score unless the assessed evidence linked that mechanism to the relevant response option.

Impacts on biodiversity, water, food, health, and climate were scored using ordinal scales. Biodiversity and water scores account for changes in state, function, quantity, and quality. Food scores account for access to healthy food, production, and environmental performance. Health scores account for disease burden, disability, well-being, and, where relevant, access to healthcare. Climate scores account for mitigation potential and adaptation effect. Equity was assessed through distributional equity, recognition and inclusion, and procedural equity. Technical feasibility drew on Technology Readiness Levels, ranging from basic principles observed to systems proven in operational environments.

Qualitative confidence followed the IPBES four-box model for the communication of confidence approach. Confidence reflects the quantity and quality of evidence and the level of agreement among sources. Four categories were used: well-established, established but incomplete, unresolved, and inconclusive. Where evidence pointed in different directions, the assessment retained mixed or context-dependent classifications rather than forcing a single positive or negative interpretation. The symbols in Table 1 should therefore be read as directional judgements. They do not report universal effects, effect sizes, or invariant outcomes across all regions and time horizons.

#### Policy instrument evaluation

The policy-instrument analysis examined how governance tools enable or constrain climate response options. Twenty-one instruments were assessed by the Chapter 5.5 author team. They were selected because they recur in the IPBES Chapter 5.5 climate-action assessment and represent the principal legal, economic, financial, rights-based, customary, socio-cultural, and participatory mechanisms through which response options are enabled or constrained. They were grouped into four domains: legal and regulatory instruments; economic and financial instruments; rights-based and customary instruments; and socio-cultural and participatory mechanisms. Figure 4 reports the policy instruments and their nexus effects. Table S2 provides scoring rationales. Table S9 reports the evaluation criteria.

Each policy instrument was assessed on a four-point ordinal scale from 0 to 3. A score of 3 denotes full institutionalisation, cross-sectoral operation, clear mandates, implementation evidence, and embedded safeguards. A score of 2 denotes strong articulation but uneven operationalisation. A score of 1 denotes partial or fragmented uptake. A score of 0 denotes siloed operation, weak institutional anchoring, or risks of maladaptation and inequity. The score does not measure universal policy quality. It evaluates how far an instrument supports integrated climate action across the nexus.

#### ESJ and nexus governance assessment

A second evaluative layer assessed each climate response option for ESJ compatibility and nexus governance synergy. ESJ compatibility measures how far an option incorporates distributive equity, procedural fairness, recognition justice, intergenerational responsibility, and ILK. Nexus governance synergy measures how far an option supports coordinated outcomes across biodiversity, water, food, health, and climate systems.

Both criteria were scored on a 0–4 ordinal scale. A score of 4 indicates transformative ESJ compatibility or systemic nexus governance synergy. A score of 3 indicates strong compatibility or high synergy. A score of 2 indicates moderate incorporation. A score of 1 indicates limited operationalisation. A score of 0 indicates justice incompatibility or fragmentary governance. Table 4 reports the results. Table S5 explains the rationale for combining ESJ and nexus governance. Table S10 reports the full scoring criteria.

The ethical foundations in Table 3 and the ILK analysis in Table 5 support this assessment. These tables were used to check whether response options merely report technical benefits or also address values, rights, participation, recognition, accountability, and knowledge pluralism.

#### Case selection and comparison

Five focal cases were selected for deeper institutional interpretation. They correspond to C11–C15 and represent agricultural, forest, coastal, urban, and food-health systems. The cases are Andhra Pradesh’s Community-managed Natural Farming; Indigenous REDD+ initiatives in the Brazilian Amazon; Mikoko Pamoja’s community-led blue carbon project in Kenya; Medellín’s urban green corridors; and the revitalisation of local health traditions and food baskets in Wayanad. These cases were selected because they provide evidence-rich illustrations of enabling conditions and represent different systems transitions. They are not treated as statistically representative. They are also not treated as unqualified successes.

The Kachung Forest Project in Uganda was added as a contested counter-case within C12. It was selected because it had a technical mitigation rationale and a formal Clean Development Mechanism architecture, yet contested baseline assumptions, access restrictions, limited recognition of customary resource use, and uneven benefit-sharing undermined its legitimacy. It also has a comparatively rich literature base among contested forest-carbon cases.

The counter-case reduces successful-case bias. It does not make the case set statistically balanced. It strengthens qualitative mechanism tracing by showing how a technically plausible forest-based response option can lose legitimacy when justice and governance safeguards are weak. It also has a comparatively rich literature base for a contested forest-carbon case.

The cases were used to examine mechanisms, not to estimate prevalence. They show how institutional design and social legitimacy shape implementation. They do not support claims about average effects across all climate response options.

The case comparison used two matrices. Table S6 reports nexus impacts across cases. Table S7 reports enabling conditions, including polycentric governance, adaptive learning, procedural inclusion, tenure and rights, distributive safeguards, knowledge pluralism, financing, monitoring, legitimacy, and durability. Table 6 condenses these case findings for the main text.

#### Treatment of uncertainty and limits of inference

The analysis uses ordinal scoring, comparative interpretation, and qualitative confidence categories. It does not estimate causal effects in a statistical sense. It does not assign probabilities to outcomes. It does not treat modeled or scored results as deterministic real-world trajectories.

Four safeguards were used to reduce overstatement. First, scoring criteria were standardised and reported in the Supplementary Materials. Second, direct effects were separated from mediated governance effects. Third, mixed evidence was retained as mixed rather than converted into a single positive result. Fourth, scores were subjected to established review within the Chapter 5.5 author team and the IPBES process, including cross-chapter deliberations. Where evidence was incomplete, the manuscript uses cautious language and reports confidence limits.

The assessment may reflect differences in how separate literatures study response options. Ecosystem-centred and demand-side options are often examined through the lenses of co-benefits, resilience, livelihoods, and justice. Infrastructure-centred options are often examined through the lenses of risk, siting, spatial footprint, and ecological impact. The analysis, therefore, treats observed patterns as evidence-supported comparative tendencies, not as intrinsic rankings of response-option quality. Table S11 provides an evidence transparency map for the 15 response options. It distinguishes global assessments, peer-reviewed syntheses, empirical studies, policy and gray literature, and case-based evidence, while clarifying that source counts refer only to sources explicitly used in this manuscript and do not represent a systematic review count of the full literature. Table S12 reports the long-list response options and categories from Smith et al.^11^ that were not examined as separate analytical units, explains the reason for non-selection, assesses potential selection-bias risks, and identifies the safeguards used to mitigate those risks.

### Quantification and statistical analysis

The quantitative elements are descriptive and ordinal. The main analytical units are fifteen climate response options, twenty-one policy instruments, five focal cases, and one contested counter-case. The response-option assessment used ten scoring criteria. The policy-instrument assessment used a 0–3 ordinal scale. The ESJ and nexus governance assessment used a 0–4 ordinal scale. Selected nexus-impact dimensions used −3 to +3 scales. All the scales are reported in the Supplementary Materials. Technical feasibility used Technology Readiness Levels from 1 to 9.

No inferential statistics were used. No *p* values, confidence intervals, regressions, meta-analytic weights, machine-learning models, or simulation outputs were generated. No randomisation, blinding, stratification, or sample-size estimation was applicable. The symbols in Table 1 are ordinal summaries of the direction and consistency of the evidence. They are not effect sizes. The symbols in Table 1 and scores in Table 4 are structured expert syntheses. They are not population estimates.
