# Supplementary material for: Enabling climate action: A nexus approach for delivering co-benefits across biodiversity, water, food, and health
Source: iScience. 2026 Oct 5;29(11):117734. doi: 10.1016/j.isci.2026.117734 (PMC13670937; doi:10.1016/j.isci.2026.117734)
Supplement: Supplementary file 1 [file mmc1.pdf]

iScience, Volume *29*

Supplemental information

Enabling climate action: A nexus approach for delivering co-benefits across biodiversity, water, food, and health

Pramod K. Singh, Himangana Gupta, Julio Díaz-José, Pamela D. McElwee, Maria Cristina Tirado, Francesco Cherubini, Helena Duchková, Marcos Llope, Mariana M. Vale, Shabana Khan, Akihiko Ito, Masakazu Hori, Siri H. Eriksen, Lakshmikant Tiwari, Sui Chian Phang, Daniel Mason-D'Croz, Xiyan Xu, Tiff van Huysen, Mario Herrero, Taylor H. Ricketts, Paula A. Harrison, and Pete Smith

List of Supplementary Materials

Table S1: Response options and their description

Table S2: Rationale for scoring climate policies and procedures through a nexus lens

Table S3: Climate policies and procedures potentially shaping the biodiversity-water-food-health nexus

Table S4: Definitions of key sustainability and ethics terminology

Table S5: Rationale for evaluating climate response options through Earth System Justice and nexus governance synergy

Table S6: Nexus impacts across five focal cases and one contested counter-case

Table S7: Enabling conditions for five focal response options and one contested counter-case

Table S8: Indicators and scoring framework for response options

Table S9: Criteria for evaluating policies and procedures enabling climate response options

Table S10: Criteria for assessing climate response options for Earth System Justice compatibility and nexus governance synergy

Table S11: Evidence base, source coverage, confidence, and limitations for the 15 climate response options

Table S12: Selection-boundary audit for long-list climate response options, related to STAR Methods and Tables 1 and 4

General note on the Tables

The Supplementary Materials report the analytical basis for the structured comparative assessment used in the manuscript. They define the response options, policy instruments, scoring criteria, evidence bases, case matrices and selection boundaries. Scores are ordinal, evidence-informed judgements. They do not represent effect sizes, probabilities, model outputs or universal rankings. Case materials support qualitative mechanism tracing, not prevalence estimation. Confidence terms indicate the strength, consistency and agreement of the available evidence. They do not imply that outcomes will remain constant across regions, institutions or time horizons.

Throughout these materials, direct effects refer to the expected consequences of implementing a response option in nexus systems. Mediated effects arise through governance, finance, tenure, inclusion, knowledge systems, institutional capacity, or political economy. Enabling conditions are the institutional and social arrangements that shape implementation. Durability refers to the persistence of cross-nexus outcomes over time. Confidence denotes the assessed strength, consistency, and agreement of the available evidence.

Table S1: Response options and their description

Response options	Description
C01: Increase soil organic carbon	Increasing soil organic carbon in managed lands enhances carbon sequestration, soil health, and biodiversity, while improving water retention and crop yields. It supports food security, climate mitigation, and resilience to drought, erosion, and extreme weather events.
C02: Sustainable intensification	Sustainable intensification improves productivity without expanding farmland, using resource-efficient practices and ecological innovation. It enhances food and nutrition security, reduces greenhouse gas emissions, conserves biodiversity, and strengthens climate resilience while addressing equity and sustainability in agricultural systems.
C03: Integrated multitrophic aquaculture	Integrated Multitrophic Aquaculture co-cultivates fed and extractive aquatic species to recycle nutrients and reduce waste. It provides nutritious food, supports biodiversity, mitigates carbon emissions, and fosters climate resilience in coastal systems with lower environmental impact than conventional aquaculture.
C04: Wetland conservation and restoration	Conserving and restoring wetlands—such as marshes, peatlands, and floodplains—enhances carbon storage, water regulation, and biodiversity. These ecosystems buffer climate extremes, support livelihoods, and offer cost-effective, nature-based solutions for adaptation and mitigation.
C05: Offshore wind power	Offshore wind harnesses kinetic energy from coastal and deep-sea winds to generate renewable electricity. It offers a scalable, low-emission energy source, supporting decarbonisation, energy security, and climate goals while reducing pressure on terrestrial ecosystems.
C06: Solar photovoltaics on land	Land-based solar PV systems convert sunlight into clean electricity, reducing reliance on fossil fuels. They offer a scalable solution for emissions reduction, energy access, and climate mitigation, though land use trade-offs require careful management.
C07: Sustainable bioeconomy	The sustainable bioeconomy utilises biological resources through circular practices—bio-based products, waste valorisation, and water efficiency—to reduce emissions, enhance biodiversity, and support livelihoods, aligning economic development with climate goals and environmental sustainability.
C08: Reduce short-lived climate pollutants	Reducing SLCPs like methane and black carbon mitigates climate change, improves air quality, and safeguards health. Targeted actions in energy, agriculture, and transport yield rapid climate benefits while promoting equity and environmental justice.
C09: Multi-hazard early warning systems	MHEWS integrate climate data to anticipate and communicate hazards like floods, heatwaves, and disease outbreaks. They enable timely responses, protect lives, and strengthen adaptation across food, water, health, and biodiversity systems.
C10: Global cooperation for finance and technology	Equitable global cooperation ensures access to climate finance, innovation, and low-carbon technologies. It supports mitigation, adaptation, and capacity-building, particularly in developing countries, advancing inclusive transitions aligned with climate and development goals.
C11: Agroecology	Agroecology applies ecological principles to farming, combining biodiversity, local knowledge, and sustainable practices. It improves resilience, supports healthy diets, enhances food sovereignty, and reduces reliance on external inputs, addressing both social and environmental dimensions of climate action.
C12: Forest-based practices to address climate change	Forest-based strategies—like afforestation, reforestation, and agroforestry—sequester carbon, conserve biodiversity, regulate water, and support livelihoods. They are essential for mitigation, adaptation, and equitable development while protecting Indigenous rights and forest ecosystems.
C13: Restoration of coastal and marine ecosystems for carbon sequestration	Restoring mangroves, seagrasses, and salt marshes enhances blue carbon storage, protects coastlines, and supports biodiversity. These ecosystems buffer climate impacts and deliver adaptation, mitigation, and livelihood co-benefits through NbS.
C14: Urban nature-based solutions	Urban NbS are green and blue urban infrastructure like green roofs, urban forests, and wetlands, that reduce heat, air pollution, and flood risks. They promote biodiversity, improve public health, and enhance resilience, integrating ecological infrastructure into sustainable urban development.
C15: Sustainable healthy diets	Sustainable healthy diets exert minimal environmental pressure, contribute to lower greenhouse gas emissions, conserve biodiversity, and enhance human health and well-being. They prioritise plant-based foods, incorporate responsibly produced animal-sourced products, and seek to minimise food loss and waste. Such diets are not only nutritionally adequate, accessible, and affordable but are also culturally appropriate and ecologically grounded. By aligning with diverse food systems and local contexts, they support equity, nutritional security, and climate resilience.

Note. These descriptions define the analytical scope of each response option. They do not imply uniform effectiveness across all settings. Outcomes depend on ecological context, governance design, finance, tenure, implementation capacity and social legitimacy.

Table S2: Rationale for scoring climate policies and procedures through a nexus lens

Policies/Procedures	Biodiversity	Water	Food	Health	Energy	Climate Action	Key Actors	Confidence
**Legal and regulatory**								
Implementation of Nationally Determined Contributions	1, Biodiversity integration in NDCs is limited and often indirect, with few countries explicitly prioritising biodiversity co-benefits.	2, NDCs have improved recognition of water-energy linkages, though implementation remains partial.	1, Agricultural mitigation/adaptation appear in some NDCs, but food system transformation is nascent.	1, Health co-benefits are acknowledged but rarely prioritised in NDCs.	3, Energy transition is a central pillar of most NDCs, driving mitigation commitments.	3, Legally anchored economy-wide instrument with high institutional traction.	National governments, non-state actors, local governments, private sector	*Established but incomplete*
Integration of national climate and biodiversity action plans	3, Strong synergistic intent and institutional commitment evident in policy alignment with biodiversity goals.	1, Water components are present but not consistently integrated across climate-biodiversity plans.	2, Moderate integration of agrobiodiversity and food security; implementation is uneven.	1, Limited operational integration of climate-health links; mostly sectoral or project-based.	0, Energy is not a focus area in biodiversity-climate synergies; no institutional mechanisms included.	3, Provides strategic convergence across multiple goals; alignment at national level is strengthening.	National, local and city governments	*Well established*
Implementation of Sustainable Development Goal 13	2, SDG 13 recognises biodiversity links, though operationalisation is moderate and non-binding.	2, Water resilience is directly referenced in SDG 13 targets and indicators.	2, SDG 13 mainstreams food resilience concepts but remains general in scope.	2, SDG 13 includes health resilience and awareness but lacks enforcement.	3, SDG 13 reinforces renewable energy and efficiency through its linkages with SDG 7.	3, Broad climate governance tool; widely adopted and reported under UNFCCC processes.	National governments, non-state actors, private sector	*Established but incomplete*
National adaptation plans	2, Biodiversity considerations are incorporated in sectoral adaptation but not yet systemic.	3, Water sector is a key focus; adaptation plans commonly include water security actions.	3, Food systems are core to national adaptation discussions; application is expanding.	3, Strong attention to health vulnerabilities in adaptation programming and community-level plans.	1, Energy is addressed peripherally in sectoral adaptation; mostly through efficiency or decentralised systems.	3, Central planning tool for national adaptation with moderate implementation challenges.	National, local and city governments, citizens, IPLCs	*Established but incomplete*
**Economic and financial**								
Climate-related financial disclosures (TCFD)	1, Limited integration of biodiversity in financial disclosures; nature risks not fully mainstreamed.	1, Water-related risks are often underrepresented; sector-specific disclosures are evolving.	1, Food system risks are indirectly addressed; little sectoral guidance in current frameworks.	1, Health implications of climate risks are not explicitly assessed under TCFD.	2, Energy transition risks and opportunities are central to TCFD frameworks.	3, Strong alignment with climate mitigation and corporate transparency goals.	Businesses, state and local governments	*Established but incomplete*
Integrated finance for climate and biodiversity (GBF 19E)	3, Clearly allocates funding to biodiversity protection; multi-criteria	3, Water-related financing is prioritised for natural	2, Agricultural co-benefits present but not systematically monitored or financed at scale.	1, Health outcomes are not yet clearly linked to biodiversity-climate	0, No significant emphasis on energy transitions in GBF-linked finance.	2, Aligned with co-benefits framing but lacks economy-wide coverage or safeguards.	Financing institutions, national government	*Well established*

Policies/Procedures	Biodiversity	Water	Food	Health	Energy	Climate Action	Key Actors	Confidence
	impact investments advancing.	infrastructure and conservation finance.		financing frameworks.				
Accounting of ecosystem services	3, High recognition and valuation of biodiversity within ecosystem service frameworks.	3, Water provisioning and regulation services are central to valuation exercises.	3, Strong emphasis on food provisioning, agrobiodiversity, and pollination services.	1, Health co-benefits are under-theorised and inconsistently accounted for.	0, Energy-related services are not central to ecosystem service metrics.	3, Widely adopted tool for internalising nature into climate governance.	Local communities, practitioners, civil society, government	*Well established*
Market-based mechanisms (Article 6 PA)	0, Biodiversity risks from carbon markets (e.g., monocultures) often unaddressed.	0, Water impacts are not explicitly assessed or safeguarded in many offset schemes.	0, Food security trade-offs from land-use changes linked to offsets are common.	0, Health co-benefits are not integrated into carbon market instruments.	3, Strong driver of low-carbon transitions through carbon pricing and market signals.	3, Highly aligned with mitigation goals but may generate trade-offs without safeguards.	Private sector, development banks, resource users	*Well established*
Green bonds	1, Some projects target biodiversity but lack robust ecological safeguards or monitoring.	1, Water infrastructure and nature-based solutions increasingly funded but fragmented.	2, Food-related investments (e.g., sustainable agriculture) are growing but still niche.	1, Limited projects directly target health, though co-benefits may accrue.	3, Strong alignment with renewable energy and clean infrastructure investments.	3, Mainstream financial vehicle for climate solutions with varied sectoral depth.	Private sector, development banks, resource users	*Well established*
REDD+ (results-based payments)	3, Directly incentivises biodiversity conservation through forest preservation.	2, Positive water impacts via watershed protection; outcomes depend on location.	2, Co-benefits for food security exist but are context dependent and variable.	2, Health benefits from improved ecosystems are indirect but relevant.	0, No energy focus or integration in REDD+ frameworks.	3, Widely recognised carbon mitigation strategy with measurable outcomes.	National governments, resource users, IPLCs, private sector	*Unresolved*
Payment for ecosystem services	3, Encourages biodiversity conservation via economic incentives and land stewardship.	3, Water purification and supply services often prioritised in PES schemes.	1, Food impacts are not core to most PES design; agricultural links underdeveloped.	3, Clean water and ecosystem stability generate substantial health co-benefits.	1, Energy is rarely a direct component, though firewood/biomass reductions may occur.	3, Innovative tool aligning conservation with livelihoods and mitigation.	Local communities, sub-national and national governments, NGOs	*Unresolved*
Reduction of harmful subsidies	1, Some impact on biodiversity when redirecting subsidies from extractive sectors.	1, Water-intensive subsidies (e.g., for irrigation or fossil-fuel-based power) are often targeted.	1, Food system incentives are gradually shifting, but reform is partial and uneven.	1, Health implications (e.g., from pollution subsidies) recognised but not primary rationale.	0, Reforms do not always align with energy justice; fossil subsidies remain politically embedded.	0, Reform processes are ongoing but face strong institutional resistance and trade-offs.	National government, international trade entities	*Unresolved*
**Rights-based and customary**								
Ecosystem and community-based adaptations (EbA and CbA)	3, Biodiversity conservation is central; promotes ecosystem integrity through community-led stewardship.	3, EbA enhances water retention, recharge, and watershed protection based on local knowledge.	3, Food system resilience strengthened through ecosystem approaches and agroecology.	3, Health is improved via nature-based adaptation, clean water access, and reduced climate vulnerability.	0, Energy dimension is not targeted explicitly within EbA/CbA frameworks.	3, Strongly aligned with climate adaptation goals and community empowerment.	National and local governments, IPLCs, women, youth	*Well established*
Non-market approaches (Paris Agreement Article 6)	3, Encourages recognition of non-monetary values of ecosystems and rights-based biodiversity strategies.	1, Water systems acknowledged, but implementation is minimal and varies by national interest.	3, Supports sustainable practices with community-based food systems.	2, Health dimensions are recognised in principle but uneven in integration.	3, Promotes decentralised, socially inclusive energy access indirectly.	3, Important counterbalance to market mechanisms with strong equity orientation.	National and local governments, civil society, private sector	*Well established but incomplete*
Other effective area-based conservation measures (OECMs)	3, Explicitly prioritises biodiversity protection across terrestrial and marine ecosystems.	3, Strong water security co-benefits from landscape	2, Food co-benefits through wild food sources, agroecology, and access protection.	3, Enhances resilience to climate-health risks via ecosystem services.	0, Energy aspects are absent; no provisions for low-carbon transitions.	3, Effective tool for climate resilience and nature-based mitigation.	MEAs, national and sub-national governments, civil society, IPLCs	*Unresolved*

Policies/Procedures	Biodiversity	Water	Food	Health	Energy	Climate Action	Key Actors	Confidence
		restoration and wetland protection.						
Local governance/ local and Indigenous peoples rights for climate resilience	3, Customary tenure and biodiversity management are central; Indigenous practices conserved.	3, Watershed co-management strengthens water governance rooted in traditional knowledge.	3, Food security is enhanced through sovereign, culturally-appropriate food systems.	3, Health resilience built via clean ecosystems, nutrition, and healing traditions.	0, Energy governance mostly absent; limited role in systemic energy transition.	3, Foundational for climate justice and effective adaptation.	National and local governments, IPLCs, women, youth	*Well established*
Indigenous and local knowledge registers	3, Helps preserve biodiversity-related traditional knowledge and practices.	2, Records water-use traditions, though access remains limited in institutional uptake.	3, Strengthens food security by codifying ILK around seed systems and agroecology.	3, Supports culturally-embedded health knowledge and resilience to shocks.	1, Some ILK around biomass and fuel use, but not formalised for broader energy governance.	3, Enhances knowledge justice, essential for equitable and localised climate action.	IPLCs, national and local governments	*Well established*
**Socio-cultural approaches**								
Religion and culture-driven nature protection	3, Sacred groves and spiritually protected areas preserve key biodiversity and native species.	3, Traditional values support watershed protection and seasonal water rituals.	2, Cultural practices support subsistence agriculture and preservation of traditional food systems.	3, Sacred landscapes improve mental and community health and buffer environmental shocks.	0, No explicit role in renewable or energy policy; indirect benefits only.	3, Deeply rooted in communities and effective for sustaining conservation values over generations.	IPLC, local governments	*Well established*
Participatory/ co-management	3, Collaborative governance strengthens community-based conservation and species management.	2, Water governance structures benefit from local knowledge but need formal authority.	3, Enhances adaptive capacity of local food systems and ensures food access.	2, Health co-benefits through improved sanitation and water-health linkages.	1, Some local co-management of off-grid solar or biomass observed, but not systemic.	3, Strong climate alignment via empowerment and adaptive learning.	Community/IPLCs, resource users, civil society, local governments	*Well established*
Capacity development	2, Biodiversity awareness and stewardship built through training and extension services.	2, Water literacy and conservation practices supported through local capacity efforts.	3, Supports diversification of food systems through livelihood training and input support.	3, Enhances health resilience through women's collectives, nutrition programmes, and training.	1, Energy efficiency or access integrated in some programmes, though not consistently.	3, Empowers grassroots actors to implement and scale climate adaptation.	Civil society, local governments, IPLCs, international organisations	*Well established*
Education, and awareness/Action for climate empowerment (ACE)	3, Promotes understanding of biodiversity and ecosystem linkages with climate risks.	2, Encourages behavioural change in water use through educational campaigns.	2, Food literacy and awareness built around sustainable diets and climate-smart practices.	2, Strengthens public health awareness on climate-health risks through school and media.	2, Energy-saving behaviours and renewable adoption promoted, though unevenly applied.	3, Strategic pillar of UNFCCC to enhance civic capacity for climate action.	Civil society, local governments, international organisations	*Well established*

Table S3: Climate policies and procedures potentially shaping the biodiversity-water-food-health nexus

Policies/ procedures	What does it entail?	Biodiversity	Water	Food	Health	Energy	Climate Action	Key actors	References	Confidence
**Legal and regulatory measures**										
Implementation of Nationally Determined Contributions	Achieving economy-wide targets under country submissions	1	2	1	1	3	3	National governments, non-state actors, private sector	[1,2]	*Established but incomplete*
Integration of national climate and biodiversity action plans	Synergising policies at national and local levels for effective and efficient implementation	3	1	2	1	0	3	National, local and city governments	[3–8]	*Well established*
Implementation of Sustainable Development Goal 13	Resilience, adaptive capacity, mainstreaming into national policies, awareness	2	2	2	2	3	3	National governments, non-state actors, private sector	[9,10]	*Established but incomplete*
National adaptation plans	Focusing on adaptation sectors like forestry, water, food, health	2	3	3	3	1	3	National, local and city governments, citizens, IPLCs	[11–13]	*Established but incomplete*
**Economic and financial measures**										
Climate-related financial disclosures (TCFD)	Disclosure framework for businesses and financial institutions to assess and disclose climate-related risks and opportunities.	1	1	1	1	2	3	Businesses, state and local governments	[14]	*Established but incomplete*
Integrated finance for climate and biodiversity (GBF 19E)	Streamlining finance to achieve multiple objectives	3	3	2	1	0	2	Financing institutions, national government	[15,16]	*Well established*
Accounting of ecosystem services	Comprehensive evaluation of ecosystem services for climate, food, health, and biodiversity	3	3	3	1	0	3	Local communities, practitioners, civil society, government	[17–19]	*Well established*

Policies/ procedures	What does it entail?	Biodiversity	Water	Food	Health	Energy	Climate Action	Key actors	References	Confidence
Market-based mechanisms (Article 6 PA)	Carbon pricing and mainstreaming in all sectors	0	0	0	0	3	3	Private sector, development banks, resource users	[20,21]	*Well established*
Green bonds	Finance climate-aligned projects through labelled debt instruments, subject to disclosure, verification and safeguard quality	1	1	2	1	3	3	Private sector, development banks, resource users	[22,23]	*Well established*
REDD+ (results-based payments)	Real, measurable, long term and additional carbon sequestration/stabilisation	3	2	2	2	0	3	National governments, resource users, IPLCs, private sector	[24–26]	*Unresolved*
Payment for ecosystem services	Reducing emissions from deforestation, eco-tourism, protected areas, clean water	3	3	1	3	1	3	Local communities, sub-national and national governments, NGOs	[27,28]	*Unresolved*
Reduction of harmful subsidies	Reducing subsidies that result in high trade-offs	1	1	1	1	0	0	National government, international trade entities	[29]	*Unresolved*
**Rights-based and customary approaches**										
Ecosystem and community-based adaptations (EbA and CbA)	Empowering grassroots actors, including IPLCs, women and youth into decision-making	3	3	3	3	0	3	National and local governments, IPLCs, women, youth	[30–32]	*Well established*
Non-market approaches (Paris Agreement Article 6)	Mainstreaming sustainability at an economy-wide scale.	3	1	3	2	3	3	National and local governments, civil society, private sector	[18,20]	*Well established but incomplete*
Other effective area-based conservation measures (OECMs)	Conserving and restoring lands, marine and coastal ecosystems to build climate resilience	3	3	2	3	0	3	MEAs, national and sub-national governments, civil society, IPLC	[33–36]	*Unresolved*
Local governance and Indigenous Peoples' rights for climate resilience	Empowering grassroot actors, including IPLCs, women and youth into decision-making	3	3	3	3	0	3	National and local governments, IPLCs, women, youth	[37–40]	*Well established*
Indigenous and local knowledge registers	Documenting ILK, creating data platforms, and securing access and benefit sharing to build climate resilience	3	2	3	3	1	3	IPLCs, national and local governments	[39,41–43]	*Well established*
**Socio-cultural approaches**										

Policies/ procedures	What does it entail?	Biodiversity	Water	Food	Health	Energy	Climate Action	Key actors	References	Confidence
Religion and culture-driven nature protection	Sacred landscapes, biocultural diversity and conservation linked to culture and religion. Protects carbon stock	3	3	2	3	0	3	IPLCs, local governments	[44–48]	*Well established*
Participatory/ co-management	In addition to implementation and monitoring, community is engaged in planning the intervention, acting as a decision-maker	3	2	3	2	1	3	Community/IPLCs, resource users, civil society, local governments	[49–51]	*Well established*
Capacity development	Developing capacities of the community through economic and social support systems	2	2	3	3	1	3	Civil society, local governments, IPLCs, international organisations	[52,53]	*Well established*
Education, and awareness/Action for climate empowerment (ACE)	Creating awareness on resource use through education and training	3	2	2	2	2	3	Civil society, local governments, international organisations		*Well established*

Note. Energy is included because several climate response options act through energy systems. It is not treated as a primary biodiversity-water-food-health outcome domain in the manuscript.

Scores indicate structured, evidence-informed judgements. They compare the direction, strength and consistency of available evidence across response options or policy instruments. They do not imply deterministic effects, universal transferability or direct causal proof. Where evidence is mixed, context-dependent or incomplete, the tables retain that uncertainty rather than converting it into a single definitive claim.

Table S4: Definitions of key sustainability and ethics terminology

Terminology	Definition	Reference
Ecocentric Ethics	Assigns intrinsic value to ecosystems and all life forms beyond human utility. Supports governance prioritising ecological integrity and intergenerational equity.	[54,55]
Anthropocentric Pragmatism	Human-centred ethics evaluated through practical environmental benefits. Balances ecological goals with socio-political feasibility.	[56]
Blue Sustainability	Sustainable use of ocean resources for livelihoods and growth while ensuring marine ecosystem health and justice for coastal communities.	[57–59]
Biocentric Values	Values that attribute inherent worth to all living beings, independent of human utility.	[60,61]
Technological Pragmatism	Evaluates technologies based on their contextual outcomes and societal implications rather than ideological orientations.	[62,63]
Precautionary Principle	Advocates preventive action in the face of scientific uncertainty to avoid potential environmental harm.	[64,65]
Precautionary Ocean Stewardship	Applies the precautionary principle in ocean governance, prioritising ecosystem protection amidst uncertainty and equity concerns.	[58,66]
Pragmatic Anthropocentrism	Recognises human interests as central while enabling environmental ethics that integrate long-term ecological outcomes.	[56]
Ecomodernism	Promotes decoupling development from environmental degradation via technological innovation; critiqued for techno-fixes and social inequity.	[67,68]
Systems Thinking	A holistic analytical framework to understand and act on interlinkages within complex socio-ecological systems.	[65,69]
Regenerative Design	Focuses on restoring and revitalising ecological and social systems through place-based design and reciprocal relationships.	[70–72]
Cosmopolitan Ethics	Ethical orientation that extends responsibilities globally, beyond national and cultural boundaries.	[73]
Biocultural Diversity	Interconnection of biological, cultural, and linguistic diversity within complex socio-ecological systems.	[65,74,75]
Regenerative Systems	Systems that replenish ecological, material, and energy cycles, enhancing resilience and supporting circular economies.	[74,76]
Deep Ecology	Environmental philosophy asserting the intrinsic worth of all beings and calling for a radical rethinking of human-nature relationships.	[77]
Marine Biocentrism	Ethical stance granting intrinsic value to marine ecosystems, advancing their protection regardless of direct human benefit.	[66]
Blue Economy	Marine-based development strategy aiming for sustainability; critiqued for growth-centric and extractive tendencies unless equity-centred.	[57,78]
Planetary Health	The health of human civilisation and natural systems on which it depends. Emphasises regenerative processes and ethical responsibility in development.	[79]

Note. The terms in this table support the ethical and conceptual vocabulary used in the manuscript. Core terms inform the analysis of climate governance, justice, values, knowledge systems and human-nature relations. Contextual terms clarify adjacent debates where they shape the interpretation of response options.

Table S5: Rationale for evaluating climate response options through Earth System Justice and nexus governance synergy

Response Option	ESJ Compatibility (0–4)	Nexus Governance Synergy (0–4)
C01: Increase soil organic carbon	3 – Strong: Integrates procedural equity and distributive co-benefits for smallholders and resource-poor farmers.	3 – High: Enhances soil–climate–food interlinkages; embedded in regenerative agriculture programmes.
C02: Sustainable intensification	2 – Moderate: Focused on productivity gains; equity outcomes remain uneven across scale and context.	2 – Moderate: Some cross-sectoral alignment, but sectoral silos in water-energy-food remain.
C03: Integrated multitrophic aquaculture	3 – Strong: Respects socio-ecological interdependencies and promotes community-led stewardship.	3 – High: Balances marine biodiversity, food security, and climate resilience through system integration.
C04: Wetland conservation and restoration	3 – Strong: Procedural justice frameworks inclusive of IPLCs; distributional co-benefits observed.	3 – High: Functions at the water–biodiversity–climate interface; increasing adoption in NbS policies.
C05: Offshore wind power	2 – Moderate: Justice safeguards often underdeveloped; distributive benefits skewed in favour of investors.	2 – Moderate: Nexus integration limited by ecological trade-offs and land–sea use conflicts.
C06: Solar photovoltaics on land	2 – Moderate: Energy access equity constrained by land use conflicts and socio-spatial inequalities.	2 – Moderate: Delivers climate and energy benefits but weak on biodiversity and health linkages.
C07: Sustainable bioeconomy	3 – Strong: Aligns well with inclusive livelihoods and local value chains; promotes circularity.	3 – High: Mobilises synergies across climate, food, energy, and land systems.
C08: Reduce short-lived climate pollutants	3 – Strong: Delivers co-benefits across vulnerable populations; enhances procedural justice.	3 – High: Cross-sectoral impact on climate, air quality, and health recognised in governance frameworks.
C09: Multi-hazard early warning systems	3 – Strong: Promotes anticipatory adaptation and inclusivity in climate risk governance.	3 – High: Interfaces strongly with disaster, climate, food, water, energy and health governance systems.
C10: Global cooperation for finance and technology	4 – Transformative: Redistributes capabilities and power; centres justice in institutional arrangements.	4 – Systemic: Enables vertical and horizontal coherence; foundational to climate–development synergies.
C11: Agroecology	4 – Transformative: Grounds justice in Indigenous and local knowledge systems; supports autonomy and dignity.	4 – Systemic: Integrates land, biodiversity, food, and climate through reflexive, community-based governance.
C12: Forest-based practices to address climate change	4 – Transformative: Empowers IPLCs and affirms territorial rights; intergenerational equity is central.	4 – Systemic: Harmonises biodiversity conservation and carbon storage; polycentric governance models emerging.
C13: Restoration of coastal and marine ecosystems	4 – Transformative: Elevates place-based ethics and livelihood equity; fosters interspecies reciprocity.	4 – Systemic: Strong biodiversity–climate–livelihood linkages institutionalised in blue carbon strategies.
C14: Urban nature-based solutions	3 – Strong: Procedural inclusion generally high; equity varies with governance contexts.	3 – High: Synergistic with climate adaptation, urban health, and biodiversity co-benefits.
C15: Sustainable healthy diets	4 – Transformative: Builds food justice, health equity, and cultural resilience; benefits intergenerational wellbeing.	3 – High: Intersects agriculture, biodiversity, and public health; catalyses policy convergence.

Note. Scores assess the compatibility and potential of each response option under governance designs that meet the stated criteria. They do not imply that every implementation of the option achieves the same level of justice or nexus integration. Context-specific safeguards, tenure arrangements, financing structures and institutional accountability remain decisive.

Scores indicate structured, evidence-informed judgements. They compare the direction, strength and consistency of available evidence across response options or policy instruments. They do not imply deterministic effects, universal transferability or direct causal proof. Where evidence is mixed, context-dependent or incomplete, the tables retain that uncertainty rather than converting it into a single definitive claim.

Table S6: Nexus impacts across five focal cases and one contested counter-case

Dimension	Key Element	Andhra Pradesh's Community-managed Natural Farming [80–84]	Indigenous REDD+ initiatives in the Brazilian Amazon [85–90]	Mikoko Pamoja's community-led blue carbon project, Kenya [91–93]	Medellín's urban green corridors [94–99]	Revitalisation of local health traditions and food baskets in Wayanad [100–103]	Kachung Forest Project, Uganda [104–110]
Case Representing response option		C11 Agroecology	C12 Forest-based practices to address climate change	C13 Restoration of coastal and marine ecosystems for carbon sequestration	C14 Urban nature-based solutions	C15 Sustainable healthy diets	C12 Forest-based practices; contested industrial afforestation/CDM counter-case.
Impacts on biodiversity	Enhancement of species richness, genetic diversity, and ecological resilience.	Diversified seed use, natural inputs, and ecosystem-based farming enhance agrobiodiversity.	Avoids deforestation; protects critical habitats; strengthens IPLC conservation rights.	Rehabilitates mangrove habitats, increases habitat complexity and species richness.	Increases tree and plant cover, enriches urban biodiversity corridors with native species.	Revives herbal plants, wild edible species, and cultivars integral to biocultural diversity.	Carbon-accounted tree cover increased, but useful-tree diversity and savannah–woodland functions narrowed.
Impacts on water systems	Hydrological regulation, water purification, and retention capacity.	Enhances groundwater recharge and local water retention through mulch, organic soil cover.	Protects riverine flows, avoids watershed degradation through IPLC forest stewardship.	Improves filtration, buffers salinity; supports rice field reclamation and yields.	Enhances blue spaces and water purification in city environments.	Some evidence of enhanced moisture regulation through home gardens	Water-source concerns persisted; plantation design did not resolve wider access and hydrological risks.
Impacts on food systems	Food security, dietary diversity, and sustainable sourcing.	Promotes millet and diverse crop systems, improves local food autonomy.	Supports wild food and non-timber forest products in community-managed areas.	Diversifies marine and estuarine sources; increases seafood and rice yields.	Food-system effects are mediated and not central to the intervention.	Strengthens food sovereignty through revived local diets and medicinal plants.	Restricted farmland, grazing, fuelwood, and useful-tree access weakened livelihood and food security.
Impacts on health	Public health improvements through nutrition, pollution reduction, and wellbeing.	Reduces agrochemical exposure; improves nutrition security via dietary diversity.	Reduces health burden of forest fires and vector-borne disease via intact ecosystems.	Improves dietary quality through seafood; supports livelihoods linked to nutrition.	Reduces air pollution, heat stress; enhances urban mental and cardiovascular health.	Revives immunity-enhancing foods, herbal remedies, local health traditions.	Health effects were mediated through food stress, fuelwood burdens, poverty, and livelihood insecurity.

Note: Case entries identify reported or documented pathways. They support qualitative mechanism tracing rather than prevalence estimation. They do not imply comparable magnitude across cases or average effects across all implementations of a response option.

Table S7: Enabling conditions for five focal response options and one contested counter-case

Dimension	Key Element	Andhra Pradesh CMNF[27–31]	Brazilian Amazon Indigenous REDD+[32–37]	Mikoko Pamoja[38–40]	Medellín's urban green corridors [41–46]	Wayanad food-health traditions[47–50]	Kachung Forest Project [104–110]
Polycentric Governance	Decentralised multi-level governance	Implemented through RySS, engaging multiple governance levels and CBOs.	Operates through Amazon Fund with coordination across IPLCs, NGOs, and state agencies.	Jointly managed by community organisations, national agencies, and international partners.	Led by municipal government with participation from local and global actors.	Multi-level model involving National/Local Governments, SHGs, and CSOs in layered governance.	CDM–state–corporate coordination was strong; community authority remained weak.
	Adaptive learning and experimentation	Farmer-led experiments facilitated by CSOs and field schools.	Satellite monitoring and participatory mapping shaped through IPLC involvement.	Adapts through feedback loops; now includes seagrass conservation.	Adaptive urban greening refined through continuous monitoring and feedback.	500+ training classes delivered by ILK holders and scientific institutions.	Technical monitoring evolved; access, tenure, and livelihood concerns remained weakly addressed.
	Institutional redundancy and resilience	Redundant support from SHGs, extension staff, farmer networks builds resilience.	Overlapping roles among CSOs, state agencies, IPLC associations buffer external shocks.	Redundancy in community structures, NGOs, and certification ensures continuity.	Multiple agencies and community actors provide institutional backup for longevity.	Redundancy from SHGs, CSOs, PRI institutions, and Ayurvedic linkages strengthens local systems.	Formal institutions existed, but redundancy did not protect affected resource users.
	Diverse actor participation	Participation spans RySS, NGOs, farmer collectives, and local government.	Involves IPLCs, NGOs, donors, government and private actors in shared roles.	Broad engagement across local villages, NGOs, scientific partners, and donors.	Civic bodies, academia, neighbourhood groups, and urban planners work together.	IPLCs, CSOs, knowledge centres, women's SHGs, and PRI function in tandem.	State, corporate, donor, and certification actors dominated; villagers had limited agenda-setting power.
	Context-specific governance	Practices are adapted to local agroecological zones and socio-cultural realities.	Designed around IPLC customs, tenure systems, and forest use norms.	Tailored to community needs and ecological functions of coastal zones.	Responds to Medellín's geography, climate, and vulnerability to heat islands.	Programmes integrate Wayanad's forest ecology and tribal food systems.	Project design under-read savannah use, grazing, fuelwood dependence, and useful-tree diversity.
	Coordination and equity challenges	Bureaucratic inertia, state-farmer misalignments persist in implementation.	Benefit-sharing inequities and land rights violations pose governance risks.	Balancing conservation and livelihoods remains a community negotiation.	Inequities in access to green zones remain across Medellín's urban poor.	Coordination gaps across health, forest, and ILK recognition remain.	Coordination favoured carbon and plantation goals; local access and benefit-sharing remained contested.
Nexus Governance	Integrative, holistic and transdisciplinary framings	Builds conceptual convergence across food, soil, water, and health systems through agroecology and farmer-centred epistemologies.	Combines Indigenous knowledge, carbon governance, biodiversity stewardship, and health linkages into an integrated landscape approach.	Integrates mitigation, biodiversity conservation, and livelihood improvement within a participatory, ecosystem-based PES framework.	Frames urban resilience through co-benefits across air quality, health, biodiversity, and ecological connectivity in spatially layered design.	Merges biocultural diversity, nutrition, ecosystem restoration, and women's agency through Indigenous and ecological framings.	Carbon and timber objectives dominated; livelihood, water, food, and health links were secondary.
	Inclusive approaches for diverse actor engagement	Involves RySS, CSOs, farmer federations, and marginalised women farmers in co-producing locally adapted knowledge systems.	Ensures participation of IPLCs in mapping, monitoring, benefit-sharing, and decision-making through recognised customary institutions.	Facilitates collective planning among village committees, local NGOs, and scientific partners with embedded community ownership.	Engages planners, neighbourhood groups, academics, and NGOs in co-design, with some civic bodies mediating community claims.	Anchored in collaborative platforms across tribal women's collectives, CSOs, healers, and biodiversity boards for co-governance.	Community activities existed, but did not amount to rights-based co-design.
	Considerations of equity, justice, and accountability	Targets tenancy farmers and the landless through inclusive entitlements and grievance redressal via SHGs and CRPs.	Addresses procedural injustices via FPIC protocols and REDD+ safeguards; engages in advocacy against tenure violations.	Deploys equitable benefit-sharing mechanisms and transparent revenue use for education, water, and infrastructure services.	Struggles with social exclusion and uneven access, prompting grassroots mobilisation for spatial justice in informal settlements.	Focuses on remedying intergenerational injustice in food and health systems by centring tribal women's voices and entitlements.	Safeguards did not prevent restricted access to land, fuelwood, grazing, and useful trees.

Dimension	Key Element	Andhra Pradesh CMNF[27–31]	Brazilian Amazon Indigenous REDD+[32–37]	Mikoko Pamoja[38–40]	Medellín's urban green corridors [41–46]	Wayanad food-health traditions[47–50]	Kachung Forest Project [104–110]
	Collaboration and coordination across scales and sectors	Enables coordinated delivery through agriculture, rural development, nutrition, and NRM departments, overcoming policy fragmentation.	Operates across forest, climate, biodiversity, and IPLC welfare institutions, with donor-state-community convergence under Amazon Fund.	Aligns NGOs, state institutions, and donors for joint forest stewardship and carbon governance under Plan Vivo standards.	Bridges planning, climate adaptation, health, and transport departments, with inter-agency and inter-scalar negotiation on land use.	Involves AYUSH, agriculture, biodiversity, and nutrition missions through layered and decentralised programme convergence.	Global carbon finance linked to national forestry, but weakly to local livelihood institutions.
	Adaptive, reflexive and experimental approaches	Uses farmer field schools and iterative learning platforms for adaptive design, supported by community-led experimentation.	Evolves through participatory carbon accounting, IPLC-led territorial monitoring, and feedback from PES design iterations.	Adjusts through community validation of carbon gains, revised planting strategies, and emerging co-benefit metrics for resilience.	Incorporates real-time monitoring, pilot adjustments, and citizen feedback loops to recalibrate greening strategies and access equity.	Draws on iterative co-learning between ILK holders and scientists through seasonal diet calendars and ecological indicators.	Reflexivity emerged mainly after criticism; initial design relied on technocratic baselines.
Integration of Governance Models	Integration of Governance Models	Blends decentralised, adaptive practices with convergence of rural schemes.	Integrates IPLC forest governance with carbon market mechanisms.	Combines community-based management with carbon-finance tools.	Unites formal city planning with grassroots urban ecology.	Bridges SHG governance with state and donor health and food initiatives.	State forestry, private plantation, and CDM finance integrated; customary governance remained marginal.
Financial & Technological Mechanisms	Climate Finance	Funded by state and central schemes; lacking carbon credit revenue.	Amazon Fund and donor-backed PES drive IPLC support.	Blue carbon credits fund restoration and community infrastructure.	City/donor co-funding with potential for climate finance.	State, donor, and CSO support but no carbon-linked finance yet.	CDM finance enabled planting and monitoring; community-benefit allocation remained insufficiently accountable.
	Innovative Instruments	Social audits, CRP-led extension, and farmer field schools.	PES, carbon trading, and ILK territorial monitoring tools.	Pioneers blue carbon credit trading in coastal East Africa.	Urban vertical gardens, native species greening, storm-buffer zones.	Rice branding, herbal value chains, community nurseries.	Carbon accounting, GIS, FSC certification, woodlots, and efficient stoves were deployed.
	Technology Transfer	Agroecological tools, SRI methods, and local seed systems shared.	Geospatial mapping, remote sensing merged with ILK systems.	Satellite-based monitoring + community reporting integrated.	Urban GIS tools and green design shared with municipalities.	ILK co-production with MSSRF and traditional practitioners.	Plantation silviculture and GIS transferred; local ecological knowledge was not equivalently recognised.
Ethical Frameworks	Ethics in Climate Governance	Values of dignity, ecological care, and intergenerational justice inform practice.	Grounded in climate justice and Indigenous rights-based governance.	Prioritises justice for communities maintaining carbon sinks.	Anchored in green equity, climate health, and urban well-being.	Guided by ethics of care, food dignity, and sustainability.	Global mitigation efficiency outweighed livelihood security and recognition justice.
	Worldviews and Value Systems	Rooted in Gandhian ecology, natural farming ethics, and simplicity.	ILK cosmologies and stewardship frame forest conservation.	Respect for ancestral marine practices underpins design.	Cultural respect for land and communal greenery fosters inclusion.	Biocultural ethics from tribal healing and diet systems.	Trees were valued mainly as carbon and timber, not as multifunctional livelihood resources.
	Earth System Justice (ESJ)	Emphasises procedural and distributive justice across agroecosystems.	Distributive and restorative justice through IPLC-led PES.	Balances community development with blue carbon protection.	Equitable access to ecosystem services and green space.	Food, land, healing justice across generations and genders.	Weak ESJ alignment: procedural, distributive, and recognition justice remained contested.
Equity, Justice & Social Inclusion	Recognition and Participation	Empowers women farmers and landless through SHGs and CRPs.	Supports IPLC recognition and FPIC throughout REDD+ cycle.	Community ownership and decision-making are reported as central to implementation.	Citizens involved in green corridor planning and monitoring.	Consensus-based decisions via SHGs and cultural organisations.	Local users, especially women fuelwood collectors, were insufficiently recognised as rights-holders.
	Distributive Justice	Aims to improve benefit flows to smallholders and vulnerable groups.	Carbon payments distributed via community-level governance.	Funds shared across schools, water, and equity initiatives.	Reported benefits include improved air quality, jobs and urban amenity	Reported benefits include income, nutrition and access to herbal resources	Carbon and timber benefits concentrated upward; access burdens fell on neighbouring communities.

**Dimension**	**Key Element**	**Andhra Pradesh CMNF**[27–31]	**Brazilian Amazon Indigenous REDD+**[32–37]	**Mikoko Pamoja**[38–40]	**Medellín's urban green corridors** [41–46]	**Wayanad food-health traditions**[47–50]	**Kachung Forest Project** [104–110]
	Environmental Justice Movements	Linked to national farmer movements and agroecology coalitions.	Part of IPLC-led forest and land rights resistance.	Blue justice framing for coastal rights and recognition.	Urban greening tied to justice in underserved communities.	Revival of Indigenous healing/food as cultural reclamation.	The project became a carbon-justice controversy through academic, NGO, and media scrutiny.
Indigenous & Local Knowledge (ILK)	Integration with Scientific Knowledge	Farmers' knowledge integrated with agroecology, SRI, and weather tools.	ILK combined with satellite and ecological research tools.	ILK validated via participatory carbon measurement and planning.	Urban greening benefits from community ecological knowledge.	ILK co-produced with MSSRF, validated in diet/health.	Remote sensing and expert baselines dominated; situated tree-use knowledge was marginalised.
	Role in Resilience and Adaptation	Traditional seed, crop, and soil knowledge supports resilience.	ILK critical for fire control, biodiversity, and risk alerts.	Supports biodiversity, water access, and nutrition recovery.	Adaptation benefits from community knowledge of plant resilience.	Used for climate-proof diets, agroecology, and healthcare.	Loss of useful trees, grazing, and fuelwood access weakened everyday resilience.
	Institutional Recognition	Supported through informal mechanisms and SHG processes.	Partially recognised under REDD+, but legally vulnerable.	Facilitated via project partnerships and PES rules.	Urban policy lacks formal ILK recognition.	ILK not yet fully institutionalised under Biodiversity Act.	ILK and customary use were not embedded in CDM design or monitoring.

Note. The table uses short case labels for readability. Full case descriptions and references are provided in the column headings or Supplementary References. The entries compare enabling conditions across cases; they do not rank the cases or imply equivalent evidence depth.

Table S8: Indicators and scoring framework for response options

Criterion	Indicator	Scoring Criteria
Impact on each element of the nexus	Biodiversity: change in state; change in function (component variables under each)	Scoring for change in state and change in function is separate (−3 to +3): +3 = increase in at least two component variables and decrease in none; +2 = increase in at least two and decrease in one; +1 = increase in at least one and decrease in no more than one; 0 = no change or cancelled-out change across the three components; −1 = decrease in at least one and increase in no more than one; −2 = decrease in at least two and increase in one; −3 = decrease in at least two and increase in none.
	Water (for humans): water quantity and quality across the annual cycle and during critical periods (four components)	Four-component rule (−3 to +3): +3 = increase in three or more components and decrease in none; +2 = increase in three and decrease in no more than one; +1 = increase in two and decrease in no more than one; 0 = increase in two and decrease in two; −1 = increase in one and decrease in no more than one; −2 = increase in one and decrease in two or more; −3 = no increase in any component.
	Water (for ecosystems): water quantity and quality across the annual cycle and during critical periods (four components)	Four-component rule (−3 to +3): +3 = increase in three or more components and decrease in none; +2 = increase in three and decrease in no more than one; +1 = increase in two and decrease in no more than one; 0 = increase in two and decrease in two; −1 = increase in one and decrease in no more than one; −2 = increase in one and decrease in two or more; −3 = no increase in any component.
	Food: access to healthy food; environmental performance of production; production	Single scale (−3 to +3): +3 high positive impact; +2 medium positive; +1 low positive; 0 no impact; −1 low negative; −2 medium negative; −3 high negative.
	Health: burden of disease or disability; well-being (mental, social, cultural)	Single scale (−3 to +3): +3 high positive impact; +2 medium positive; +1 low positive; 0 no impact; −1 low negative; −2 medium negative; −3 high negative.
	Health: access to quality healthcare (including environmentally supportive policies and practices)	Population-scope scale (−3 to +3): +3 positive impacts on entire populations or countries; +2 positive impacts on remote and vulnerable populations; +1 positive impacts on vulnerable populations only; 0 no impact; −1 negative impacts on vulnerable populations only; −2 negative impacts on remote and vulnerable populations; −3 negative impacts on entire populations or countries.
	Climate change – Mitigation potential	Based on $GtCO_2e\ yr^{-1}$ (−3 to +3): +3 > 3 $GtCO_2e$ reduced; +2 = 0.3–3 $GtCO_2e$ reduced; +1 = positive but < 0.3 $GtCO_2e$ reduced; 0 no impact; −1 = up to 0.3 $GtCO_2e$ increased; −2 = 0.3–3 $GtCO_2e$ increased; −3 > 3 $GtCO_2e$ increased.
	Climate change – Adaptation impact	Based on people affected or benefiting (−3 to +3): +3 positive impacts on >25 million people; +2 = 1–25 million; +1 = up to 1 million; 0 no impact; −1 negative impacts up to 1 million; −2 = 1–25 million; −3 >25 million negatively affected.
Social acceptability	Public support; effects on vulnerable groups	0 = no public support or no positive effect; 1 = low support or effect; 2 = medium support or effect; 3 = high support or effect.
Alignment with political priorities	Policy support; inclusion in national priorities	0 no policies or priorities; 1 few; 2 some; 3 many policies or priorities supporting the response option.
Technical feasibility	Technology Readiness Level (TRL)	TRL 1 basic principles observed; TRL 2 concept formulated; TRL 3 experimental proof of concept; TRL 4 validated in lab; TRL 5 validated in relevant environment; TRL 6 demonstrated in relevant environment; TRL 7 prototype demonstrated in operational environment; TRL 8 system complete and qualified; TRL 9 system proven in operational environment.
Equity	Distributional equity; Inclusion and/or recognition; Procedural equity	Distributional equity (−3 to +3): −3 unfair costs with no benefits; 0 balanced costs and benefits; +3 highly equal benefits with no costs. Inclusion and recognition (0–3): 0 none; 1 low; 2 medium (multiple rights or knowledge systems recognised); 3 high (comprehensive or Indigenous-led). Procedural equity (1–5): 1 informing; 2 consulting; 3 deciding together; 4 acting together; 5 supporting independent community interests.
Alignment with international frameworks	Kunming–Montreal GBF; SDGs; Paris Agreement	Per framework (1–3): 1 not referenced; 2 addresses at least one specific actionable target; 3 addresses multiple actionable targets.
Transformative change potential	Social, cultural and technical innovation; Structures and institutions; Views and values	Each sub-indicator scored 0–3: Innovation—0 none; 1 low; 2 medium; 3 high facilitation of new actions or practices. Structures and institutions—0 business-as-usual; 1 incremental change; 2 substantial change; 3 novel structures enabling innovations and power shifts. Views and values—0 none; 1 low; 2 medium; 3 high potential for value change.

Note: Scores indicate structured, evidence-informed judgements. They compare the direction, strength and consistency of available evidence across response options or policy instruments. They do not imply deterministic effects, universal transferability or direct causal proof. Where evidence is mixed, context-dependent or incomplete, the tables retain that uncertainty rather than converting it into a single definitive claim.

Table S9: Criteria for evaluating policies and procedures enabling climate response options

Instrument category	Score	Interpretive criteria
Legal and regulatory instruments	3	Fully institutionalised and operational across sectors, with clear mandates, resource allocation, procedural accountability, and evidence of implementation.
	2	Strong policy articulation with partial or uneven operationalisation. Nexus linkages acknowledged and cross-sectoral integration evident but not comprehensive.
	1	Legally recognised frameworks exist but are fragmented in coverage and uptake regarding nexus integration.
	0	Siloed approach that generates trade-offs or maladaptation due to competing priorities or power asymmetries inhibiting national integration.
Economic and financial instruments	3	Proven multi-sectoral alignment with high integrity standards and demonstrated co-benefits. Governance safeguards and equitable access embedded.
	2	Well-designed instruments targeting nexus co-benefits. Contextually effective but not yet scaled or fully safeguarded.
	1	Initial alignment with nexus priorities. Innovation visible, but regulatory clarity, institutional buy-in, or equity frameworks still emerging.
	0	Economically viable but socially or ecologically contentious; risks incentivising nature-negative practices under weak safeguards.
Rights-based and customary instruments	3	Fully recognises customary rights, enables community agency, and is institutionally anchored. Promotes community ownership.
	2	Promotes equity and synergy through participatory mechanisms, though enforcement or resource asymmetries persist.
	1	Partially inclusive; symbolic or pilot interventions not supported by legal safeguards or financial backing.
	0	Nominal references to IPLCs and customary rights without formal recognition or implementation.
Socio-cultural approaches	3	Deeply embedded in cultural practices; supports behavioural change, co-management, and intergenerational empowerment.
	2	Participatory and co-designed with high legitimacy but requires stronger institutional support or resourcing.
	1	Awareness-oriented initiatives with strong engagement but lacking systemic integration.
	0	Conceptually aligned with sustainability but lacking institutional mandate or strategic anchoring; superficial participation without authority transfer.

Note. Scores are interpreted within each instrument category. A score of 0 does not always indicate the same type of limitation. It indicates weak or absent enabling capacity relative to the institutional function of that category.

Table S10: Criteria for assessing climate response options for Earth System Justice compatibility and nexus governance synergy

Score	Earth System Justice (ESJ) Compatibility	Nexus Governance Synergy
**4**	**Transformative** – Reconfigures structural power relations; institutionalises distributive, procedural, recognition, and intergenerational justice; embeds Indigenous and local knowledge; delivers equitable outcomes across communities and generations.	**Systemic** – Fully institutionalised adaptive governance across biodiversity, water, food, health, and climate domains; strong coordination mechanisms; sustained cross-sectoral co-benefits and systemic trade-off management.
**3**	**Strong** – Integrates procedural, distributive, and intergenerational justice principles in design and implementation; demonstrates active stakeholder inclusion and measurable equity outcomes.	**High** – Robust multi-sectoral integration; clear coordination mechanisms; effective trade-off management; substantial and demonstrable nexus synergies.
**2**	**Moderate** – Incorporates procedural safeguards and partial distributive justice; recognition justice or intergenerational considerations remain incomplete or uneven.	**Moderate** – Engagement across multiple nexus domains (typically two to three); emerging coordination; mixed management of trade-offs and co-benefits.
**1**	**Limited** – Justice principles acknowledged but weakly operationalised; limited redistribution, inclusion, or recognition mechanisms.	**Low** – Minimal cross-sectoral coordination; weak synergy generation; governance remains largely sector-bound.
**0**	**Incompatible** – Reinforces inequities or exclusion; exacerbates environmental or social injustice.	**Fragmentary** – Operates strictly within silos; intensifies cross-sectoral trade-offs; lacks nexus awareness.

Note. The criteria assess the justice and governance orientation of response options as interpreted through the available evidence. They do not imply that all implementations of a response option achieve the same level of ESJ compatibility or nexus governance synergy.

Scores indicate structured, evidence-informed judgements. They compare the direction, strength and consistency of available evidence across response options or policy instruments. They do not imply deterministic effects, universal transferability or direct causal proof. Where evidence is mixed, context-dependent or incomplete, the tables retain that uncertainty rather than converting it into a single definitive claim.

Table S11: Evidence base, source coverage, confidence, and limitations for the 15 climate response options

This Supplementary Material reports the evidence base used to interpret the fifteen climate response options assessed in the manuscript. The evidence base draws directly from citations used in the corresponding Chapter 5.5 response-option sections and their data-management reports. The source counts report distinct source entries cited in those sections. They do not represent the total number of independent studies identified through a systematic review. A single source entry may synthesise several studies, while separate entries may draw on overlapping evidence. Table S11 therefore maps the bounded assessment evidence base rather than estimating the size of the global literature.

Response option	Representative evidence base	Number of sources used	Evidence type	Geographical spread	Confidence	Key limitation
C01: Increase soil organic carbon	[111–124]	14	Peer-reviewed synthesis, IPBES/IPCC-derived assessment evidence, soil-carbon and land-degradation studies.	Global managed land, especially croplands, grazing lands and managed forests.	Well established	Carbon gains saturate and can reverse through poor management, fire, drought or land-use change; nutritional and food-access effects are more strongly mediated than soil and water benefits.
C02: Sustainable intensification	[122,125–141]	27	Peer-reviewed agricultural studies, synthesis evidence and global food-systems assessment evidence.	Global and regional agricultural systems, with emphasis on intensively managed and smallholder production systems.	Established but incomplete	Higher productivity can reduce land pressure only when governance prevents rebound effects, input intensification, irrigation stress and biodiversity loss.
C03: Integrated multitrophic aquaculture	[142–149]	12	Peer-reviewed aquaculture research, FAO/UNEP evidence and coastal food-system synthesis.	Marine and coastal aquaculture systems, with evidence from Asia, Europe and other aquaculture regions.	Established but incomplete	Benefits depend on species mix, feed sources, ecological carrying capacity, disease management, water quality and siting.
C04: Wetland conservation and restoration	[122,150–161]	19	Global assessment evidence, wetland and peatland studies, nature-based solution synthesis and restoration literature.	Global wetlands, peatlands, floodplains and coastal wetland systems.	Established but incomplete	Carbon, biodiversity and water gains vary by wetland type; methane dynamics, land claims and disease-vector concerns require local assessment.
C05: Offshore wind power	[162–179]	23	Peer-reviewed marine-energy, biodiversity, fisheries and energy-transition evidence.	Global offshore-energy literature, with strong empirical grounding from Europe and expanding evidence from other coastal regions.	Established but incomplete	Marine impacts depend on siting, construction noise, benthic disturbance, cumulative effects, fisheries displacement and marine spatial planning.
C06: Solar photovoltaics on land	[160,180–199]	35	IPCC-derived energy-transition evidence, solar-siting studies, agrivoltaics evidence and land-use literature.	Global, with evidence from utility-scale solar, rural energy access, agrivoltaics and land-siting contexts.	Established but incomplete	Land competition, habitat conversion, water requirements, panel-waste management and tenure conflicts can offset benefits if siting safeguards are weak.
C07: Sustainable bioeconomy	[200–219]	52	Peer-reviewed circular-bioeconomy, biomass, industrial ecology and policy-synthesis evidence.	Global bioeconomy literature, with relevance to biomass-producing landscapes, industrial value chains and circular resource systems.	Well established for circularity potential; established but incomplete for distributional outcomes	Circular resource use does not automatically prevent biomass pressure, land competition, water stress or unequal capture of value-chain gains.
C08: Reduce short-lived climate pollutants	[193,220–240]	46	Atmospheric science, health-impact modelling, air-pollution evidence and global assessment literature.	Global, across energy, transport, agriculture, waste, household energy and industrial emissions sources.	Well established for climate and health; established but incomplete for biodiversity and food-system pathways	Climate and health benefits are rapid, but biodiversity, water and food effects are often mediated and pollutant-specific.
C09: Multi-hazard early warning systems	[220,241–250]	23	Disaster-risk, climate-service, health-warning, occupational-safety and national adaptation evidence.	Global, with strong relevance for hazard-prone regions, small islands, coastal zones, drylands, heat-prone cities and flood-prone basins.	Well established	Effectiveness depends on last-mile communication, trust, institutional capacity, maintenance finance and inclusion of marginalised groups.

C10: Global cooperation for finance and technology	[79,251–264]	17	Climate-finance, technology-transfer, biodiversity-finance and institutional-policy evidence.	Global, especially North-South finance, technology cooperation, capacity-building and biodiversity-climate funding contexts.	Well established for enabling role; established but incomplete for equity outcomes	Finance and technology flows remain constrained by inadequate scale, debt burdens, access barriers, accountability gaps and geopolitical asymmetries.
C11: Agroecology	[80,84,265–280]	52	Agroecology and agroforestry synthesis, peer-reviewed empirical studies, ILK-related evidence and case evidence.	Global agroecological and agroforestry systems, with strong evidence from smallholder and Global South contexts, including Andhra Pradesh.	Well established for biodiversity-soil-food synergies; established but incomplete for scaling pathways	Benefits vary by agroecological zone, farmer support, tenure, markets, labour burdens, extension capacity and yield-transition dynamics.
C12: Forest-based practices to address climate change	[39,122,157,281–295]	86	Forest mitigation, REDD+, forest restoration, biodiversity, hydrology, tenure and Amazon monitoring evidence.	Global forest systems, with detailed case evidence from the Brazilian Amazon and broader tropical forest contexts.	Established but incomplete	Carbon and biodiversity outcomes depend strongly on forest type, permanence, tenure security, safeguards, leakage, fire risk and recognition of Indigenous and local rights.
C13: Restoration of coastal and marine ecosystems for carbon sequestration	[296–310]	50	Blue-carbon synthesis, coastal ecosystem science, case studies, restoration evidence and policy/grey literature.	Global mangrove, seagrass and salt-marsh systems, with detailed case evidence from Senegal's Sine-Saloum delta.	Established but incomplete	Blue-carbon benefits depend on site suitability, tenure, permanence, hydrology, monitoring capacity and livelihood safeguards.
C14: Urban nature-based solutions	[95,257,268,311–327]	43	Urban climate adaptation, public-health, green-infrastructure, biodiversity and urban-governance evidence.	Global urban systems, with detailed case evidence from Medellin and wider urban nature-based solution literature.	Established but incomplete	Cooling, health and biodiversity gains can be uneven; green gentrification, maintenance finance and spatial inequity remain persistent risks.
C15: Sustainable healthy diets	[268,328–341]	23	Global modelling, nutrition and health evidence, food-systems assessment, public procurement and dietary-transition case evidence.	Global dietary-change literature, with case evidence from Arara Indigenous territories and Los Angeles County public food procurement.	Well established for health and emissions; established but incomplete for equitable transition pathways	Affordability, cultural acceptability, access to diverse foods, public procurement design and distributional effects shape feasibility and justice outcomes.

Interpretive note. The evidence-base column lists principal or representative citations from the relevant Chapter 5.5 sections. Where sections contain many citations, the table identifies the evidence base without reproducing the complete chapter bibliography. Source counts should not be compared as measures of independent-study volume or evidentiary strength across response-option types. Different literatures emphasise different outcomes, mechanisms, regions, and risks, which creates evidence-focus bias. The table should be read with Chapter 5.5 and the scoring frameworks in Tables S8–S10. Confidence categories indicate the strength, consistency, and agreement of the evidence used for this analysis. They do not imply universal effect sizes or invariant outcomes across places.

Suggested manuscript cross-reference. To make the evidence base behind these directional judgements transparent, Table S11 reports, for each response option, the main evidence base, the number of chapter-cited source entries, the evidence type, the geographical spread, the confidence, and the key limitation.

Methodological limitations

The assessment did not use blinded independent parallel coding procedures. Blinded coding requires reviewers to classify evidence without knowing other coders' decisions. The author team instead used deliberative calibration within the established Chapter 5.5 assessment process. The study did not calculate inter-rater reliability, which measures agreement among independent coders. It also did not conduct formal numerical sensitivity analysis to test alternative scoring assumptions. These procedures could strengthen future assessments, but the present analysis reports no results from them.

Comparisons between response-option types require two further methodological qualifications. Evidence-focus bias arises when separate literatures emphasise different outcomes, mechanisms, regions, or risks. The observed patterns may therefore partly reflect how each research field frames and measures its focal response options. The selection boundary also limits comparison because the fifteen options were chosen for cross-nexus traceability and analytical relevance, rather than exhaustive representation. Accordingly, the tables report evidence-supported comparative tendencies, not intrinsic rankings of response-option quality.

Table S 12: Selection-boundary audit for long-list climate response options, related to STAR Methods and Tables 1 and 4

This table defines the selection boundary. It draws on the long-list assessment by Smith et al. (2025) and identifies response options not analysed as separate units in the manuscript. Exclusion does not imply ineffectiveness. Most exclusions reflect overlap with selected options, narrower sectoral mechanisms, weaker cross-nexus traceability, or limited relevance to the governance-and-justice explanation tested in the paper. The table also identifies selection-bias risks and the safeguards used to reduce them. Comparisons remain subject to evidence-focus bias because separate literatures examine different outcomes and mechanisms. The C-codes refer to the 15 selected response options in the manuscript and Table S 1. Indigenous and local knowledge is abbreviated as ILK.

Long-list option/category not examined	Reason for exclusion	Risk of selection bias	How bias was mitigated
Afforestation as a stand-alone option	Overlaps with C12, but stand-alone tree planting has distinct biome, species, water, and access risks.	Moderate. Tree-planting risks could be understated.	C12 covers forest practices. The Kachung counter-case shows how weak tenure, baseline, access, and benefit-sharing safeguards can undermine forest-carbon legitimacy.
Agricultural diversification	Overlaps with C02 and C11; its effects operate through crop, seed, livestock, and livelihood diversification within wider agricultural transitions.	Low to moderate. Diversity-centred farming pathways may be less visible.	C02 and C11 retain diversity, resilience, and livelihood mechanisms.
Agroforestry as a stand-alone option	Substantially overlaps with C11 and C12, which already cover land-tree-food-water mechanisms.	Moderate. Tree-based agricultural transitions could be understated.	C11 and C12 retain agroforestry-related mechanisms and avoid equating forest-based practices with plantations.
Biochar addition to soil	Overlaps with C01, but requires separate life-cycle, feedstock, pyrolysis, and pollution assessment.	Moderate. Engineered soil-carbon pathways may be underrepresented.	C01 covers soil-carbon benefits and permanence limits as a soil-carbon family rather than a single practice.
Carbon storage in seabed	A highly specific marine carbon-dioxide-removal option with uncertain feasibility, monitoring, ecological disturbance, and governance.	Low to moderate. Its evidence base is narrower than for ecosystem restoration and agricultural options.	C13 retains marine carbon and blue-carbon issues, while engineered marine storage is not used to support broad governance claims.
Enhanced mineral weathering	An engineered carbon-dioxide-removal option with mining, grinding, transport, energy, and ecological uncertainties.	Moderate. Engineered mitigation options may be underrepresented.	C05, C06, C07, and C08 retain technology-centred and infrastructure-centred options, so the set is not exclusively ecosystem-centred.
Enhanced urban food systems	Partly overlaps with C14 and C15, through urban planning, food access, land availability, and diet transitions.	Low to moderate. Urban food-system adaptation may be less visible.	C14 captures urban ecological infrastructure; C15 captures diet-related food-system transformation.
Fire management	Context-specific risk-governance option that overlaps with C12 and C09.	Moderate. Wildfire governance remains important for forest, health, and climate outcomes.	C09 and C12 retain anticipatory risk, forest management, and ecosystem-governance mechanisms without generalising across all fire contexts.
Improved and sustainable forest management	Folded into C12 to avoid duplicating forest stewardship, carbon, biodiversity, and livelihood mechanisms.	Low to moderate. Aggregation can obscure differences among conservation, restoration, reduced deforestation, and production forestry.	C12 functions as an aggregate forest-governance option, while the cases distinguish enabling and contested forest-carbon pathways.
Improved cropland management	Overlaps with C01, C02, and C11 through soil, nutrient, and water-management pathways.	Low. Core mechanisms remain represented.	C01, C02, and C11 retain agricultural management effects.
Improved grazing land management	A narrower livestock-land option with pasture, stocking, methane, soil, and livelihood dynamics.	Moderate. Pastoral and rangeland transitions may be underrepresented.	C02 and C11 retain land-management pathways; C15 covers demand-side livestock-consumption implications.
Improved livestock management	A sector-specific option requiring dedicated analysis of methane, feed, animal health, land demand, and diet shifts.	Moderate. Livestock mitigation and adaptation pathways could be understated.	C08 covers methane as a short-lived climate pollutant; C15 addresses diet shifts and health implications.
Integrated coastal zone management	A broad coastal governance approach that extends beyond the blue-carbon and restoration focus of C13.	Moderate. Coastal planning instruments may be underrepresented.	C13 retains coastal restoration and blue-carbon mechanisms; the governance discussion recognises spatial planning and coordination.
Integrated water resource management	An enabling governance framework rather than a bounded climate response option.	Moderate. Water governance may appear less visible as a stand-alone pathway.	Water effects are assessed across response options; policy-instrument analysis captures cross-sectoral coordination.
Management for biodiversity and ecosystem services	Too broad for mechanism-specific comparison and overlaps with conservation, restoration, and nature-based options.	Low to moderate. Broad ecosystem-management benefits could be undercounted.	C04, C12, C13, and C14 represent specific ecosystem-management pathways.
Management of coastal and marine ecosystems beyond restoration	Partly represented through C13, while broader fisheries governance and coastal-use regulation were not treated separately.	Moderate. Marine governance beyond blue carbon may be underrepresented.	C13 keeps the focus on coastal and marine restoration for carbon sequestration, and the narrower scope is explicit.
Management of food supply chains	Overlaps with food-waste reduction, sustainable healthy diets, and circular bioeconomy.	Moderate. Food-chain efficiency may be underrepresented.	C15 retains food-system demand and nutrition pathways; C07 retains circular resource-use pathways.

Long-list option/category not examined	Reason for exclusion	Risk of selection bias	How bias was mitigated
Management of invasive species/encroachment	A biodiversity-centred adaptation option with context-specific effects and less direct mitigation evidence.	Low to moderate. Some biodiversity-health-water interactions may receive less attention.	C04, C12, C13, and C14 retain ecosystem-restoration mechanisms; the manuscript does not claim exhaustive biodiversity coverage.
More sustainable ocean fisheries, aquaculture, and dietary shifts beyond integrated multitrophic aquaculture	C03 represents a bounded aquaculture pathway; broader fisheries and marine diet-shift pathways were not separated.	Moderate. Sustainable fisheries may be underrepresented relative to aquaculture.	C03 retains marine food-production synergies; C13 captures coastal ecosystem restoration.
Nature conservation as a stand-alone option	An umbrella category overlapping with wetland, forest, coastal, marine, and urban nature-based options.	Low. Conservation mechanisms remain present.	C04, C12, C13, and C14 retain conservation and restoration pathways across landscapes and seascapes.
Nature-based Indigenous and local knowledge as a stand-alone option	Treated as a cross-cutting epistemic and governance condition, not a discrete technical intervention.	Low to moderate. ILK-specific contributions could become less visible.	Indigenous and local knowledge is analysed as a knowledge, justice, and legitimacy condition in the manuscript and cases.
Reduced conversion of grassland to cropland	A narrower land-protection option with food-system trade-offs.	Moderate. Grassland conservation and pastoral landscapes may be underrepresented.	C02, C11, and C12 retain land-use trade-off analysis, while the limitation prevents claims of exhaustive coverage.
Reduced deforestation and degradation	Aggregated within C12 rather than treated as a separate REDD+ or avoided-deforestation option.	Low to moderate. Aggregation may mask differences among avoided deforestation, restoration, and plantation-based options.	C12 includes Indigenous REDD+ evidence and the Kachung counter-case to compare enabling and contested forest-carbon pathways.
Reduced post-harvest food losses	Overlaps with sustainable diets and food-system demand reduction.	Moderate. Food-loss reduction may be underrepresented as a mitigation pathway.	C15 captures diet and food-system transformation; food loss and waste remain associated pathways.
Reduced soil erosion	Overlaps with C01, C02, and C11 through soil health, cover, organic matter, and agroecological management.	Low. Soil and water-retention mechanisms remain represented.	C01, C02, and C11 preserve core soil-function mechanisms.
Reforestation and forest restoration as a stand-alone option	Folded into C12 to avoid creating a full forestry typology outside the paper's analytical scope.	Moderate. Restoration may differ from avoided deforestation or plantations.	The Indigenous REDD+ evidence and Kachung counter-case reduce successful-case bias and expose design differences.
Restoration and reduced conversion of peatlands as a stand-alone option	Partly represented through C04, which treats wetland restoration as a broader water-carbon-biodiversity option.	Low to moderate. Peatland methane, fire, and water-table issues may receive less attention.	C04 retains wetland restoration and hydrological regulation; peatland-specific claims are avoided.
Rewilding	A context-specific restoration option with possible land-use and food-production trade-offs.	Moderate. Rewilding debates may be underrepresented.	Selected ecosystem options cover restoration, conservation, and governance trade-offs without generalising to all restoration paradigms.
Bioenergy and biofuels	A biomass-based mitigation option with strong land, water, biodiversity, food, and livelihood trade-offs.	High. Exclusion could make the selected set appear more favourable.	C07 discusses biomass demand and land-governance constraints; infrastructure options with trade-offs are retained.
Bioenergy with carbon capture and storage (BECCS)	A contested land-energy-carbon-removal pathway requiring dedicated assessment of biomass, land, water, and storage.	High. BECCS exclusion could understate high-trade-off mitigation pathways.	The exclusion is disclosed; biomass and carbon-removal trade-offs are addressed through C07 and design limitations.
Energy demand management	A high-relevance demand-side energy option with less direct biodiversity-water-food-health evidence than food-system demand options.	Moderate. Low-risk energy-demand transitions may be understated.	C15 represents societal demand-side change; C08 captures health gains from sectoral emissions reduction.
Energy storage for low-carbon grids	An enabling infrastructure option mediated by minerals, siting, chemistry, storage type, grids, and recycling.	Moderate. Grid-enabling constraints may be underrepresented.	C05 and C06 show renewable-infrastructure trade-offs; the manuscript avoids inferring full energy-system effects from direct-effect scoring.
Energy system integration	A system-level enabling condition mediated through grid design, demand, storage, sector coupling, and governance.	Moderate. Whole-system energy design may be understated.	The Methods separate direct effects from systemic mediation; finance, technology, and governance are treated as enabling conditions.
Fossil fuels with carbon capture and storage	A fossil-based abatement option with continuing extraction, infrastructure, leakage, and air-pollution concerns.	High. Exclusion could bias comparison toward lower-risk options.	The exclusion is explicit; selected technology options are not presented as a ranked endorsement of all low-carbon systems.
Geothermal energy	A renewable option with site-specific water, seismic, mineral, and ecosystem effects.	Low to moderate. Renewable-energy diversity is reduced.	Offshore wind and land-based solar photovoltaics represent the energy-transition category.
Hydroelectric power	A mature renewable option with major river-flow, biodiversity, sediment, displacement, and food-system implications.	High. Water-energy-biodiversity conflicts could be less visible.	Infrastructure sections analyse land and water trade-offs; the exclusion table keeps this limitation explicit.
Nuclear power	A low-carbon electricity option with mining, waste, cost, safety, and social-acceptability issues.	Moderate to high. Non-renewable low-carbon options may be underrepresented.	Selected energy options are not used to represent all low-carbon electricity systems.

Long-list option/category not examined	Reason for exclusion	Risk of selection bias	How bias was mitigated
Ocean-based renewable energy beyond offshore wind	Includes wave, tidal, ocean-thermal, and salinity-gradient technologies with less mature evidence.	Low to moderate. Emerging marine energy options may be underrepresented.	C05 retains offshore renewable-energy and marine spatial-planning trade-offs.
Resilient power infrastructures/systems	An enabling infrastructure option mediated through reliability, decentralisation, storage, and grid governance.	Moderate. Adaptation benefits of resilient energy infrastructure may be understated.	Finance, technology, and institutional coherence are treated as enabling conditions rather than direct effects.
Solar photovoltaics offshore	Overlaps with land-based solar photovoltaics and offshore energy mechanisms.	Low to moderate. Offshore-solar effects are not separately captured.	C06 captures solar land-use issues; C05 captures offshore energy and marine-ecosystem interactions.
Wind power onshore	Overlaps with offshore wind but has distinct landscape, biodiversity, community, and siting implications.	Moderate. Onshore siting conflicts may be underrepresented.	C05 retains wind-energy infrastructure concerns; C06 captures land-based spatial competition.
Change in construction methods and materials	A built-environment decarbonisation option with mediated material and embodied-carbon effects.	Low to moderate. Built-material transitions may be less visible.	C09 and C14 represent urban and infrastructure transitions; C07 partly captures material and circularity pathways.
Efficient appliances	A household energy-efficiency option with mostly mediated biodiversity-water-food-health effects.	Low. It is a narrower demand-side energy option.	C15 represents demand-side transformation; C08 captures mitigation and health effects from emissions reduction.
Electromobility	A transport decarbonisation option requiring separate analysis of minerals, infrastructure, energy, and justice.	Moderate. Transport-transition issues may be underrepresented.	C08 captures air-pollution and health gains; C05 and C06 capture infrastructure and siting trade-offs.
Energy-efficient buildings	Overlaps with construction, appliances, and urban-planning measures.	Low to moderate. Built-environment energy options may be less visible.	C14 covers urban adaptation and health co-benefits; C08 covers emissions and air-quality effects.
Green mobility	A transport behaviour and infrastructure option with less direct cross-nexus traceability.	Low to moderate. Active mobility and air-quality benefits may be less visible.	C08 and C14 retain health, air-quality, and urban-environment pathways.
Sustainable land-use and urban planning	A broad enabling planning category that overlaps with C14 and governance analysis.	Moderate. Planning mechanisms may be underrepresented if not explicit.	C14 embeds urban planning, while the governance sections analyse spatial coordination and equity.
Urban green/blue infrastructure	Subsumed within C14 as a component of urban nature-based solutions.	Low. It is directly represented within C14.	C14 includes green and blue infrastructure, cooling, flood buffering, biodiversity, and health pathways.
Waste prevention, minimisation, and management	Overlaps with circular bioeconomy, reduced food waste, and short-lived climate pollutants.	Moderate. Waste-sector mitigation and health co-benefits may be underrepresented.	C07 captures circularity; C08 captures short-lived pollutants; C15 captures diet-related food-system demand.
Carbon-neutral manufacturing	A broad industrial decarbonisation category requiring sector-specific disaggregation.	Low to moderate. Industrial mitigation may be underrepresented.	C07 and C08 represent industrial transition; the manuscript avoids claims about all industrial decarbonisation pathways.
Direct air carbon capture and storage	An engineered carbon-dioxide-removal pathway with energy, storage, land, water, and governance implications.	Moderate to high. Engineered carbon-removal options may be underrepresented.	The exclusion is explicit, and the manuscript does not claim comprehensive coverage of carbon-removal technologies.
Direct air carbon capture and utilisation	Depends on product pathways, energy source, carbon permanence, and market use.	Moderate. Carbon-utilisation pathways are not represented in detail.	C07 captures circular resource-use logic; C05, C06, and C08 represent technology-centred mitigation.
Eco-industrial parks	An industrial ecology option with localised resource-sharing mechanisms and context-specific evidence.	Low to moderate. Local industrial-ecology pathways may be less visible.	C07 retains circularity and resource efficiency; governance analysis captures institutional coordination.
Green innovations in processes, techniques, systems, and products	Too broad to function as a bounded response option and duplicates several technology pathways.	Low. Innovation is excluded only as a separate scoring unit.	Innovation remains present through technology, finance, and implementation conditions.
Green supply chain management	A corporate and supply-chain option with diffuse effects across sectors.	Low to moderate. Supply-chain governance may be less visible.	C07 and C15 retain production-consumption linkages; policy instruments cover disclosure, finance, and accountability.
Industrial symbiosis	A resource-exchange pathway overlapping with C07 and circularity.	Low. The core circularity mechanism remains represented.	C07 retains circular resource-use and waste-valorisation mechanisms.
Materials efficiency	A demand-side industrial option mediated through manufacturing, consumption, waste, and supply chains.	Moderate. Material-demand reduction may be underrepresented.	C07 captures circular resource use; C15 captures demand-side transformation.
Behavioural nudges for sustainability	A broad behavioural instrument with variable effect sizes and context-dependent durability.	Low to moderate. Behavioural-policy mechanisms may be less visible.	C15 represents demand-side societal transformation; governance analysis covers procedural and institutional conditions for behaviour change.
Reduced food waste by consumers and retailers	A food-system demand and efficiency option folded into the broader C15 pathway.	Moderate. Food-waste mitigation may be underrepresented.	C15 can acknowledge food loss and waste as a complementary pathway; this table prevents overstating the scope of diet analysis.

Note: The entries in this table are long-list options or aggregated option/category entries. They are not intended as a one-to-one subtraction from the full long-list count. Some entries combine related options to make the selection boundary clearer. Exclusion from the manuscript does not imply low climate relevance. It indicates that the option was either represented within a selected response option, narrower than the manuscript's analytical unit, weakly traceable across the biodiversity-water-food-health nexus, or less suited to the governance-and-justice explanation examined here.

References:

1. Burke, M., Davis, W.M., and Diffenbaugh, N.S. (2018). Large potential reduction in economic damages under UN mitigation targets. Nature *557*, 549–553. https://doi.org/10.1038/s41586-018-0071-9.

2. Rogelj, J., Huppmann, D., Krey, V., Riahi, K., Clarke, L., Gidden, M., Nicholls, Z., and Meinshausen, M. (2019). A new scenario logic for the Paris Agreement long-term temperature goal. Nature *573*, 357–363. https://doi.org/10.1038/s41586-019-1541-4.

3. Pittock, J. (2011). National Climate Change Policies and Sustainable Water Management: Conflicts and Synergies. Ecology and Society *16*.

4. Gupta, H., Kohli, R.K., Ahluwalia, A.S., and Pal, P. (2014). Climate change and biodiversity: Analysis of the policy framework. Environmental Policy and Law *44*, 539–544.

5. Rasul, G., and Sharma, B. (2016). The nexus approach to water–energy–food security: an option for adaptation to climate change. Climate Policy *16*, 682–702. https://doi.org/10.1080/14693062.2015.1029865.

6. Tittensor, D.P., Beger, M., Boerder, K., Boyce, D.G., Cavanagh, R.D., Cosandey-Godin, A., Crespo, G.O., Dunn, D.C., Ghiffary, W., Grant, S.M., et al. (2019). Integrating climate adaptation and biodiversity conservation in the global ocean. Science Advances *5*, eaay9969. https://doi.org/10.1126/sciadv.aay9969.

7. Schmidt-Traub, G., Locke, H., Gao, J., Ouyang, Z., Adams, J., Li, L., Sala, E., Shaw, M.R., Troëng, S., Xu, J., et al. (2021). Integrating climate, biodiversity, and sustainable land-use strategies: innovations from China. National Science Review *8*, nwaa139. https://doi.org/10.1093/nsr/nwaa139.

8. SDSN (2021). The integration of biodiversity and climate objectives in land-use policy SDSN Policy Brief (Sustainable development solutions network and The Food and Land Use Coalition).

9. Fuso Nerini, F., Sovacool, B., Hughes, N., Cozzi, L., Cosgrave, E., Howells, M., Tavoni, M., Tomei, J., Zerriffi, H., and Milligan, B. (2019). Connecting climate action with other Sustainable Development Goals. Nature Sustainability *2*, 674–680. https://doi.org/10.1038/s41893-019-0334-y.

10. Soergel, B., Kriegler, E., Weindl, I., Rauner, S., Dirnaichner, A., Ruhe, C., Hofmann, M., Bauer, N., Bertram, C., Bodirsky, B.L., et al. (2021). A sustainable development pathway for climate action within the UN 2030 Agenda. Nature Climate Change *11*, 656–664. https://doi.org/10.1038/s41558-021-01098-3.

11. Bhattarai, K., and Conway, D. (2021). The Environment. Advances in Asian Human-Environmental Research, 115–199. https://doi.org/10.1007/978-3-030-50168-6_3.

12. Fuldauer, L.I., Thacker, S., Haggis, R.A., Fuso-Nerini, F., Nicholls, R.J., and Hall, J.W. (2022). Targeting climate adaptation to safeguard and advance the Sustainable Development Goals. Nature Communications *13*. https://doi.org/10.1038/s41467-022-31202-w.

13. Mombauer, D., Link, A.-C., and van der Geest, K. (2023). Addressing climate-related human mobility through NDCs and NAPs: State of play, good practices, and the ways forward. Frontiers in Climate *5*. https://doi.org/10.3389/fclim.2023.1125936.

14. TCFD (2022). Task Force on Climate-related Financial Disclosures.

15. Harvey, C.A., Chacón, M., Donatti, C.I., Garen, E., Hannah, L., Andrade, A., Bede, L., Brown, D., Calle, A., Chará, J., et al. (2014). Climate-Smart Landscapes: Opportunities and Challenges for Integrating Adaptation and Mitigation in Tropical Agriculture. Conservation Letters *7*, 77–90. https://doi.org/10.1111/conl.12066.

16. Scherr, S.J., Shames, S., and Friedman, R. (2012). From climate-smart agriculture to climate-smart landscapes. Agric & Food Secur *1*, 12. https://doi.org/10.1186/2048-7010-1-12.

17. Druckenmiller, H. (2022). Accounting for ecosystem service values in climate policy. Nat. Clim. Chang. *12*, 596–598. https://doi.org/10.1038/s41558-022-01362-0.

18. Gupta, H., and Dube, L.C. (2021). Benefits of Evaluating Ecosystem Services for Implementation of Nature-based Solutions Under the Paris Agreement. In Social-Ecological Systems (SES): From Risks and Insecurity to Viability and Resilience, M. Behnassi, H. Gupta, M. El Haiba, and G. Ramachandran, eds. (Springer International Publishing), pp. 39–56. https://doi.org/10.1007/978-3-030-76247-6_2.

19. Mäler, K.-G., Aniyar, S., and Jansson, Å. (2008). Accounting for ecosystem services as a way to understand the requirements for sustainable development. Proceedings of the National Academy of Sciences *105*, 9501–9506. https://doi.org/10.1073/pnas.0708856105.

20. Gupta, H., and Dube, L.C. (2018). Addressing biodiversity in climate change discourse: Paris mechanisms hold more promise. International Forestry Review *20*, 104–114. https://doi.org/10.1505/146554818822824282.

21. Cook, G., Zakkour, P., Neades, S., and Dixon, T. (2024). CCS under Article 6 of the Paris Agreement. International Journal of Greenhouse Gas Control *134*, 104110. https://doi.org/10.1016/j.ijggc.2024.104110.

22. Maltais, A., and Nykvist, B. (2020). Understanding the role of green bonds in advancing sustainability. Journal of Sustainable Finance & Investment, 1–20. https://doi.org/10.1080/20430795.2020.1724864.

23. Ning, Y., Cherian, J., Sial, M.S., Álvarez-Otero, S., Comite, U., and Zia-Ud-Din, M. (2022). Green bond as a new determinant of sustainable green financing, energy efficiency investment, and economic growth: a global perspective. Environ Sci Pollut Res. https://doi.org/10.1007/s11356-021-18454-7.

24. Agrawal, A., and Angelsen, A. (2009). Using community forest management to achieve REDD+ goals. In Realising REDD+ National strategy and policy options, A. Angelsen, M. Brockhaus, M. Kanninen, E. Sills, W. D. Sunderlin, and S. Wertz-Kanounnikoff, eds. (Center for International Forestry Research).

25. Ball, S., and Makala, J. (2014). Making REDD+ work for communities and forests: three shared lessons for project designers (IIED).

26. Dube, L.C. (2019). Conserving Carbon and Biodiversity Through REDD+ Implementation in Tropical Countries. In Climate Change, Food Security and Natural Resource Management, M. Behnassi, O. Pollmann, and H. Gupta, eds. (Springer International Publishing), pp. 281–297. https://doi.org/10.1007/978-3-319-97091-2_15.

27. Wallbott, L., Siciliano, G., and Lederer, M. (2019). Beyond PES and REDD+: Costa Rica on the way to climate-smart landscape management? Ecology and Society *24*.

28. Le, T.-A.T., Vodden, K., Wu, J., Bullock, R., and Sabau, G. (2024). Payments for ecosystem services programs: A global review of contributions towards sustainability. Heliyon *10*. https://doi.org/10.1016/j.heliyon.2023.e22361.

29. Villasante, S., Sumaila, U.R., Da-Rocha, J.M., Carvalho, N., Skerritt, D.J., Schuhbauer, A., Cisneros-Montemayor, A.M., Bennett, N.J., Hanich, Q., and Prellezo, R. (2022). Strengthening European Union fisheries by removing harmful subsidies. Marine Policy *136*, 104884. https://doi.org/10.1016/j.marpol.2021.104884.

30. Bisaro, A., and Meyer, K. (2022). Integrating Nature-based Solutions into policies for climate change adaptation and disaster risk reduction (IUCN).

31. Funder, M., and Gravesen, M.L. (2022). How to support a rights-based approach to nature-based solutions. (Danish Institute for International Studies).

32. Muthee, K., Duguma, L., Nzyoka, J., and Minang, P. (2021). Ecosystem-Based Adaptation Practices as a Nature-Based Solution to Promote Water-Energy-Food Nexus Balance. Sustainability *13*, 1142. https://doi.org/10.3390/su13031142.

33. Gevaña, D.T., Garcia, J.E., Ruzol, C.D., Malabayabas, F.L., Grefalda, L.B., O'Brien, E., Santos, E.P., and Camacho, L.D. (2021). Climate Change Resiliency Through Mangrove Conservation: The Case of Alitas Farmers of Infanta, Philippines. In Fostering Transformative Change for Sustainability in the Context of Socio-Ecological Production Landscapes and Seascapes (SEPLS), M. Nishi, S. M. Subramanian, H. Gupta, M. Yoshino, Y. Takahashi, K. Miwa, and T. Takeda, eds. (Springer), pp. 195–214. https://doi.org/10.1007/978-981-33-6761-6_11.

34. Lemieux, C.J., Kraus, D.T., and Beazley, K.F. (2022). Running to stand still: The application of substandard OECMs in national and provincial policy in Canada. Biological Conservation *275*. https://doi.org/10.1016/j.biocon.2022.109780.

35. Dalton, D.T., Berger, V., Adams, V., Botha, J., Halloy, S., Kirchmeir, H., Sovinc, A., Steinbauer, K., Švara, V., and Jungmeier, M. (2023). A Conceptual Framework for Biodiversity Monitoring Programs in Conservation Areas. Sustainability (Switzerland) *15*. https://doi.org/10.3390/su15086779.

36. Maini, B., Blythe, J.L., Darling, E.S., and Gurney, G.G. (2023). Charting the value and limits of other effective conservation measures (OECMs) for marine conservation: A Delphi study. Marine Policy *147*. https://doi.org/10.1016/j.marpol.2022.105350.

37. Dey, D., and Amerasinghe, P.H. (2022). Community "Bio-Rights" in Augmenting Health and Climate Resilience of a Socio-Ecological Production Landscape in Peri-urban Ramsar Wetlands. In Biodiversity-Health-Sustainability Nexus in Socio-Ecological Production Landscapes and Seascapes (SEPLS) Satoyama Initiative Thematic Review., M. Nishi, S. M. Subramanian, and H. Gupta, eds. (Springer Nature), pp. 107–127. https://doi.org/10.1007/978-981-16-9893-4_6.

38. Gilbert, J. (2018). Governance of Natural Resources and Human Rights: From Development to Benefit-Sharing. In Natural Resources and Human Rights: An Appraisal, J. Gilbert, ed. (Oxford University Press), p. 0. https://doi.org/10.1093/oso/9780198795667.003.0004.

39. Gupta, H., Nishi, M., and Gasparatos, A. (2021). Community-based responses for tackling environmental and socio-economic change and impacts in mountain social–ecological systems. Ambio. https://doi.org/10.1007/s13280-021-01651-6.

40. Springer, J., Campese, J., and Nakangu, B. (2021). The Natural Resource Governance Framework : improving governance for equitable and effective conservation (IUCN, International Union for Conservation of Nature) https://doi.org/10.2305/IUCN.CH.2021.16.en.

41. Gadgil, M. (2000). People's Biodiversity Registers: Lessons Learnt. Environment Development and Sustainability *2*, 323–332. https://doi.org/10.1023/A:1011438729881.

42. Alamgir, A.N.M. (2017). Intellectual property (IP) and intellectual property right (IPR), traditional knowledge (TK) and protection of traditional medical knowledge (TMK). Progress in Drug Research *73*, 515–528. https://doi.org/10.1007/978-3-319-63862-1_12.

43. Reyes-García, V., and Benyei, P. (2019). Indigenous knowledge for conservation. Nat Sustain *2*, 657–658. https://doi.org/10.1038/s41893-019-0341-z.

44. Hegde, N., and Joosten, H. (2023). The impact of traditional conservation practices on species composition and diversity patterns of sacred swamps in the central Western Ghats, India. Wetlands Ecology and Management *31*, 245–266. https://doi.org/10.1007/s11273-023-09914-7.

45. Htoo, M.H.C., Steenhuisen, B., and Verschuuren, B. (2023). Different Natures of Reality Inform Different Realities of Nature: What Karen Perceptions of Forest Reveal about Nature Conservation in Indigenous Contexts. Conservation and Society *21*, 28–37. https://doi.org/10.4103/cs.cs_83_21.

46. Melaku, A., Ivars, J.P., and Sahle, M. (2023). The state-of-the-art and future research directions on sacred forests and ecosystem services. Environmental Management *71*, 1255–1268. https://doi.org/10.1007/s00267-023-01790-4.

47. Mweta, N., Juma, P., and Phiri, S.B. (2023). Conservation of Natural Forests Through Indigenous Religious Beliefs and Practices: A Case Study of M'bona Cult, Nsanje District, Malawi. In Socio-Ecological Systems and Decoloniality: Convergence of Indigenous and Western Knowledge, pp. 157–177. https://doi.org/10.1007/978-3-031-15097-5_8.

48. Plieninger, T., Abunnasr, Y., D'Ambrosio, U., Guo, T., Kizos, T., Kmoch, L., Topp, E., and Varela, E. (2023). Biocultural conservation systems in the Mediterranean region: the role of values, rules, and knowledge. Sustainability Science *18*, 823–838. https://doi.org/10.1007/s11625-022-01155-6.

49. Nishi, M., Subramanian, S.M., Gupta, H., Yoshino, M., Takahashi, Y., Miwa, K., and Takeda, T. (2021). Synthesis: Conception, Approaches and Strategies for Transformative Change. In Fostering Transformative Change for Sustainability in the Context of Socio-Ecological Production Landscapes and Seascapes (SEPLS), M. Nishi, S. M. Subramanian, H. Gupta, M. Yoshino, Y. Takahashi, K. Miwa, and T. Takeda, eds. (Springer Singapore), pp. 229–249. https://doi.org/10.1007/978-981-33-6761-6_13.

50. Raharilaza, N.V. (2021). Degraded Landscape Transformed into Foodland and Woodland by Village Agroforestry. In Fostering Transformative Change for Sustainability in the Context of Socio-Ecological Production Landscapes and Seascapes (SEPLS), M. Nishi, S. M. Subramanian, H. Gupta, M. Yoshino, Y. Takahashi, K. Miwa, and T. Takeda, eds. (Springer), pp. 37–60. https://doi.org/10.1007/978-981-33-6761-6_3.

51. Saxena, K.G., Rao, K.S., and Maikhuri, R.K. (2021). Long-Term Tracking of Multiple Benefits of Participatory Forest Restoration in Marginal Cultural Landscapes in Himalaya. In Fostering Transformative Change for Sustainability in the Context of Socio-Ecological Production Landscapes and Seascapes (SEPLS), M. Nishi, S. M. Subramanian, H. Gupta, M. Yoshino, Y. Takahashi, K. Miwa, and T. Takeda, eds. (Springer), pp. 61–75. https://doi.org/10.1007/978-981-33-6761-6_4.

52. Franco, I.B., and Tracey, J. (2019). Community capacity-building for sustainable development: Effectively striving towards achieving local community sustainability targets. IJSHE *20*, 691–725. https://doi.org/10.1108/IJSHE-02-2019-0052.

53. Gualandi, G., and Williams-Gualandi, D. (2021). Transformative Change Through Ecological Consumption and Production of Ancient Wheat Varieties in Tuscany, Italy. In Fostering Transformative Change for Sustainability in the Context of Socio-Ecological Production Landscapes and Seascapes (SEPLS), M. Nishi, S. M. Subramanian, H. Gupta, M. Yoshino, Y. Takahashi, K. Miwa, and T. Takeda, eds. (Springer), pp. 95–114. https://doi.org/10.1007/978-981-33-6761-6_6.

54. Batavia, C., Nelson, M.P., and Wallach, A.D. (2020). The moral residue of conservation. Conservation Biology *34*, 1114–1121. https://doi.org/10.1111/cobi.13463.

55. Gosling, J., and Case, P. (2013). Social dreaming and ecocentric ethics: sources of non-rational insight in the face of climate change catastrophe. Organization *20*, 705–721. https://doi.org/10.1177/1350508413489814.

56. Katz, E. (1999). A Pragmatic Reconsideration of Anthropocentrism. Environmental Ethics *21*, 377–390. https://doi.org/10.5840/enviroethics19992144.

57. Lee, K.-H., Noh, J., and Khim, J.S. (2020). The Blue Economy and the United Nations' sustainable development goals: Challenges and opportunities. Environ Int *137*, 105528. https://doi.org/10.1016/j.envint.2020.105528.

58. Bennett, N.J., Blythe, J., White, C.S., and Campero, C. (2021). Blue growth and blue justice: Ten risks and solutions for the ocean economy. Marine Policy *125*, 104387. https://doi.org/10.1016/j.marpol.2020.104387.

59. Cohen, P.J., Allison, E.H., Andrew, N.L., Cinner, J., Evans, L.S., Fabinyi, M., Garces, L.R., Hall, S.J., Hicks, C.C., Hughes, T.P., et al. (2019). Securing a Just Space for Small-Scale Fisheries in the Blue Economy. Front. Mar. Sci. *6*. https://doi.org/10.3389/fmars.2019.00171.

60. Kortenkamp, K.V., and Moore, C.F. (2001). ECOCENTRISM AND ANTHROPOCENTRISM: MORAL REASONING ABOUT ECOLOGICAL COMMONS DILEMMAS. Journal of Environmental Psychology *21*, 261–272. https://doi.org/10.1006/jevp.2001.0205.

61. Carter, A. (2005). Inegalitarian Biocentric Consequentialism, the Minimax Implication and Multidimensional Value Theory: A Brief Proposal for a New Direction in Environmental Ethics. Utilitas *17*, 62–84. https://doi.org/10.1017/S0953820804001402.

62. Feenberg, A. (2003). Pragmatism and Critical Theory of Technology. Techné: Research in Philosophy and Technology *7*, 29–33. https://doi.org/10.5840/techne20037114.

63. Hällmark, K. (2023). Politicization after the 'end of nature': The prospect of ecomodernism. European Journal of Social Theory *26*, 48–66. https://doi.org/10.1177/13684310221103759.

64. Kriebel, D., Tickner, J., Epstein, P., Lemons, J., Levins, R., Loechler, E.L., Quinn, M., Rudel, R., Schettler, T., and Stoto, M. (2001). The precautionary principle in environmental science. Environ Health Perspect *109*, 871–876. https://doi.org/10.1289/ehp.01109871.

65. Möller, V., Diemen, R. van, Matthews, J.B.R., Méndez, C., Semenov, S., Fuglestvedt, J.S., and Reisinger, A. (2022). Annex II: Glossary.

66. Harden-Davies, H., Humphries, F., Maloney, M., Wright, G., Gjerde, K., and Vierros, M. (2020). Rights of Nature: Perspectives for Global Ocean Stewardship. Marine Policy *122*, 104059. https://doi.org/10.1016/j.marpol.2020.104059.

67. Veraart, R., Blok, V., and Lemmens, P. (2023). Ecomodernism and the Libidinal Economy: Towards a Critical Conception of Technology in the Bio-Based Economy. Philos. Technol. *36*, 18. https://doi.org/10.1007/s13347-023-00617-8.

68. Ellis, E.C., Lynas, M., and Nordhaus, T. (2024). Ecomodernism: A clarifying perspective. The Anthropocene Review *11*, 684–685. https://doi.org/10.1177/20530196231221495.

69. Arnold, R.D., and Wade, J.P. (2015). A Definition of Systems Thinking: A Systems Approach. Procedia Computer Science *44*, 669–678. https://doi.org/10.1016/j.procs.2015.03.050.

70. Cole, R.J. (2012). Regenerative design and development: current theory and practice. Building Research & Information *40*, 1–6. https://doi.org/10.1080/09613218.2012.617516.

71. Pedersen Zari, M. (2012). Ecosystem services analysis for the design of regenerative built environments. Building Research & Information *40*, 54–64. https://doi.org/10.1080/09613218.2011.628547.

72. Naboni, E., Natanian, J., Brizzi, G., Florio, P., Chokhachian, A., Galanos, T., and Rastogi, P. (2019). A digital workflow to quantify regenerative urban design in the context of a changing climate. Renewable and Sustainable Energy Reviews *113*, 109255. https://doi.org/10.1016/j.rser.2019.109255.

73. Dower, N. (2014). Global ethics: dimensions and prospects. Journal of Global Ethics *10*, 8–15. https://doi.org/10.1080/17449626.2014.896575.

74. Bauman, W.A. (2017). The Ethics of Wicked Problems. Worldviews *21*, 7–20. https://doi.org/10.1163/15685357-02101002.

75. Maffi, L. (2005). LINGUISTIC, CULTURAL, AND BIOLOGICAL DIVERSITY. Annu. Rev. Anthropol. *34*, 599–617. https://doi.org/10.1146/annurev.anthro.34.081804.120437.

76. Mang, P., and Reed, B. (2012). Designing from place: a regenerative framework and methodology. Building Research & Information *40*, 23–38. https://doi.org/10.1080/09613218.2012.621341.

77. Naess, A. (1973). The shallow and the deep, long-range ecology movement. A summary∗. Inquiry *16*, 95–100. https://doi.org/10.1080/00201747308601682.

78. Voyer, M., Quirk, G., McIlgorm, A., and Azmi, K. (2018). Shades of blue: what do competing interpretations of the Blue Economy mean for oceans governance? Preprint, https://doi.org/10.31230/osf.io/ksq6n https://doi.org/10.31230/osf.io/ksq6n.

79. Singh, P.K., and Chudasama, H. (2021). Pathways for climate resilient development: Human well-being within a safe and just space in the 21st century. Global Environmental Change *68*, 102277. https://doi.org/10.1016/j.gloenvcha.2021.102277.

80. Bharucha, Z.P., Mitjans, S.B., and Pretty, J. (2020). Towards redesign at scale through zero budget natural farming in Andhra Pradesh, India. International Journal of Agricultural Sustainability *18*, 1–20. https://doi.org/10.1080/14735903.2019.1694465.

81. Duddigan, S., Collins, C.D., Hussain, Z., Osbahr, H., Shaw, L.J., Sinclair, F., Sizmur, T., Thallam, V., and Ann Winowiecki, L. (2022). Impact of Zero Budget Natural Farming on Crop Yields in Andhra Pradesh, SE India. Sustainability *14*, 1689. https://doi.org/10.3390/su14031689.

82. Jaacks, L.M., Serupally, R., Dabholkar, S., Venkateshmurthy, N.S., Mohan, S., Roy, A., Prabhakaran, P., Smith, B., Gathorne-Hardy, A., Veluguri, D., et al. (2022). Impact of large-scale, government legislated and funded organic farming training on pesticide use in Andhra Pradesh, India: a cross-sectional study. The Lancet Planetary Health *6*, e310–e319. https://doi.org/10.1016/S2542-5196(22)00062-6.

83. Kumar, C.P. (2022). Sustainable Agriculture in India - Natural Farming Spreads Roots in Andhra Pradesh. ash *10*, 16–21. https://doi.org/10.34293/sijash.v10i2.5159.

84. Khadse, A., and Rosset, P.M. (2019). Zero Budget Natural Farming in India – from inception to institutionalization. Agroecology and Sustainable Food Systems *43*, 848–871. https://doi.org/10.1080/21683565.2019.1608349.

85. Duchelle, A.E., Cromberg, M., Gebara, M.F., Guerra, R., Melo, T., Larson, A., Cronkleton, P., Börner, J., Sills, E., Wunder, S., et al. (2014). Linking Forest Tenure Reform, Environmental Compliance, and Incentives: Lessons from REDD+ Initiatives in the Brazilian Amazon. World Development *55*, 53–67. https://doi.org/10.1016/j.worlddev.2013.01.014.

86. Garcia, B., Rimmer, L., Canal Vieira, L., and Mackey, B. (2021). REDD+ and forest protection on indigenous lands in the Amazon. Rev Euro Comp Intl Enviro *30*, 207–219. https://doi.org/10.1111/reel.12389.

87. Gebara, M., and Agrawal, A. (2017). Beyond Rewards and Punishments in the Brazilian Amazon: Practical Implications of the REDD+ Discourse. Forests *8*, 66. https://doi.org/10.3390/f8030066.

88. Shankland, A., and Hasenclever, L. (2011). Indigenous Peoples and the Regulation of REDD+ in Brazil: Beyond the War of the Worlds? IDS Bulletin *42*, 80–88. https://doi.org/10.1111/j.1759-5436.2011.00225.x.

89. West, T.A.P. (2016). Indigenous community benefits from a de-centralized approach to REDD+ in Brazil. Climate Policy *16*, 924–939. https://doi.org/10.1080/14693062.2015.1058238.

90. Blackman, A., and Veit, P. (2018). Titled Amazon Indigenous Communities Cut Forest Carbon Emissions. Ecological Economics *153*, 56–67. https://doi.org/10.1016/j.ecolecon.2018.06.016.

91. Wylie, L., Sutton-Grier, A.E., and Moore, A. (2016). Keys to successful blue carbon projects: Lessons learned from global case studies. Marine Policy *65*, 76–84. https://doi.org/10.1016/j.marpol.2015.12.020.

92. Huff, A., and Tonui, C. (2017). Making "Mangroves Together": Carbon, conservation and co-management in Gazi Bay, Kenya (STEPS Working Paper 95) (STEPS Centre, Institute of Development Studies).

93. Formuli, F., Mazza, F., Medeleanu, M., Sant'Ana, T.T., and Wong, J. (2023). A Virtuous Cycle: Mangrove Conservation and Blue Carbon Initiatives in Coastal Kenya (Reach Alliance).

94. Anguelovski, I., Irazábal-Zurita, C., and Connolly, J.J.T. (2019). Grabbed Urban Landscapes: Socio-spatial Tensions in Green Infrastructure Planning in Medellín. Int J Urban Regional Res *43*, 133–156. https://doi.org/10.1111/1468-2427.12725.

95. Garcia Ferrari, S., Smith, H., Coupe, F., and Rivera, H. (2018). City profile: Medellin. Cities *74*, 354–364. https://doi.org/10.1016/j.cities.2017.12.011.

96. Hammelman, C., and Saenz-Montoya, A. (2020). Territorializing the Urban-Rural Border in Medellín, Colombia: Socio-Ecological Assemblages and Disruptions. Journal of Latin American Geography *19*, 36–59. https://doi.org/10.1353/lag.2020.0031.

97. Humphrey, M., and Valverde, E. (2017). A Tale of Dual Cities: The Urban Miracle and Humanitarian Crisis Maps of Medellín (2002–2015). Journal of Iberian and Latin American Research *23*, 159–177. https://doi.org/10.1080/13260219.2017.1365927.

98. Reynolds, C., Escobedo, F., Clerici, N., and Zea-Camaño, J. (2017). Does "Greening" of Neotropical Cities Considerably Mitigate Carbon Dioxide Emissions? The Case of Medellin, Colombia. Sustainability *9*, 785. https://doi.org/10.3390/su9050785.

99. Sotomayor, L. (2017). Dealing with Dangerous Spaces: The Construction of Urban Policy in Medellín. Latin American Perspectives *44*, 71–90. https://doi.org/10.1177/0094582X16682758.

100. Anil Kumar, N., Sivan, V.V., and Vipindas, P. (2022). Promoting Local Health Traditions and Local Food Baskets: A Case Study from a Biocultural Hotspot of India. In Biodiversity-Health-Sustainability Nexus in Socio-Ecological Production Landscapes and Seascapes (SEPLS) Satoyama Initiative Thematic Review., M. Nishi, S. M. Subramanian, and H. Gupta, eds. (Springer Nature), pp. 177–198. https://doi.org/10.1007/978-981-16-9893-4_9.

101. Flachs, A., and Richards, P. (2018). Playing development roles: the political ecology of performance in agricultural development. Journal of Political Ecology *25*. https://doi.org/10.2458/v25i1.23089.

102. Chandirasekaran, V.M., and Rani, M.G. (2003). Conservation and characterization of traditional varieties of legumes and cucurbits in Wayanad. CROP DIVERSITY AND TRIBAL EMPOWERMENT, 187–190.

103. Kumar, N.A., Nambi, V.A., Rani, M.G., King, E.D.I.O., Chaudhury, S.S., and Mishra, S. (2015). Community agro biodiversity conservation continuum: an integrated approach to achieve food and nutrition security. Current Science *109*, 474–487.

104. Green Resources AS and Busoga Forestry Company Ltd. (2019). Project design document form: Kachung Forest Project: Afforestation on Degraded Lands (Version 09).

105. Busoga Forestry Company Ltd., Green Resources AS, and Swedish Energy Agency (2021). Monitoring report: Kachung Forest Project: Afforestation on Degraded Lands, UNFCCC project activity 4653 (Version 1; Third monitoring period, 23/11/2017–20/05/2021).

106. Hajdu, F., Penje, O., and Fischer, K. (2016). Questioning the use of 'degradation' in climate mitigation: A case study of a forest carbon CDM project in Uganda. Land Use Policy *59*, 412–422. https://doi.org/10.1016/j.landusepol.2016.09.016.

107. Lyons, K., and Westoby, P. (2014). Carbon colonialism and the new land grab: Plantation forestry in Uganda and its livelihood impacts. Journal of Rural Studies *36*, 13–21. https://doi.org/10.1016/j.jrurstud.2014.06.002.

108. Fischer, K., and Hajdu, F. (2018). The importance of the will to improve: how 'sustainability' sidelined local livelihoods in a carbon-forestry investment in Uganda. Journal of Environmental Policy & Planning *20*, 328–341. https://doi.org/10.1080/1523908X.2017.1410429.

109. Edstedt, K., and Carton, W. (2018). The benefits that (only) capital can see? Resource access and degradation in industrial carbon forestry, lessons from the CDM in Uganda. Geoforum *97*, 315–323. https://doi.org/10.1016/j.geoforum.2018.09.030.

110. Fischer, K., Giertta, F., and Hajdu, F. (2019). Carbon-binding biomass or a diversity of useful trees? (Counter)topographies of carbon forestry in Uganda. Environment and Planning E: Nature and Space *2*, 178–199. https://doi.org/10.1177/2514848618823598.

111. Smith, P. (2016). Soil carbon sequestration and biochar as negative emission technologies. Global Change Biology *22*, 1315–1324. https://doi.org/10.1111/gcb.13178.

112. FAO and ITPS (2015). Status of the World's Soil Resources: Main Report.

113. Smith, P., Soussana, J., Angers, D., Schipper, L., Chenu, C., Rasse, D.P., Batjes, N.H., Van Egmond, F., McNeill, S., Kuhnert, M., et al. (2020). How to measure, report and verify soil carbon change to realize the potential of soil carbon sequestration for atmospheric greenhouse gas removal. Global Change Biology *26*, 219–241. https://doi.org/10.1111/gcb.14815.

114. Jones, C., McConnell, C., Coleman, K., Cox, P., Falloon, P., Jenkinson, D., and Powlson, D. (2005). Global climate change and soil carbon stocks; predictions from two contrasting models for the turnover of organic carbon in soil. Global Change Biology *11*, 154–166. https://doi.org/10.1111/j.1365-2486.2004.00885.x.

115. Cheng, K., Xu, X., Cui, L., Li, Y., Zheng, J., Wu, W., Sun, J., and Pan, G. (2021). The role of soils in regulation of freshwater and coastal water quality. Phil. Trans. R. Soc. B *376*, 20200176. https://doi.org/10.1098/rstb.2020.0176.

116. Keesstra, S., Sannigrahi, S., López-Vicente, M., Pulido, M., Novara, A., Visser, S., and Kalantari, Z. (2021). The role of soils in regulation and provision of blue and green water. Phil. Trans. R. Soc. B *376*, 20200175. https://doi.org/10.1098/rstb.2020.0175.

117. Qiao, L., Wang, X., Smith, P., Fan, J., Lu, Y., Emmett, B., Li, R., Dorling, S., Chen, H., Liu, S., et al. (2022). Soil quality both increases crop production and improves resilience to climate change. Nat. Clim. Chang. *12*, 574–580. https://doi.org/10.1038/s41558-022-01376-8.

118. IPBES (2018). The IPBES assessment report on land degradation and restoration. (Secretariat of the Intergovernmental Science-Policy Platform on Biodiversity and Ecosystem Services) https://doi.org/10.5281/ZENODO.3237392.

119. Smith, P., Cotrufo, M.F., Rumpel, C., Paustian, K., Kuikman, P.J., Elliott, J.A., McDowell, R., Griffiths, R.I., Asakawa, S., Bustamante, M., et al. (2015). Biogeochemical cycles and biodiversity as key drivers of ecosystem services provided by soils. SOIL *1*, 665–685. https://doi.org/10.5194/soil-1-665-2015.

120. De Deyn, G.B., and Kooistra, L. (2021). The role of soils in habitat creation, maintenance and restoration. Phil. Trans. R. Soc. B *376*, 20200170. https://doi.org/10.1098/rstb.2020.0170.

121. Lal, R. (2015). Research and Development Priorities in Water Security. Agronomy Journal *107*, 1567–1572. https://doi.org/10.2134/agronj15.0046.

122. Smith, P., Calvin, K., Nkem, J., Campbell, D., Cherubini, F., Grassi, G., Korotkov, V., Le Hoang, A., Lwasa, S., McElwee, P., et al. (2020). Which practices co-deliver food security, climate change mitigation and adaptation, and combat land degradation and desertification? Global Change Biology *26*, 1532–1575. https://doi.org/10.1111/gcb.14878.

123. McElwee, P. (2021). The role of soils in learning and inspiration, physical and psychological experiences, and in supporting identities. Philosophical Transactions of the Royal Society B *376*, 20200184. https://doi.org/10.1098/rstb.2020.0184.

124. Ichikawa K. (2012). India: Homegardens in South India and the Andaman and Nicobar Islands. International Partnership for the Satoyama Initiative. https://satoyama-initiative.org/case_studies/india-homegardens-in-south-india-and-the-andaman-and-nicobar-islands/.

125. Castañeda-Álvarez, N.P., Khoury, C.K., Achicanoy, H.A., Bernau, V., Dempewolf, H., Eastwood, R.J., Guarino, L., Harker, R.H., Jarvis, A., Maxted, N., et al. (2016). Global conservation priorities for crop wild relatives. Nature Plants *2*, 16022. https://doi.org/10.1038/nplants.2016.22.

126. Garnett, T., Appleby, M.C., Balmford, A., Bateman, I.J., Benton, T.G., Bloomer, P., Burlingame, B., Dawkins, M., Dolan, L., Fraser, D., et al. (2013). Sustainable Intensification in Agriculture: Premises and Policies. Science *341*, 33–34. https://doi.org/10.1126/science.1234485.

127. Cassman, K.G., and Grassini, P. (2020). A global perspective on sustainable intensification research. Nat Sustain *3*, 262–268. https://doi.org/10.1038/s41893-020-0507-8.

128. Pretty, J., Benton, T.G., Bharucha, Z.P., Dicks, L.V., Flora, C.B., Godfray, H.C.J., Goulson, D., Hartley, S., Lampkin, N., Morris, C., et al. (2018). Global assessment of agricultural system redesign for sustainable intensification. Nat Sustain *1*, 441–446. https://doi.org/10.1038/s41893-018-0114-0.

129. FAO (2022). The State of the World's Land and Water Resources for Food and Agriculture 2021 – Systems at breaking point (FAO) https://doi.org/10.4060/cb9910en.

130. Corbeels, M., Cardinael, R., Powlson, D., Chikowo, R., and Gerard, B. (2020). Carbon sequestration potential through conservation agriculture in Africa has been largely overestimated. Soil and Tillage Research *196*, 104300. https://doi.org/10.1016/j.still.2019.104300.

131. Haughey, E., Neogi, S., Portugal-Pereira, J., Van Diemen, R., and Slade, R.B. (2023). Sustainable intensification and carbon sequestration research in agricultural systems: A systematic review. Environmental Science & Policy *143*, 14–23. https://doi.org/10.1016/j.envsci.2023.02.018.

132. Brauman, K.A., Siebert, S., and Foley, J.A. (2013). Improvements in crop water productivity increase water sustainability and food security—a global analysis. Environ. Res. Lett. *8*, 024030. https://doi.org/10.1088/1748-9326/8/2/024030.

133. Bais-Moleman, A.L., Schulp, C.J.E., and Verburg, P.H. (2019). Assessing the environmental impacts of production- and consumption-side measures in sustainable agriculture intensification in the European Union. Geoderma *338*, 555–567. https://doi.org/10.1016/j.geoderma.2018.11.042.

134. Ngoma, H., Simutowe, E., Manyanga, M., and Thierfelder, C. (2023). Sustainable intensification and household dietary diversity in maize-based farming systems of Zambia and Zimbabwe. Outlook Agric *52*, 34–46. https://doi.org/10.1177/00307270221150660.

135. Wilkus, E.L., deVoil, P., Marenya, P., Snapp, S., Dixon, J., and Rodriguez, D. (2022). Sustainable Intensification Practices Reduce Food Deficit for the Best- and Worst-Off Households in Ethiopia and Mozambique. Front. Sustain. Food Syst. *5*, 649218. https://doi.org/10.3389/fsufs.2021.649218.

136. Godfray, H.C.J. (2015). The debate over sustainable intensification. Food Sec. *7*, 199–208. https://doi.org/10.1007/s12571-015-0424-2.

137. Rockström, J., Williams, J., Daily, G., Noble, A., Matthews, N., Gordon, L., Wetterstrand, H., DeClerck, F., Shah, M., Steduto, P., et al. (2017). Sustainable intensification of agriculture for human prosperity and global sustainability. Ambio *46*, 4–17. https://doi.org/10.1007/s13280-016-0793-6.

138. Garrett, R.D., Koh, I., Lambin, E.F., Le Polain De Waroux, Y., Kastens, J.H., and Brown, J.C. (2018). Intensification in agriculture-forest frontiers: Land use responses to development and conservation policies in Brazil. Global Environmental Change *53*, 233–243. https://doi.org/10.1016/j.gloenvcha.2018.09.011.

139. Le Polain De Waroux, Y., Garrett, R.D., Graesser, J., Nolte, C., White, C., and Lambin, E.F. (2019). The Restructuring of South American Soy and Beef Production and Trade Under Changing Environmental Regulations. World Development *121*, 188–202. https://doi.org/10.1016/j.worlddev.2017.05.034.

140. Piemontese, L., Kamugisha, R.N., Barron, J., Tukahirwa, J.M.B., Harari, N., and Jaramillo, F. (2022). Investing in sustainable intensification for smallholders: quantifying large-scale costs and benefits in Uganda. Environ. Res. Lett. *17*, 045010. https://doi.org/10.1088/1748-9326/ac5ae0.

141. Kassie, M., Teklewold, H., Jaleta, M., Marenya, P., and Erenstein, O. (2015). Understanding the adoption of a portfolio of sustainable intensification practices in eastern and southern Africa. Land Use Policy *42*, 400–411. https://doi.org/10.1016/j.landusepol.2014.08.016.

142. Chopin, T., Cooper, J.A., Reid, G., Cross, S., and Moore, C. (2012). Open-water integrated multi-trophic aquaculture: environmental biomitigation and economic diversification of fed aquaculture by extractive aquaculture. Reviews in Aquaculture *4*, 209–220. https://doi.org/10.1111/j.1753-5131.2012.01074.x.

143. FAO (2022). Integrated multitrophic aquaculture: Lessons from China.

144. UNEP (2023). Seaweed farming: Assessment on the Potential of Sustainable Upscaling for Climate, Communities and the Planet. (United Nations Environment Programme).

145. Hamilton, S.L., Elliott, M.S., deVries, M.S., Adelaars, J., Rintoul, M.D., and Graham, M.H. (2022). Integrated multi-trophic aquaculture mitigates the effects of ocean acidification: Seaweeds raise system pH and improve growth of juvenile abalone. Aquaculture *560*, 738571. https://doi.org/10.1016/j.aquaculture.2022.738571.

146. Hori, M., Hamaguchi, M., Sato, M., Tremblay, R., Correia-Martins, A., Derolez, V., Richard, M., and LAGARD, F. (2021). Oyster aquaculture using seagrass beds as a climate change countermeasure. 水産研究・教育機構研究報告 *50*, 123–133.

147. Kim, J.K., Yarish, C., Hwang, E.K., Park, M., and Kim, Y. (2017). Seaweed aquaculture: cultivation technologies, challenges and its ecosystem services. ALGAE *32*, 1–13. https://doi.org/10.4490/algae.2017.32.3.3.

148. Gentry, R.R., Alleway, H.K., Bishop, M.J., Gillies, C.L., Waters, T., and Jones, R. (2020). Exploring the potential for marine aquaculture to contribute to ecosystem services. Reviews in Aquaculture *12*, 499–512. https://doi.org/10.1111/raq.12328.

149. Stentiford, G.D., Bateman, I.J., Hinchliffe, S.J., Bass, D., Hartnell, R., Santos, E.M., Devlin, M.J., Feist, S.W., Taylor, N.G.H., Verner-Jeffreys, D.W., et al. (2020). Sustainable aquaculture through the One Health lens. Nat Food *1*, 468–474. https://doi.org/10.1038/s43016-020-0127-5.

150. Turetsky, M.R., Benscoter, B., Page, S., Rein, G., Van Der Werf, G.R., and Watts, A. (2015). Global vulnerability of peatlands to fire and carbon loss. Nature Geosci *8*, 11–14. https://doi.org/10.1038/ngeo2325.

151. UNEP (2022). Global Peatlands Assessment – The State of the World's Peatlands: Evidence for action toward the conservation, restoration, and sustainable management of peatlands. Global Peatlands Initiative (United Nations Environment Programme).

152. Smith, P., Arneth, A., Barnes, D.K.A., Ichii, K., Marquet, P.A., Popp, A., Pörtner, H., Rogers, A.D., Scholes, R.J., Strassburg, B., et al. (2022). How do we best synergize climate mitigation actions to co-benefit biodiversity? Global Change Biology *28*, 2555–2577. https://doi.org/10.1111/gcb.16056.

153. Munang, R., Thiaw, I., Alverson, K., Liu, J., and Han, Z. (2013). The role of ecosystem services in climate change adaptation and disaster risk reduction. Current Opinion in Environmental Sustainability *5*, 47–52. https://doi.org/10.1016/j.cosust.2013.02.002.

154. Miettinen, J., Shi, C., and Liew, S.C. (2016). Land cover distribution in the peatlands of Peninsular Malaysia, Sumatra and Borneo in 2015 with changes since 1990. Global Ecology and Conservation *6*, 67–78. https://doi.org/10.1016/j.gecco.2016.02.004.

155. Humpenöder, F., Karstens, K., Lotze-Campen, H., Leifeld, J., Menichetti, L., Barthelmes, A., and Popp, A. (2020). Peatland protection and restoration are key for climate change mitigation. Environ. Res. Lett. *15*, 104093. https://doi.org/10.1088/1748-9326/abae2a.

156. Dale, P.E.R., and Connelly, R. (2012). Wetlands and human health: an overview. Wetlands Ecol Manage *20*, 165–171. https://doi.org/10.1007/s11273-012-9264-4.

157. Griscom, B.W., Adams, J., Ellis, P.W., Houghton, R.A., Lomax, G., Miteva, D.A., Schlesinger, W.H., Shoch, D., Siikamäki, J.V., Smith, P., et al. (2017). Natural climate solutions. Proc. Natl. Acad. Sci. U.S.A. *114*, 11645–11650. https://doi.org/10.1073/pnas.1710465114.

158. Sadeghi, M., Chaston, T., Hanigan, I., De Dear, R., Santamouris, M., Jalaludin, B., and Morgan, G.G. (2022). The health benefits of greening strategies to cool urban environments – A heat health impact method. Building and Environment *207*, 108546. https://doi.org/10.1016/j.buildenv.2021.108546.

159. Smith, P., Adams, J., Beerling, D.J., Beringer, T., Calvin, K.V., Fuss, S., Griscom, B., Hagemann, N., Kammann, C., Kraxner, F., et al. (2019). Land-Management Options for Greenhouse Gas Removal and Their Impacts on Ecosystem Services and the Sustainable Development Goals. Annu. Rev. Environ. Resour. *44*, 255–286. https://doi.org/10.1146/annurev-environ-101718-033129.

160. Babiker, M., Berndes, G., Blok, K., Cohen, B., Cowie, A., Geden, O., Ginzburg, V., Leip, A., Smith, P., Sugiyama, M., et al. (2022). Cross-sectoral perspectives. In Climate Change 2022: Mitigation of Climate Change. Contribution of Working Group III to the Sixth Assessment Report of the Intergovernmental Panel on Climate Change, P. R. Shukla, J. Skea, R. Slade, R. Slade, A. A. Khourdajie, R. van Diemen, D. McCollum, M. Pathak, S. Some, P. Vyas, et al., eds. (Cambridge University Press).

161. Tan, Z.D., Sutikno, S., Carrasco, L.R., and Taylor, D. (2023). Local community representations of tropical peatlands and implications for restoration in Riau, Indonesia. Restoration Ecology *31*, e13900. https://doi.org/10.1111/rec.13900.

162. Barthelmie, R.J., and Pryor, S.C. (2021). Climate Change Mitigation Potential of Wind Energy. Climate *9*, 136. https://doi.org/10.3390/cli9090136.

163. Pek, A. (2020). Global Offshore Wind Report 2020 (Global Wind Energy Council).

164. Dorrell, R.M., Lloyd, C.J., Lincoln, B.J., Rippeth, T.P., Taylor, J.R., Caulfield, C.P., Sharples, J., Polton, J.A., Scannell, B.D., Greaves, D.M., et al. (2022). Anthropogenic Mixing in Seasonally Stratified Shelf Seas by Offshore Wind Farm Infrastructure. Front. Mar. Sci. *9*, 830927. https://doi.org/10.3389/fmars.2022.830927.

165. Dannheim, J., Bergström, L., Birchenough, S.N.R., Brzana, R., Boon, A.R., Coolen, J.W.P., Dauvin, J.-C., De Mesel, I., Derweduwen, J., Gill, A.B., et al. (2020). Benthic effects of offshore renewables: identification of knowledge gaps and urgently needed research. ICES Journal of Marine Science *77*, 1092–1108. https://doi.org/10.1093/icesjms/fsz018.

166. Galparsoro, I., Menchaca, I., Garmendia, J.M., Borja, Á., Maldonado, A.D., Iglesias, G., and Bald, J. (2022). Reviewing the ecological impacts of offshore wind farms. npj Ocean Sustain *1*, 1. https://doi.org/10.1038/s44183-022-00003-5.

167. Lloret, J., Turiel, A., Solé, J., Berdalet, E., Sabatés, A., Olivares, A., Gili, J.-M., Vila-Subirós, J., and Sardá, R. (2022). Unravelling the ecological impacts of large-scale offshore wind farms in the Mediterranean Sea. Science of The Total Environment *824*, 153803. https://doi.org/10.1016/j.scitotenv.2022.153803.

168. Saidur, R., Rahim, N.A., Islam, M.R., and Solangi, K.H. (2011). Environmental impact of wind energy. Renewable and Sustainable Energy Reviews *15*, 2423–2430. https://doi.org/10.1016/j.rser.2011.02.024.

169. Vaissière, A.-C., Levrel, H., Pioch, S., and Carlier, A. (2014). Biodiversity offsets for offshore wind farm projects: The current situation in Europe. Marine Policy *48*, 172–183. https://doi.org/10.1016/j.marpol.2014.03.023.

170. Ashley, M.C., Mangi, S.C., and Rodwell, L.D. (2014). The potential of offshore windfarms to act as marine protected areas – A systematic review of current evidence. Marine Policy *45*, 301–309. https://doi.org/10.1016/j.marpol.2013.09.002.

171. Boutin, K., Gaudron, S.M., Denis, J., and Ben Rais Lasram, F. (2023). Potential marine benthic colonisers of offshore wind farms in the English channel: A functional trait-based approach. Marine Environmental Research *190*, 106061. https://doi.org/10.1016/j.marenvres.2023.106061.

172. Degraer, S., Carey, D., Coolen, J., Hutchison, Z., Kerckhof, F., Rumes, B., and Vanaverbeke, J. (2020). Offshore Wind Farm Artificial Reefs Affect Ecosystem Structure and Functioning: A Synthesis. Oceanog *33*, 48–57. https://doi.org/10.5670/oceanog.2020.405.

173. Hammar, L., Perry, D., and Gullström, M. (2016). Offshore Wind Power for Marine Conservation. OJMS *06*, 66–78. https://doi.org/10.4236/ojms.2016.61007.

174. Mavraki, N., Degraer, S., Vanaverbeke, J., and Braeckman, U. (2020). Organic matter assimilation by hard substrate fauna in an offshore wind farm area: a pulse-chase study. ICES Journal of Marine Science *77*, 2681–2693. https://doi.org/10.1093/icesjms/fsaa133.

175. Banach, J.L., Van Den Burg, S.W.K., and Van Der Fels-Klerx, H.J. (2020). Food safety during seaweed cultivation at offshore wind farms: An exploratory study in the North Sea. Marine Policy *120*, 104082. https://doi.org/10.1016/j.marpol.2020.104082.

176. Stelzenmüller, V., Letschert, J., Gimpel, A., Kraan, C., Probst, W.N., Degraer, S., and Döring, R. (2022). From plate to plug: The impact of offshore renewables on European fisheries and the role of marine spatial planning. Renewable and Sustainable Energy Reviews *158*, 112108. https://doi.org/10.1016/j.rser.2022.112108.

177. Wiser, R., Rand, J., Seel, J., Beiter, P., Baker, E., Lantz, E., and Gilman, P. (2021). Expert elicitation survey predicts 37% to 49% declines in wind energy costs by 2050. Nat Energy *6*, 555–565. https://doi.org/10.1038/s41560-021-00810-z.

178. Valero, A., Valero, A., Calvo, G., and Ortego, A. (2018). Material bottlenecks in the future development of green technologies. Renewable and Sustainable Energy Reviews *93*, 178–200. https://doi.org/10.1016/j.rser.2018.05.041.

179. Levin, L.A., Amon, D.J., and Lily, H. (2020). Challenges to the sustainability of deep-seabed mining. Nat Sustain *3*, 784–794. https://doi.org/10.1038/s41893-020-0558-x.

180. Clarke, L., Wei, Y.-M., Navarro, A.D.L.V., Garg, A., Hahmann, A.N., Khennas, S., Azevedo, I.M.L., Löschel, A., Singh, A.K., Steg, L., et al. (2022). Energy Systems. In Climate Change 2022: Mitigation of Climate Change. Contribution of Working Group III to the Sixth Assessment Report of the Intergovernmental Panel on Climate Change, P. R. Shukla, J. Skea, R. Slade, R. Slade, A. A. Khourdajie, R. van Diemen, D. McCollum, M. Pathak, S. Some, P. Vyas, et al., eds. (Cambridge University Press).

181. Leirpoll, M.E., Næss, J.S., Cavalett, O., Dorber, M., Hu, X., and Cherubini, F. (2021). Optimal combination of bioenergy and solar photovoltaic for renewable energy production on abandoned cropland. Renewable Energy *168*, 45–56. https://doi.org/10.1016/j.renene.2020.11.159.

182. Schipper, E.L.F., A. Revi, B.L. Preston, E.R. Carr, S.H. Eriksen, L.R. Fernandez-Carril, B.C. Glavovic, N.J.M. Hilmi, D. Ley, R. Mukerji, et al. (2022). Climate Resilient Development Pathways. In Climate Change 2022: Impacts, Adaptation and Vulnerability. Contribution of Working Group II to the Sixth Assessment Report of the Intergovernmental Panel on Climate Change, H.-O. Pörtner, D.C. Roberts, M. Tignor, E.S. Poloczanska, K. Mintenbeck, A. Alegría, M. Craig, S. Langsdorf, S. Löschke, V. Möller, et al., eds. (Cambridge University Press), pp. 2655–2807.

183. Barron-Gafford, G.A., Pavao-Zuckerman, M.A., Minor, R.L., Sutter, L.F., Barnett-Moreno, I., Blackett, D.T., Thompson, M., Dimond, K., Gerlak, A.K., Nabhan, G.P., et al. (2019). Agrivoltaics provide mutual benefits across the food–energy–water nexus in drylands. Nat Sustain *2*, 848–855. https://doi.org/10.1038/s41893-019-0364-5.

184. Long, J., Lu, Z., Miller, P.A., Pongratz, J., Guan, D., Smith, B., Zhu, Z., Xu, J., and Zhang, Q. (2024). Large-scale photovoltaic solar farms in the Sahara affect solar power generation potential globally. Commun Earth Environ *5*, 11. https://doi.org/10.1038/s43247-023-01117-5.

185. Lu, Z., Zhang, Q., Miller, P.A., Zhang, Q., Berntell, E., and Smith, B. (2021). Impacts of Large-Scale Sahara Solar Farms on Global Climate and Vegetation Cover. Geophysical Research Letters *48*, e2020GL090789. https://doi.org/10.1029/2020GL090789.

186. Cousse, J. (2021). Still in love with solar energy? Installation size, affect, and the social acceptance of renewable energy technologies. Renewable and Sustainable Energy Reviews *145*, 111107. https://doi.org/10.1016/j.rser.2021.111107.

187. Patel, S., and Rao, K.V.S. (2016). Social acceptance of solar energy technology in India. In 2016 International Conference on Energy Efficient Technologies for Sustainability (ICEETS) (IEEE), pp. 142–147. https://doi.org/10.1109/ICEETS.2016.7582914.

188. O'Shaughnessy, E. (2022). Rooftop solar incentives remain effective for low- and moderate-income adoption. Energy Policy *163*, 112881. https://doi.org/10.1016/j.enpol.2022.112881.

189. O'Shaughnessy, K.A., Hawkins, S.J., Evans, A.J., Hanley, M.E., Lunt, P., Thompson, R.C., Francis, R.A., Hoggart, S.P.G., Moore, P.J., Iglesias, G., et al. (2020). Design catalogue for eco-engineering of coastal artificial structures: a multifunctional approach for stakeholders and end-users. Urban Ecosyst *23*, 431–443. https://doi.org/10.1007/s11252-019-00924-z.

190. Borenstein, S. (2017). Private Net Benefits of Residential Solar PV: The Role of Electricity Tariffs, Tax Incentives, and Rebates. Journal of the Association of Environmental and Resource Economists *4*, S85–S122. https://doi.org/10.1086/691978.

191. Dholakia, H.H. (2018). Solar powered healthcare in developing countries. Nature Energy *3*, 705–707.

192. Moner-Girona, M., Kakoulaki, G., Falchetta, G., Weiss, D.J., and Taylor, N. (2021). Achieving universal electrification of rural healthcare facilities in sub-Saharan Africa with decentralized renewable energy technologies. Joule *5*, 2687–2714. https://doi.org/10.1016/j.joule.2021.09.010.

193. Li, Y., Kalnay, E., Motesharrei, S., Rivas, J., Kucharski, F., Kirk-Davidoff, D., Bach, E., and Zeng, N. (2018). Climate model shows large-scale wind and solar farms in the Sahara increase rain and vegetation. Science *361*, 1019–1022. https://doi.org/10.1126/science.aar5629.

194. Bennun, L., Van Bochove, J., Ng, C., Fletcher, C., Wilson, D., Phair, N., and Carbone, G. (2021). Mitigating biodiversity impacts associated with solar and wind energy development: guidelines for project developers (IUCN, International Union for Conservation of Nature) https://doi.org/10.2305/IUCN.CH.2021.04.en.

195. Evans, M.J., Mainali, K., Soobitsky, R., Mills, E., and Minnemeyer, S. (2023). Predicting patterns of solar energy buildout to identify opportunities for biodiversity conservation. Biological Conservation *283*, 110074. https://doi.org/10.1016/j.biocon.2023.110074.

196. He, X., Feng, K., Li, X., Craft, A.B., Wada, Y., Burek, P., Wood, E.F., and Sheffield, J. (2019). Solar and wind energy enhances drought resilience and groundwater sustainability. Nat Commun *10*, 4893. https://doi.org/10.1038/s41467-019-12810-5.

197. Macknick, J., Newmark, R., Heath, G., and Hallett, K.C. (2012). Operational water consumption and withdrawal factors for electricity generating technologies: a review of existing literature. Environ. Res. Lett. *7*, 045802. https://doi.org/10.1088/1748-9326/7/4/045802.

198. Dhar, A., Naeth, M.A., Jennings, P.D., and Gamal El-Din, M. (2020). Perspectives on environmental impacts and a land reclamation strategy for solar and wind energy systems. Science of The Total Environment *718*, 134602. https://doi.org/10.1016/j.scitotenv.2019.134602.

199. Tian, X., Stranks, S.D., and You, F. (2021). Life cycle assessment of recycling strategies for perovskite photovoltaic modules. Nat Sustain *4*, 821–829. https://doi.org/10.1038/s41893-021-00737-z.

200. Yang, M., Chen, L., Wang, J., Msigwa, G., Osman, A.I., Fawzy, S., Rooney, D.W., and Yap, P.-S. (2023). Circular economy strategies for combating climate change and other environmental issues. Environ Chem Lett *21*, 55–80. https://doi.org/10.1007/s10311-022-01499-6.

201. Venkatesh, G. (2022). Circular Bio-economy—Paradigm for the Future: Systematic Review of Scientific Journal Publications from 2015 to 2021. Circ.Econ.Sust. *2*, 231–279. https://doi.org/10.1007/s43615-021-00084-3.

202. Velasco-Muñoz, J.F., Mendoza, J.M.F., Aznar-Sánchez, J.A., and Gallego-Schmid, A. (2021). Circular economy implementation in the agricultural sector: Definition, strategies and indicators. Resources, Conservation and Recycling *170*, 105618. https://doi.org/10.1016/j.resconrec.2021.105618.

203. Trigo, E., Chavarria, H., Pray, C., Smyth, S.J., Torroba, A., Wesseler, J., Zilberman, D., and Martinez, J.F. (2023). The Bioeconomy and Food System Transformation. In Science and Innovations for Food Systems Transformation, J. Von Braun, K. Afsana, L. O. Fresco, and M. H. A. Hassan, eds. (Springer International Publishing), pp. 849–868. https://doi.org/10.1007/978-3-031-15703-5_45.

204. Tait, J., Raybould, A., Flight, M.H., and McGoohan, A. (2023). Circular and Networked Bioeconomies for Net-Zero Food Production: There is Nothing Magic About Circles. Circ.Econ.Sust. *3*, 1907–1918. https://doi.org/10.1007/s43615-022-00247-w.

205. Stephenson, P.J., and Damerell, A. (2022). Bioeconomy and Circular Economy Approaches Need to Enhance the Focus on Biodiversity to Achieve Sustainability. Sustainability *14*, 10643. https://doi.org/10.3390/su141710643.

206. Sharma, P., Gaur, V.K., Sirohi, R., Varjani, S., Hyoun Kim, S., and Wong, J.W.C. (2021). Sustainable processing of food waste for production of bio-based products for circular bioeconomy. Bioresource Technology *325*, 124684. https://doi.org/10.1016/j.biortech.2021.124684.

207. Schneider, P., Rochell, V., Plat, K., and Jaworski, A. (2021). Circular Approaches in Small-Scale Food Production. Circ.Econ.Sust. *1*, 1231–1255. https://doi.org/10.1007/s43615-021-00129-7.

208. Sauvé, S., Lamontagne, S., Dupras, J., and Stahel, W. (2021). Circular economy of water: Tackling quantity, quality and footprint of water. Environmental Development *39*, 100651. https://doi.org/10.1016/j.envdev.2021.100651.

209. Rosenboom, J.-G., Langer, R., and Traverso, G. (2022). Bioplastics for a circular economy. Nat Rev Mater *7*, 117–137. https://doi.org/10.1038/s41578-021-00407-8.

210. Rodríguez-Espinosa, T., Papamichael, I., Voukkali, I., Gimeno, A.P., Candel, M.B.A., Navarro-Pedreño, J., Zorpas, A.A., and Lucas, I.G. (2023). Nitrogen management in farming systems under the use of agricultural wastes and circular economy. Science of The Total Environment *876*, 162666. https://doi.org/10.1016/j.scitotenv.2023.162666.

211. Qtaishat, Y., Hofman, J., and Adeyeye, K. (2022). Circular Water Economy in the EU: Findings from Demonstrator Projects. Clean Technol. *4*, 865–892. https://doi.org/10.3390/cleantechnol4030054.

212. Muhie, S.H. (2022). Novel approaches and practices to sustainable agriculture. Journal of Agriculture and Food Research *10*, 100446. https://doi.org/10.1016/j.jafr.2022.100446.

213. Morseletto, P., Mooren, C.E., and Munaretto, S. (2022). Circular Economy of Water: Definition, Strategies and Challenges. Circ.Econ.Sust. *2*, 1463–1477. https://doi.org/10.1007/s43615-022-00165-x.

214. Mak, T.M.W., Xiong, X., Tsang, D.C.W., Yu, I.K.M., and Poon, C.S. (2020). Sustainable food waste management towards circular bioeconomy: Policy review, limitations and opportunities. Bioresource Technology *297*, 122497. https://doi.org/10.1016/j.biortech.2019.122497.

215. Leong, H.Y., Chang, C.-K., Khoo, K.S., Chew, K.W., Chia, S.R., Lim, J.W., Chang, J.-S., and Show, P.L. (2021). Waste biorefinery towards a sustainable circular bioeconomy: a solution to global issues. Biotechnol Biofuels *14*, 87. https://doi.org/10.1186/s13068-021-01939-5.

216. Holden, N.M., Neill, A.M., Stout, J.C., O'Brien, D., and Morris, M.A. (2023). Biocircularity: a Framework to Define Sustainable, Circular Bioeconomy. Circ.Econ.Sust. *3*, 77–91. https://doi.org/10.1007/s43615-022-00180-y.

217. D'Amato, D., and Korhonen, J. (2021). Integrating the green economy, circular economy and bioeconomy in a strategic sustainability framework. Ecological Economics *188*, 107143. https://doi.org/10.1016/j.ecolecon.2021.107143.

218. Atiwesh, G., Mikhael, A., Parrish, C.C., Banoub, J., and Le, T.-A.T. (2021). Environmental impact of bioplastic use: A review. Heliyon *7*, e07918. https://doi.org/10.1016/j.heliyon.2021.e07918.

219. Abe, M.M., Martins, J.R., Sanvezzo, P.B., Macedo, J.V., Branciforti, M.C., Halley, P., Botaro, V.R., and Brienzo, M. (2021). Advantages and Disadvantages of Bioplastics Production from Starch and Lignocellulosic Components. Polymers *13*, 2484. https://doi.org/10.3390/polym13152484.

220. IPCC (2021). Climate Change 2021 – The Physical Science Basis: Working Group I Contribution to the Sixth Assessment Report of the Intergovernmental Panel on Climate Change 1st ed. (Cambridge University Press) https://doi.org/10.1017/9781009157896.

221. Bellouin, N., Baker, L., Hodnebrog, Ø., Olivié, D., Cherian, R., Macintosh, C., Samset, B., Esteve, A., Aamaas, B., Quaas, J., et al. (2016). Regional and seasonal radiative forcing by perturbations to aerosol and ozone precursor emissions. Atmos. Chem. Phys. *16*, 13885–13910. https://doi.org/10.5194/acp-16-13885-2016.

222. Bony, S., Stevens, B., Frierson, D.M.W., Jakob, C., Kageyama, M., Pincus, R., Shepherd, T.G., Sherwood, S.C., Siebesma, A.P., Sobel, A.H., et al. (2015). Clouds, circulation and climate sensitivity. Nature Geosci *8*, 261–268. https://doi.org/10.1038/ngeo2398.

223. Silva, R.A., Adelman, Z., Fry, M.M., and West, J.J. (2016). The Impact of Individual Anthropogenic Emissions Sectors on the Global Burden of Human Mortality due to Ambient Air Pollution. Environ Health Perspect *124*, 1776–1784. https://doi.org/10.1289/EHP177.

224. Stohl, A., Aamaas, B., Amann, M., Baker, L.H., Bellouin, N., Berntsen, T.K., Boucher, O., Cherian, R., Collins, W., Daskalakis, N., et al. (2015). Evaluating the climate and air quality impacts of short-lived pollutants. Atmos. Chem. Phys. *15*, 10529–10566. https://doi.org/10.5194/acp-15-10529-2015.

225. Xie, Y., Dai, H., Xu, X., Fujimori, S., Hasegawa, T., Yi, K., Masui, T., and Kurata, G. (2018). Co-benefits of climate mitigation on air quality and human health in Asian countries. Environment International *119*, 309–318. https://doi.org/10.1016/j.envint.2018.07.008.

226. Unger, N., Zheng, Y., Yue, X., and Harper, K.L. (2020). Mitigation of ozone damage to the world's land ecosystems by source sector. Nat. Clim. Chang. *10*, 134–137. https://doi.org/10.1038/s41558-019-0678-3.

227. Lee, D.S., Fahey, D.W., Skowron, A., Allen, M.R., Burkhardt, U., Chen, Q., Doherty, S.J., Freeman, S., Forster, P.M., Fuglestvedt, J., et al. (2021). The contribution of global aviation to anthropogenic climate forcing for 2000 to 2018. Atmospheric Environment *244*, 117834. https://doi.org/10.1016/j.atmosenv.2020.117834.

228. Jonson, J.E., Gauss, M., Schulz, M., Jalkanen, J.-P., and Fagerli, H. (2020). Effects of global ship emissions on European air pollution levels. Atmos. Chem. Phys. *20*, 11399–11422. https://doi.org/10.5194/acp-20-11399-2020.

229. Liu, H., Fu, M., Jin, X., Shang, Y., Shindell, D., Faluvegi, G., Shindell, C., and He, K. (2016). Health and climate impacts of ocean-going vessels in East Asia. Nature Clim Change *6*, 1037–1041. https://doi.org/10.1038/nclimate3083.

230. Benmarhnia, T., Rey, L., Cartier, Y., Clary, C.M., Deguen, S., and Brousselle, A. (2014). Addressing equity in interventions to reduce air pollution in urban areas: a systematic review. Int J Public Health *59*, 933–944. https://doi.org/10.1007/s00038-014-0608-0.

231. Thompson, T.M., Rausch, S., Saari, R.K., and Selin, N.E. (2016). Air quality co-benefits of subnational carbon policies. Journal of the Air & Waste Management Association *66*, 988–1002. https://doi.org/10.1080/10962247.2016.1192071.

232. Vandyck, T., Keramidas, K., Kitous, A., Spadaro, J.V., Van Dingenen, R., Holland, M., and Saveyn, B. (2018). Air quality co-benefits for human health and agriculture counterbalance costs to meet Paris Agreement pledges. Nat Commun *9*, 4939. https://doi.org/10.1038/s41467-018-06885-9.

233. Harmsen, M.J.H.M., Van Dorst, P., Van Vuuren, D.P., Van Den Berg, M., Van Dingenen, R., and Klimont, Z. (2020). Co-benefits of black carbon mitigation for climate and air quality. Climatic Change *163*, 1519–1538. https://doi.org/10.1007/s10584-020-02800-8.

234. Saari, R.K., Selin, N.E., Rausch, S., and Thompson, T.M. (2015). A self-consistent method to assess air quality co-benefits from U.S. climate policies. Journal of the Air & Waste Management Association *65*, 74–89. https://doi.org/10.1080/10962247.2014.959139.

235. Shindell, D.T., Lee, Y., and Faluvegi, G. (2016). Climate and health impacts of US emissions reductions consistent with 2 °C. Nature Clim Change *6*, 503–507. https://doi.org/10.1038/nclimate2935.

236. Smith, S.J., and Mizrahi, A. (2013). Near-term climate mitigation by short-lived forcers. Proc. Natl. Acad. Sci. U.S.A. *110*, 14202–14206. https://doi.org/10.1073/pnas.1308470110.

237. Shindell, D., Borgford-Parnell, N., Brauer, M., Haines, A., Kuylenstierna, J.C.I., Leonard, S.A., Ramanathan, V., Ravishankara, A., Amann, M., and Srivastava, L. (2017). A climate policy pathway for near- and long-term benefits. Science *356*, 493–494. https://doi.org/10.1126/science.aak9521.

238. Schraufnagel, D.E., Balmes, J.R., De Matteis, S., Hoffman, B., Kim, W.J., Perez-Padilla, R., Rice, M., Sood, A., Vanker, A., and Wuebbles, D.J. (2019). Health Benefits of Air Pollution Reduction. Annals of the American Thoracic Society *16*, 1478–1487. https://doi.org/10.1513/AnnalsATS.201907-538CME.

239. Markandya, A., Sampedro, J., Smith, S.J., Van Dingenen, R., Pizarro-Irizar, C., Arto, I., and González-Eguino, M. (2018). Health co-benefits from air pollution and mitigation costs of the Paris Agreement: a modelling study. The Lancet Planetary Health *2*, e126–e133. https://doi.org/10.1016/S2542-5196(18)30029-9.

240. Pierrehumbert, R.T. (2014). Short-Lived Climate Pollution. Annu. Rev. Earth Planet. Sci. *42*, 341–379. https://doi.org/10.1146/annurev-earth-060313-054843.

241. Fakhruddin, B. (Shm), Gluckman, P., Bardsley, A., Griffiths, G., and McElroy, A. (2021). Creating resilient communities with medium-range hazard warning systems. Progress in Disaster Science *12*, 100203. https://doi.org/10.1016/j.pdisas.2021.100203.

242. ILO (2017). Indigenous peoples and climate change: From victims to change agents through decent work (International Labour Organisation).

243. UNDRR (2022). Early warnings for all | UNDRR. http://www.undrr.org/early-warnings-for-all.

244. UNISDR (2015). Sendai Framework for Disaster Risk Reduction 2015 – 2030. UNISDR/GE/2015—ICLUX EN5000 1st edition.

245. Khan, S., Mishra, J.L., Lin, K.E., and Doyle, E.E.H. (2017). Rethinking communication in risk interpretation and action. Nat Hazards *88*, 1709–1726. https://doi.org/10.1007/s11069-017-2942-z.

246. Liu, N., Bouzembrak, Y., Van Den Bulk, L.M., Gavai, A., Van Den Heuvel, L.J., and Marvin, H.J.P. (2022). Automated food safety early warning system in the dairy supply chain using machine learning. Food Control *136*, 108872. https://doi.org/10.1016/j.foodcont.2022.108872.

247. Newell, R. (2023). The climate-biodiversity-health nexus: a framework for integrated community sustainability planning in the Anthropocene. Front. Clim. *5*, 1177025. https://doi.org/10.3389/fclim.2023.1177025.

248. WMO (2023). Increasing water hazards demand better early warnings (World Meteorological Organization).

249. WHO (2018). Climate change and health in small island developing states. A WHO special initiative. Licence: CC BY-NC-SA 3.0 IGO (World Health Organzation).

250. UNDRR and WMO (2022). Global status of multi-hazard early warning (United Nations Office for Disaster Risk Reduction).

251. OECD (2022). Aggregate Trends of Climate Finance Provided and Mobilised by Developed Countries in 2013-2020.

252. UNFCCC (2021). Glasgow Climate Pact. Conference of the Parties Twenty-sixth session. Glasgow, 31 October to 12 November 2021. FCCC/CP/2021/L.13.

253. UNFCCC (2023). Decision 4/CP.28. Long-term climate finance.

254. UNEP (2023). Underfinanced, Underprepared - Inadequate investment and planning on climate adaptation leaves world exposed.

255. Carty, T., Kowalzig, J., and Zagema, B. (2020). Climate Finance Shadow Report 2020: Assessing progress towards the $100 billion commitment (Oxfam) https://doi.org/10.21201/2020.6621.

256. Markandya, A., and González-Eguino, M. (2019). Integrated Assessment for Identifying Climate Finance Needs for Loss and Damage: A Critical Review. In Loss and Damage from Climate Change: Concepts, Methods and Policy Options Climate Risk Management, Policy and Governance., R. Mechler, L. M. Bouwer, T. Schinko, S. Surminski, and J. Linnerooth-Bayer, eds. (Springer International Publishing), pp. 343–362. https://doi.org/10.1007/978-3-319-72026-5_14.

257. Secretariat of CBD (2022). Kunming-Montreal Global Biodiversity Framework.

258. Dempsey, J., Irvine-Broque, A., Bigger, P., Christiansen, J., Muchhala, B., Nelson, S., Rojas-Marchini, F., Shapiro-Garza, E., Schuldt, A., and DiSilvestro, A. (2022). Biodiversity targets will not be met without debt and tax justice. Nat Ecol Evol *6*, 237–239. https://doi.org/10.1038/s41559-021-01619-5.

259. Sharman, N. (2022). Inter-State climate technology transfer under the UNFCCC: A benefit-sharing approach. Review of European, Comparative & International Environmental Law *31*, 435–446. https://doi.org/10.1111/reel.12454.

260. Weko, S., and Goldthau, A. (2022). Bridging the low-carbon technology gap? Assessing energy initiatives for the Global South. Energy Policy *169*, 113192. https://doi.org/10.1016/j.enpol.2022.113192.

261. Zhou, C. (2019). Can intellectual property rights within climate technology transfer work for the UNFCCC and the Paris Agreement? Int Environ Agreements *19*, 107–122. https://doi.org/10.1007/s10784-018-09427-2.

262. Lema, R., Fu, X., and Rabellotti, R. (2021). Green windows of opportunity: latecomer development in the age of transformation toward sustainability. Industrial and Corporate Change *29*, 1193–1209. https://doi.org/10.1093/icc/dtaa044.

263. Nightingale, A.J., Eriksen, S., Taylor, M., Forsyth, T., Pelling, M., Newsham, A., Boyd, E., Brown, K., Harvey, B., Jones, L., et al. (2020). Beyond Technical Fixes: climate solutions and the great derangement. Climate and Development *12*, 343–352. https://doi.org/10.1080/17565529.2019.1624495.

264. Ockwell, D., and Byrne, R. (2016). Improving technology transfer through national systems of innovation: climate relevant innovation-system builders (CRIBs). Climate Policy *16*, 836–854. https://doi.org/10.1080/14693062.2015.1052958.

265. Bezner Kerr, R., Postigo, J.C., Smith, P., Cowie, A., Singh, P.K., Rivera-Ferre, M., Tirado-von Der Pahlen, M.C., Campbell, D., and Neufeldt, H. (2023). Agroecology as a transformative approach to tackle climatic, food, and ecosystemic crises. Current Opinion in Environmental Sustainability *62*, 101275. https://doi.org/10.1016/j.cosust.2023.101275.

266. HLPE (2019). Agroecological and other innovative approaches for sustainable agriculture and food systems that enhance food security and nutrition. A report by the High Level Panel of Experts on Food Security and Nutrition of the Committee on World Food Security (High Level Panel of Experts, Committee on World Food Security).

267. Aguilera, E., Díaz-Gaona, C., García-Laureano, R., Reyes-Palomo, C., Guzmán, G.I., Ortolani, L., Sánchez-Rodríguez, M., and Rodríguez-Estévez, V. (2020). Agroecology for adaptation to climate change and resource depletion in the Mediterranean region. A review. Agricultural Systems *181*, 102809. https://doi.org/10.1016/j.agsy.2020.102809.

268. IPCC (2022). Climate Change 2022: Impacts, adaptation and vulnerability: Contribution of Working Group II to the Sixth Assessment Report of the Intergovernmental Panel on Climate Change H.-O. Pörtner, D.C. Roberts, M. Tignor, E.S. Poloczanska, K. Mintenbeck, A. Alegría, M. Craig, S. Langsdorf, S. Löschke, V. Möller, et al., eds. (Cambridge University Press).

269. Wezel, A., Herren, B.G., Kerr, R.B., Barrios, E., Gonçalves, A.L.R., and Sinclair, F. (2020). Agroecological principles and elements and their implications for transitioning to sustainable food systems. A review. Agron. Sustain. Dev. *40*, 40. https://doi.org/10.1007/s13593-020-00646-z.

270. Mohamed, A., DeClerck, F., Verburg, P.H., Obura, D., Abrams, J.F., Zafra-Calvo, N., Rocha, J., Estrada-Carmona, N., Fremier, A., Jones, S.K., et al. (2022). Biosphere functional integrity for people and Planet. Preprint at Ecology, https://doi.org/10.1101/2022.06.24.497294 https://doi.org/10.1101/2022.06.24.497294.

271. Chapman, M., Walker, W.S., Cook-Patton, S.C., Ellis, P.W., Farina, M., Griscom, B.W., and Baccini, A. (2020). Large climate mitigation potential from adding trees to agricultural lands. Global Change Biology *26*, 4357–4365. https://doi.org/10.1111/gcb.15121.

272. Kuyah, S., Whitney, C.W., Jonsson, M., Sileshi, G.W., Öborn, I., Muthuri, C.W., and Luedeling, E. (2019). Agroforestry delivers a win-win solution for ecosystem services in sub-Saharan Africa. A meta-analysis. Agron. Sustain. Dev. *39*, 47. https://doi.org/10.1007/s13593-019-0589-8.

273. Staton, T., Breeze, T.D., Walters, R.J., Smith, J., and Girling, R.D. (2022). Productivity, biodiversity trade-offs, and farm income in an agroforestry versus an arable system. Ecological Economics *191*, 107214. https://doi.org/10.1016/j.ecolecon.2021.107214.

274. Zinngrebe, Y., Borasino, E., Chiputwa, B., Dobie, P., Garcia, E., Gassner, A., Kihumuro, P., Komarudin, H., Liswanti, N., Makui, P., et al. (2020). Agroforestry governance for operationalising the landscape approach: connecting conservation and farming actors. Sustain Sci *15*, 1417–1434. https://doi.org/10.1007/s11625-020-00840-8.

275. Dawson, I.K., Leakey, R., Clement, C.R., Weber, J.C., Cornelius, J.P., Roshetko, J.M., Vinceti, B., Kalinganire, A., Tchoundjeu, Z., Masters, E., et al. (2014). The management of tree genetic resources and the livelihoods of rural communities in the tropics: Non-timber forest products, smallholder agroforestry practices and tree commodity crops. Forest Ecology and Management *333*, 9–21. https://doi.org/10.1016/j.foreco.2014.01.021.

276. Do, H., Luedeling, E., and Whitney, C. (2020). Decision analysis of agroforestry options reveals adoption risks for resource-poor farmers. Agron. Sustain. Dev. *40*, 20. https://doi.org/10.1007/s13593-020-00624-5.

277. Rosenstock, T.S., Dawson, I.K., Aynekulu, E., Chomba, S., Degrande, A., Fornace, K., Jamnadass, R., Kimaro, A., Kindt, R., Lamanna, C., et al. (2019). A Planetary Health Perspective on Agroforestry in Sub-Saharan Africa. One Earth *1*, 330–344. https://doi.org/10.1016/j.oneear.2019.10.017.

278. Tschora, H., and Cherubini, F. (2020). Co-benefits and trade-offs of agroforestry for climate change mitigation and other sustainability goals in West Africa. Global Ecology and Conservation *22*, e00919. https://doi.org/10.1016/j.gecco.2020.e00919.

279. Ollinaho, O.I., and Kröger, M. (2021). Agroforestry transitions: The good, the bad and the ugly. Journal of Rural Studies *82*, 210–221. https://doi.org/10.1016/j.jrurstud.2021.01.016.

280. Xiao, L., Kuhn, N.J., Zhao, R., and Cao, L. (2021). Net effects of conservation agriculture principles on sustainable land use: A synthesis. Global Change Biology *27*, 6321–6330. https://doi.org/10.1111/gcb.15906.

281. Kaarakka, L., Cornett, M., Domke, G., Ontl, T., and Dee, L.E. (2021). Improved forest management as a natural climate solution: A review. Ecol Sol and Evidence *2*, e12090. https://doi.org/10.1002/2688-8319.12090.

282. Anderson, C.M., DeFries, R.S., Litterman, R., Matson, P.A., Nepstad, D.C., Pacala, S., Schlesinger, W.H., Shaw, M.R., Smith, P., Weber, C., et al. (2019). Natural climate solutions are not enough. Science *363*, 933–934. https://doi.org/10.1126/science.aaw2741.

283. MacDicken, K.G., Sola, P., Hall, J.E., Sabogal, C., Tadoum, M., and De Wasseige, C. (2015). Global progress toward sustainable forest management. Forest Ecology and Management *352*, 47–56. https://doi.org/10.1016/j.foreco.2015.02.005.

284. Roe, S., Streck, C., Obersteiner, M., Frank, S., Griscom, B., Drouet, L., Fricko, O., Gusti, M., Harris, N., Hasegawa, T., et al. (2019). Contribution of the land sector to a 1.5 °C world. Nature Climate Change *9*, 817–828. https://doi.org/10.1038/s41558-019-0591-9.

285. Daigneault, A., and Favero, A. (2021). Global forest management, carbon sequestration and bioenergy supply under alternative shared socioeconomic pathways. Land Use Policy *103*, 105302. https://doi.org/10.1016/j.landusepol.2021.105302.

286. Locatelli, B., Pavageau, C., Pramova, E., and Di Gregorio, M. (2015). Integrating climate change mitigation and adaptation in agriculture and forestry: opportunities and trade-offs: Integrating climate change mitigation and adaptation in agriculture and forestry. Wiley Interdisciplinary Reviews: Climate Change *6*, 585–598. https://doi.org/10.1002/wcc.357.

287. Humpenöder, F., Popp, A., Schleussner, C.-F., Orlov, A., Windisch, M.G., Menke, I., Pongratz, J., Havermann, F., Thiery, W., Luo, F., et al. (2022). Overcoming global inequality is critical for land-based mitigation in line with the Paris Agreement. Nat Commun *13*, 7453. https://doi.org/10.1038/s41467-022-35114-7.

288. Erbaugh, J.T., Pradhan, N., Adams, J., Oldekop, J.A., Agrawal, A., Brockington, D., Pritchard, R., and Chhatre, A. (2020). Global forest restoration and the importance of prioritizing local communities. Nat Ecol Evol *4*, 1472–1476. https://doi.org/10.1038/s41559-020-01282-2.

289. Lenzi, D., Jakob, M., Honegger, M., Droege, S., Heyward, J.C., and Kruger, T. (2021). Equity implications of net zero visions. Climatic Change *169*, 20. https://doi.org/10.1007/s10584-021-03270-2.

290. Börner, J., Schulz, D., Wunder, S., and Pfaff, A. (2020). The Effectiveness of Forest Conservation Policies and Programs. Annu. Rev. Resour. Econ. *12*, 45–64. https://doi.org/10.1146/annurev-resource-110119-025703.

291. Karsenty, A., and Salau, S. (2023). Fiscal Incentives for Improved Forest Management and Deforestation-Free Agricultural Commodities in Central and West Africa. International Forestry Review *25*, 27–38. https://doi.org/10.1505/146554823836838691.

292. Charnley, S., Frey, G.E., and Makala, J. (2022). Community forests, timber production, and certification: success factors in the African context. Ecology and Society *27*. https://doi.org/10.5751/ES-13101-270306.

293. Nepstad, D., Soares-Filho, B.S., Merry, F., Lima, A., Moutinho, P., Carter, J., Bowman, M., Cattaneo, A., Rodrigues, H., Schwartzman, S., et al. (2009). The End of Deforestation in the Brazilian Amazon. Science *326*, 1350–1351. https://doi.org/10.1126/science.1182108.

294. Nepstad, D., McGrath, D., Stickler, C., Alencar, A., Azevedo, A., Swette, B., Bezerra, T., DiGiano, M., Shimada, J., Seroa Da Motta, R., et al. (2014). Slowing Amazon deforestation through public policy and interventions in beef and soy supply chains. Science *344*, 1118–1123. https://doi.org/10.1126/science.1248525.

295. Vale, M.M., Berenguer, E., Argollo De Menezes, M., Viveiros De Castro, E.B., Pugliese De Siqueira, L., and Portela, R.D.C.Q. (2021). The COVID-19 pandemic as an opportunity to weaken environmental protection in Brazil. Biological Conservation *255*, 108994. https://doi.org/10.1016/j.biocon.2021.108994.

296. Nellemann, C., Corcoran, E., Duarte, C.M., Valdes, L., De Young, C., Fonseca, L., and Grimsditch, G. eds. (2009). Blue carbon: The role of healthy oceans in binding carbon: A rapid response assessment (GRID-Arendal).

297. Giri, C., Ochieng, E., Tieszen, L.L., Zhu, Z., Singh, A., Loveland, T., Masek, J., and Duke, N. (2011). Status and distribution of mangrove forests of the world using earth observation satellite data. Global Ecology and Biogeography *20*, 154–159. https://doi.org/10.1111/j.1466-8238.2010.00584.x.

298. Duarte, C.M., Losada, I.J., Hendriks, I.E., Mazarrasa, I., and Marbà, N. (2013). The role of coastal plant communities for climate change mitigation and adaptation. Nature Clim Change *3*, 961–968. https://doi.org/10.1038/nclimate1970.

299. Mason, V.G., Burden, A., Epstein, G., Jupe, L.L., Wood, K.A., and Skov, M.W. (2023). Blue carbon benefits from global saltmarsh restoration. Global Change Biology *29*, 6517–6545. https://doi.org/10.1111/gcb.16943.

300. Duarte, C.M., Bruhn, A., and Krause-Jensen, D. (2021). A seaweed aquaculture imperative to meet global sustainability targets. Nat Sustain *5*, 185–193. https://doi.org/10.1038/s41893-021-00773-9.

301. Diatta, C.S., Diouf, M., Sow, A.A., and Karibuhoye, C. (2020). The Joola civilization and the management of mangroves in Lower Casamance, Senegal. ethnoecologie *17*. https://doi.org/10.4000/ethnoecologie.5855.

302. Islam, S.N., and Yahya, U.A.A.B. (2017). Impacts of Coastal Land Use Changes on Mangrove Wetlands at Sungai Mangsalut Basin in Brunei Darussalam. In Coastal Wetlands: Alteration and Remediation Coastal Research Library., C. W. Finkl and C. Makowski, eds. (Springer International Publishing), pp. 133–157. https://doi.org/10.1007/978-3-319-56179-0_4.

303. Jalais, A. (2014). Forest of Tigers: People, Politics and Environment in the Sundarbans 0 ed. (Routledge India) https://doi.org/10.4324/9780203085516.

304. Uddin, Md.S., De Ruyter Van Steveninck, E., Stuip, M., and Shah, M.A.R. (2013). Economic valuation of provisioning and cultural services of a protected mangrove ecosystem: A case study on Sundarbans Reserve Forest, Bangladesh. Ecosystem Services *5*, 88–93. https://doi.org/10.1016/j.ecoser.2013.07.002.

305. Walters, B.B., Rönnbäck, P., Kovacs, J.M., Crona, B., Hussain, S.A., Badola, R., Primavera, J.H., Barbier, E., and Dahdouh-Guebas, F. (2008). Ethnobiology, socio-economics and management of mangrove forests: A review. Aquatic Botany *89*, 220–236. https://doi.org/10.1016/j.aquabot.2008.02.009.

306. Chuku, E.O., Effah, E., Adotey, J., Abrokwah, S., Adade, R., Okyere, I., Aheto, D.W., Kent, K., Osei, I.K., Omogbemi, E.D., et al. (2022). Spotlighting Women-Led Fisheries Livelihoods Toward Sustainable Coastal Governance: The Estuarine and Mangrove Ecosystem Shellfisheries of West Africa. Front. Mar. Sci. *9*, 884715. https://doi.org/10.3389/fmars.2022.884715.

307. Crow, B., and Carney, J. (2013). Commercializing Nature: Mangrove Conservation and Female Oyster Collectors in The Gambia. Antipode *45*, 275–293. https://doi.org/10.1111/j.1467-8330.2012.01015.x.

308. Friedlander, A.M., Ballesteros, E., Goodell, W., Hüne, M., Muñoz, A., Salinas-de-León, P., Velasco-Charpentier, C., and Sala, E. (2021). Marine communities of the newly created Kawésqar National Reserve, Chile: From glaciers to the Pacific Ocean. PLoS ONE *16*, e0249413. https://doi.org/10.1371/journal.pone.0249413.

309. Unsworth, R.K.F., Cullen-Unsworth, L.C., Jones, B.L.H., and Lilley, R.J. (2022). The planetary role of seagrass conservation. Science *377*, 609–613. https://doi.org/10.1126/science.abq6923.

310. Friess, D.A., Gatt, Y.M., Ahmad, R., Brown, B.M., Sidik, F., and Wodehouse, D. (2022). Achieving ambitious mangrove restoration targets will need a transdisciplinary and evidence-informed approach. One Earth *5*, 456–460. https://doi.org/10.1016/j.oneear.2022.04.013.

311. Goodwin, S., Olazabal, M., Castro, A.J., and Pascual, U. (2023). Global mapping of urban nature-based solutions for climate change adaptation. Nat Sustain *6*, 458–469. https://doi.org/10.1038/s41893-022-01036-x.

312. Ferreira, C.S.S., Potočki, K., Kapović-Solomun, M., and Kalantari, Z. (2021). Nature-Based Solutions for Flood Mitigation and Resilience in Urban Areas. In Nature-Based Solutions for Flood Mitigation The Handbook of Environmental Chemistry., C. S. S. Ferreira, Z. Kalantari, T. Hartmann, and P. Pereira, eds. (Springer International Publishing), pp. 59–78. https://doi.org/10.1007/698_2021_758.

313. Marando, F., Heris, M.P., Zulian, G., Udías, A., Mentaschi, L., Chrysoulakis, N., Parastatidis, D., and Maes, J. (2022). Urban heat island mitigation by green infrastructure in European Functional Urban Areas. Sustainable Cities and Society *77*, 103564. https://doi.org/10.1016/j.scs.2021.103564.

314. Richards, D.R., Belcher, R.N., Carrasco, L.R., Edwards, P.J., Fatichi, S., Hamel, P., Masoudi, M., McDonnell, M.J., Peleg, N., and Stanley, M.C. (2022). Global variation in contributions to human well-being from urban vegetation ecosystem services. One Earth *5*, 522–533. https://doi.org/10.1016/j.oneear.2022.04.006.

315. Huang, Y., Tian, Z., Ke, Q., Liu, J., Irannezhad, M., Fan, D., Hou, M., and Sun, L. (2020). Nature-based solutions for urban pluvial flood risk management. WIREs Water *7*, e1421. https://doi.org/10.1002/wat2.1421.

316. Rojas-Rueda, D., De Nazelle, A., Andersen, Z.J., Braun-Fahrländer, C., Bruha, J., Bruhova-Foltynova, H., Desqueyroux, H., Praznoczy, C., Ragettli, M.S., Tainio, M., et al. (2016). Health Impacts of Active Transportation in Europe. PLoS ONE *11*, e0149990. https://doi.org/10.1371/journal.pone.0149990.

317. Chatzimentor, A., Apostolopoulou, E., and Mazaris, A.D. (2020). A review of green infrastructure research in Europe: Challenges and opportunities. Landscape and Urban Planning *198*, 103775. https://doi.org/10.1016/j.landurbplan.2020.103775.

318. Filazzola, A., Shrestha, N., and MacIvor, J.S. (2019). The contribution of constructed green infrastructure to urban biodiversity: A synthesis and meta-analysis. Journal of Applied Ecology *56*, 2131–2143. https://doi.org/10.1111/1365-2664.13475.

319. Xie, L., and Bulkeley, H. (2020). Nature-based solutions for urban biodiversity governance. Environmental Science & Policy *110*, 77–87. https://doi.org/10.1016/j.envsci.2020.04.002.

320. Key, I.B., Smith, A.C., Turner, B., Chausson, A., Girardin, C.A.J., Macgillivray, M., and Seddon, N. (2022). Biodiversity outcomes of nature-based solutions for climate change adaptation: Characterising the evidence base. Front. Environ. Sci. *10*, 905767. https://doi.org/10.3389/fenvs.2022.905767.

321. Dorr, E., Hawes, J.K., Goldstein, B., Fargue-Lelièvre, A., Fox-Kämper, R., Specht, K., Fedeńczak, K., Caputo, S., Cohen, N., Poniży, L., et al. (2023). Food production and resource use of urban farms and gardens: a five-country study. Agron. Sustain. Dev. *43*, 18. https://doi.org/10.1007/s13593-022-00859-4.

322. Hume, C., Grieger, J.A., Kalamkarian, A., D'Onise, K., and Smithers, L.G. (2022). Community gardens and their effects on diet, health, psychosocial and community outcomes: a systematic review. BMC Public Health *22*, 1247. https://doi.org/10.1186/s12889-022-13591-1.

323. Puigdueta, I., Aguilera, E., Cruz, J.L., Iglesias, A., and Sanz-Cobena, A. (2021). Urban agriculture may change food consumption towards low carbon diets. Global Food Security *28*, 100507. https://doi.org/10.1016/j.gfs.2021.100507.

324. Anguelovski, I., Connolly, J.J.T., Cole, H., Garcia-Lamarca, M., Triguero-Mas, M., Baró, F., Martin, N., Conesa, D., Shokry, G., Del Pulgar, C.P., et al. (2022). Green gentrification in European and North American cities. Nat Commun *13*, 3816. https://doi.org/10.1038/s41467-022-31572-1.

325. Kabisch, N., Stadler, J., Korn, H., and Bonn, A. (2016). Nature-based solutions to climate change mitigation and adaptation in urban areas (Federal Agency for Nature Conservation).

326. Kabisch, N., Frantzeskaki, N., and Hansen, R. (2022). Principles for urban nature-based solutions. Ambio *51*, 1388–1401. https://doi.org/10.1007/s13280-021-01685-w.

327. Van Zanten, B.T., Gutierrez Goizueta, G., Brander, L.M., Gonzalez Reguero, B., Griffin, R., Macleod, K.K., Alves Beloqui, A.I., Midgley, A., Herrera Garcia, L.D., and Jongman, B. (2023). Assessing the Benefits and Costs of Nature-Based Solutions for Climate Resilience: A Guideline for Project Developers (World Bank) https://doi.org/10.1596/39811.

328. Laine, J.E., Huybrechts, I., Gunter, M.J., Ferrari, P., Weiderpass, E., Tsilidis, K., Aune, D., Schulze, M.B., Bergmann, M., Temme, E.H.M., et al. (2021). Co-benefits from sustainable dietary shifts for population and environmental health: an assessment from a large European cohort study. The Lancet Planetary Health *5*, e786–e796. https://doi.org/10.1016/S2542-5196(21)00250-3.

329. Springmann, M., Clark, M., Mason-D'Croz, D., Wiebe, K., Bodirsky, B.L., Lassaletta, L., De Vries, W., Vermeulen, S.J., Herrero, M., Carlson, K.M., et al. (2018). Options for keeping the food system within environmental limits. Nature *562*, 519–525. https://doi.org/10.1038/s41586-018-0594-0.

330. Springmann, M., Spajic, L., Clark, M.A., Poore, J., Herforth, A., Webb, P., Rayner, M., and Scarborough, P. (2020). The healthiness and sustainability of national and global food based dietary guidelines: modelling study. BMJ, m2322. https://doi.org/10.1136/bmj.m2322.

331. Willett, W., Rockström, J., Loken, B., Springmann, M., Lang, T., Vermeulen, S., Garnett, T., Tilman, D., DeClerck, F., Wood, A., et al. (2019). Food in the Anthropocene: the EAT–Lancet Commission on healthy diets from sustainable food systems. The Lancet *393*, 447–492. https://doi.org/10.1016/S0140-6736(18)31788-4.

332. Williams, B.A., Venter, O., Allan, J.R., Atkinson, S.C., Rehbein, J.A., Ward, M., Di Marco, M., Grantham, H.S., Ervin, J., Goetz, S.J., et al. (2020). Change in Terrestrial Human Footprint Drives Continued Loss of Intact Ecosystems. One Earth *3*, 371–382. https://doi.org/10.1016/j.oneear.2020.08.009.

333. Belgacem, W., Mattas, K., Arampatzis, G., and Baourakis, G. (2021). Changing Dietary Behavior for Better Biodiversity Preservation: A Preliminary Study. Nutrients *13*, 2076. https://doi.org/10.3390/nu13062076.

334. Milner, J., Joy, E.J.M., Green, R., Harris, F., Aleksandrowicz, L., Agrawal, S., Smith, P., Haines, A., and Dangour, A.D. (2017). Projected health effects of realistic dietary changes to address freshwater constraints in India: a modelling study. The Lancet Planetary Health *1*, e26–e32. https://doi.org/10.1016/S2542-5196(17)30001-3.

335. Tirado, M.C., Vivero-Pol, J.L., Bezner Kerr, R., and Krishnamurthy, K. (2022). Feasibility and Effectiveness Assessment of Multi-Sectoral Climate Change Adaptation for Food Security and Nutrition. Curr Clim Change Rep *8*, 35–52. https://doi.org/10.1007/s40641-022-00181-x.

336. Jalava, M., Kummu, M., Porkka, M., Siebert, S., and Varis, O. (2014). Diet change—a solution to reduce water use? Environ. Res. Lett. *9*, 074016. https://doi.org/10.1088/1748-9326/9/7/074016.

337. Springmann, M., Mason-D'Croz, D., Robinson, S., Garnett, T., Godfray, H.C.J., Gollin, D., Rayner, M., Ballon, P., and Scarborough, P. (2016). Global and regional health effects of future food production under climate change: a modelling study. The Lancet *387*, 1937–1946. https://doi.org/10.1016/S0140-6736(15)01156-3.

338. Semba, R.D., De Pee, S., Kim, B., McKenzie, S., Nachman, K., and Bloem, M.W. (2020). Adoption of the 'planetary health diet' has different impacts on countries' greenhouse gas emissions. Nat Food *1*, 481–484. https://doi.org/10.1038/s43016-020-0128-4.

339. Hirvonen, K., Bai, Y., Headey, D., and Masters, W.A. (2020). Affordability of the EAT–Lancet reference diet: a global analysis. The Lancet Global Health *8*, e59–e66. https://doi.org/10.1016/S2214-109X(19)30447-4.

340. Herrero, M., Hugas, M., Lele, U., Wirakartakusumah, A., and Torero, M. (2023). A Shift to Healthy and Sustainable Consumption Patterns. In Science and Innovations for Food Systems Transformation, J. Von Braun, K. Afsana, L. O. Fresco, and M. H. A. Hassan, eds. (Springer International Publishing), pp. 59–85. https://doi.org/10.1007/978-3-031-15703-5_5.

341. Joly, C.A., Moraes, A.R.D., Speglich, É., Berro, G.B., and Vieira, S.A. (2023). Amazon Dialogues: Contributions to the Debate About Sustainability and Inclusion (RIMA Editora) https://doi.org/10.55333/rima-978-65-84811-41-6.
